# The First International Consortium for Health Outcomes Measurement (ICHOM) Standard Dataset for Reporting Outcomes in Heart Valve Disease: Moving From Device- to Patient-Centered Outcomes

**DOI:** 10.1177/15569845241269309

**Published:** 2025-02-19

**Authors:** Emmanuel Lansac, Kevin M. Veen, Andria Joseph, Paula Blancarte Jaber, Frieda Sossi, Zofia Das-Gupta, Suleman Aktaa, J. Rafael Sádaba, Vinod H. Thourani, Gry Dahle, Wilson Y. Szeto, Faisal Bakaeen, Elena Aikawa, Frederick J. Schoen, Evaldas Girdauskas, Aubrey Almeida, Andreas Zuckermann, Bart Meuris, John Stott, Jolanda Kluin, Ruchika Meel, Wil Woan, Daniel Colgan, Hani Jneid, Husam Balkhy, Molly Szerlip, Ourania Preventza, Pinak Shah, Vera H. Rigolin, Silvana Medica, Philip Holmes, Marta Sitges, Philippe Pibarot, Erwan Donal, Rebecca T. Hahn, Johanna J.M. Takkenberg

**Affiliations:** 1 Hôpital Pitié-Salpêtrière, Sorbonne Université, Paris, France; 2 Department of Cardiothoracic Surgery, Erasmus MC, Rotterdam, The Netherlands; 3 International Consortium for Health Outcomes Measurement, London, UK; 4 Department of Cardiology, Leeds Teaching Hospitals NHS Foundation Trust and Leeds Institute of Cardiovascular and Metabolic Medicine, UK; 5 Department of Cardiac Surgery, Navarra University Hospital, Pamplona, Spain; 6 Department of Cardiovascular Surgery, Marcus Valve Center, Piedmont Heart Institute, Atlanta, GA, USA; 7 Department of Cardiothoracic Surgery, Oslo University Hospital, Norway; 8 Division of Cardiothoracic Surgery, University of Pennsylvania, Philadelphia, PA, USA; 9 Department of Thoracic and Cardiovascular Surgery, Cleveland Clinic Main Campus, OH, USA; 10 Department of Medicine, Division of Cardiovascular Medicine, Brigham and Women’s Hospital, Harvard Medical School, Boston, MA, USA; 11 Department of Pathology, Brigham and Women’s Hospital, Harvard Medical School, Boston, MA, USA; 12 Department of Cardiothoracic Surgery, Augsburg University Hospital, Germany; 13 Monash Medical Centre, Melbourne, Australia; 14 Department of Cardiac Surgery, Medical University of Vienna, Austria; 15 Department of Cardiovascular Sciences, KU Leuven, Belgium; 16 Heart Valve Voice Canada, Toronto, ON, Canada; 17 Chris Hani Baragwanath Hospital, Department of Internal Medicine, Faculty of Health Sciences, School of Clinical Medicine, University of the Witwatersrand, Johannesburg, South Africa; 18 Heart Valve Voice, Manchester, UK; 19 Independent patient representative; 20 University of Texas Medical Branch, Galveston, TX, USA; 21 Department of Surgery, Biological Sciences Division, University of Chicago Medicine, IL, USA; 22 Baylor Scott & White The Heart Hospital Plano, TX, USA; 23 Division of Cardiothoracic Surgery, University of Virginia, Charlottesville, VA, USA; 24 Heart and Vascular Center, Brigham and Women’s Hospital, Boston, MA, USA; 25 Northwestern University Feinberg School of Medicine, Chicago, IL, USA; 26 Hospital Clínic Cardiovascular Institute, Barcelona, Spain; 27 Quebec Heart and Lung Institute, Quebec City, QC, Canada; 28 Cardiology Department, University Hospital of Rennes, France; 29 Department of Cardiology, Columbia Structural Heart & Valve Center, Columbia University, New York, NY, USA

**Keywords:** AHA Scientific Statements, aortic valve, heart valve diseases, mitral valve, outcome and process assessment, health care, patient outcome assessment, patient reported outcome measures, tricuspid valve

## Abstract

**Objective:**

Globally significant variation in treatment and course of heart valve disease (HVD) exists, and outcome measurement is procedure focused instead of patient focused. This article describes the development of a patient-related (International Consortium for Health Outcomes Measurement) standard set of outcomes and case mix to be measured in patients with HVD.

**Methods:**

A multisociety working group was formed that included patient representatives and representatives from scientific cardiology and cardiothoracic surgery societies that publish current guidelines for HVD. The standard set was developed to monitor the patient’s journey from diagnosis to treatment with either a surgical or transcatheter procedure. Candidate clinical and patient-reported outcome measures (PROMs) and case mix were identified through benchmark analyses and systematic reviews. Using an online modified Delphi process, the working group voted on final outcomes/case mix and corresponding definition.

**Results:**

Patients with aortic/mitral/tricuspid valve disease or root/ascending aorta >40 mm were included in the standard set. Patients entered the dataset when the diagnosis of HVD was established, allowing outcome measurement in the preprocedural, periprocedural, and postprocedural phases of patients’ lives. The working group defined 5 outcome domains: vital status, patient-reported outcomes, progression of disease, cardiac function and durability, and complications of treatment. Subsequently, 16 outcome measures, including 2 patient-reported outcomes, were selected to be tracked in patients with HVD. Case-mix variables included demographic factors, demographic variables, echocardiographic variables, heart catheterization variables, and specific details on aortic/mitral/tricuspid valves and their specific interventions.

**Conclusions:**

Through a unique collaborative effort between patients and cardiology and cardiothoracic surgery societies, a standard set of measures for HVD was developed. This dataset focuses on outcome measurement regardless of treatment, moving from procedure- to patient-centered outcomes. Implementation of this dataset will facilitate global standardization of outcome measurement, allow meaningful comparison between health care systems and evaluation of clinical practice guidelines, and eventually improve patient care for those experiencing HVD worldwide.

## Introduction

Heart valve disease (HVD) is a major cause of mortality and quality of life impairment worldwide.^1,2^ The direct costs of aortic valve disease are estimated to be $10.2 billion in the United States.^3^ Aging population and improvements in diagnostic strategies led to an increase in the incidence and prevalence of HVD in recent years, with an anticipated rise in the proportion of people >60 years of age from 10% to 21%.^4^ As a result, it is estimated that the number of patients requiring heart valve interventions will sharply increase in the coming decades.^5^ Although degenerative HVD predominates in high-income countries, rheumatic disease remains the major challenge in low- to middle-income countries.^6^

Many clinical practice guidelines exist on HVD with recommendations for diagnosis, monitoring, and treatment for different settings of the disease.^4,7^ However, tools that standardize the methods by which patient outcomes are reported are lacking, limiting the opportunity to evaluate care quality globally.

Studies pertinent to HVD tend to focus on procedural or device outcomes and less on other (patient-reported) outcomes that are also important to patients. Efforts have been made to standardize clinical outcomes reporting in heart valve research by the Valve Academic Research Consortium (VARC) of experts.^8–10^ These outcome definitions provide an excellent and detailed overview of important end points. However, they have been developed for clinical trials and are designed to evaluate procedural outcomes rather than disease outcomes that are relevant to patients’ physical and mental well-being and longevity of life. Furthermore, because these criteria have not been validated through a multisociety endorsement process, disagreement exists among scientific societies on outcome definitions, limiting proper evaluation of patients’ outcomes worldwide.^11^

These developments called for a globally inclusive standard set of patient-centered outcome measures (referred to as Set herein) for HVD that can facilitate a standardized method for capturing and measuring existing guidelines, as well as patients’ long-term outcomes, regardless of their treatment (surgical and transcatheter procedure). It was developed by an HVD working group from the International Consortium for Health Outcomes Measurement (ICHOM) to monitor the patient’s journey from diagnosis, including potential treatments.

ICHOM, founded in 2012, seeks to promote alignment of outcome measurements globally using standardized outcome measurement. The goal of the HVD working group, which included patient representatives and a multisociety working group, was to develop a Set for HVD to improve patient care and to allow comparison of health care systems and treatment strategies. The HVD working group aimed to balance the amount and complexity of included outcomes with sufficient detail to ensure an implementable set of measures and meaningful comparison.

## Methods

### Working Group Composition

This Set was initiated by the Heart Valve Society, and the HVD working group included representatives from the American Heart Association, American College of Cardiology, the European Association for Cardio-Thoracic Surgery, the European Society of Cardiology, The Society of Thoracic Surgeons, the Australian & New Zealand Society of Cardiac & Thoracic Surgeons, the International Society for Applied Cardiovascular Biology, the International Society for Minimally Invasive Cardiothoracic Surgery, the South African Heart Association, and Heart Valve Voice Canada and UK branches, affiliates of the Global Heart Hub, a cardiovascular disease patient-led organization. In addition, the HVD working group included experts in the field, including epidemiology and public health, patient representatives, and patient advocates. HVD working group members were identified through published works, position in relevant societies, ICHOM’s professional network, or expert recommendations. Industry representatives were deliberately excluded from the working group. In total, 24 members from 12 countries were included. The HVD working group was led by 2 chairs (J.J.M.T. and E.L.) with expertise in HVD, epidemiology, and shared decision-making. A project team was also formed that included project managers (A.J. and P.B.J.) and a research fellow (K.M.V.) to coordinate the process and to provide supporting research efforts.

### Objectives

The primary objective was to develop a Set for patients with HVD (aortic, mitral, and tricuspid) that can be tracked by physicians and health care systems. This Set allows patients and physicians to monitor HVD throughout the patient’s journey. These outcomes are related to survival, valve function, quality of life, physical fitness, and treatment-related outcomes. The secondary aim was to identify a set of case-mix variables (e.g., morbidity) to ensure comparison of outcomes across health care systems and to allow risk adjustment.

### Candidate Case Mix and Outcomes

The project team identified a list of candidate case-mix variables and outcomes using a 3-pillar approach. This approach consisted of (1) a benchmark review of large registries, (2) a data visualization approach to screen commonly used words in all abstracts concerning HVD in MEDLINE, and (3) a systematic search to identify outcomes in literature using an iterative algorithm of selecting new articles until a full saturation of outcomes and case-mix variables was achieved (i.e., when no new outcomes or case mix was identified in successive rounds of article selections). These 3 pillars were combined to obtain a comprehensive list of candidate outcome measures and case-mix variables. The details of these approaches are discussed in an accompanying methodology article.^12^ In cases of PROMs, a separate systematic search was performed that yielded 856 articles, all of which were reviewed. The search for clinical outcome measures and case mix yielded 17,322 articles. The search terms for the clinical and case-mix outcomes and for the PROMs are provided in Supplemental Text 1 and 2.

### Process

The modified Delphi process was used to reach consensus in all major decision areas, including the target population to be covered, the final outcome and case-mix set, definitions of outcomes, and time points of measurements.^13^ In accordance with this method, 11 teleconference calls were organized between December 15, 2020, and April 12, 2022, and multiple surveys among the HVD working group members were used to reach consensus (Supplemental Fig. 1). Before each teleconference, the HVD working group members received the supporting research (e.g., the list with candidate outcomes measures), which was discussed during the teleconference. After the teleconference, a survey was computed to address each discussion point and potential outcomes/case mix or definitions. HVD working group members considered several criteria for selecting the variables, including the frequency of the outcome, the effect of the outcome of patients’ lives, and the feasibility of data collection. For the selections of PROMs, key domains of patients’ lives (e.g., functional, mental health) were considered. If a majority of two-thirds was reached in the HVD working group, the decision was adopted; if this majority was not reached, the topic was discussed further in the next teleconference after another survey. If the threshold was not reached in the second survey, a majority decision was adopted. In cases of specific subjects (e.g., defining valve regurgitations), additional calls with experts were organized.

## Results

### Population

The target population for this Set includes all adult patients (≥18 years of age) with HVD, including the aortic, mitral, or tricuspid valves. Disease of the pulmonary valve was excluded because most of the underlying cause is congenital and a set encompassing congenital heart disease already exists.^14^ Patients may be included in the Set if they have at least moderate aortic and mitral valve stenosis, significant tricuspid valve stenosis, or grade II or higher regurgitation of the aortic, mitral valve, more than moderate tricuspid valve regurgitation, or root/ascending aorta dilatation >40 mm (Table 1). If the patient’s first presentation is at intervention, it was decided that they may be included at the time of the intervention.

**Table 1.** Inclusion Criteria for the Standard Set.

**Valve**	**Hemodynamics**	**Measurement**	**Values**
Aortic	Grade II or higher valve regurgitation	Grade II or higher regurgitation	See Table 3
	Moderate or higher valve stenosis^15^	Peak velocity	≥3.0 m/s
		Mean gradient	≥20 mm Hg
		Aortic valve area	≤1.5 cm^2^
		Indexed aortic valve area	≤0.85 cm^2^/m^2^
		Velocity ratio	≤0.50
	Root/ascending dilatation	Root/ascending diameter	>40 mm
Mitral	Grade II or higher valve regurgitation	Grade II or higher regurgitation	See Table 4
	Moderate or higher valve stenosis^16^	Valve area (specific finding)	≤1.5 cm^2^
		Mean gradient (supportive finding)	≥10.0 mm Hg
Tricuspid	Grade moderate or higher valve regurgitation	Grade moderate or higher regurgitation	See Table 5
	Significant valve stenosis^16^	Mean pressure gradient	≥5.0 mm Hg
		Inflow time-velocity integral	>60 cm
		T_1/2_	≥190 ms
		Valve area	≤1.0 cm^2^

### Candidate Outcome/Case-Mix Screening

To inform the HVD working group of potential outcomes and case mix used in medical literature, the 3 approaches were combined. In total, 6 HVD registries^17–22^ were chosen (Supplemental Table 1), in combination with 4 consensus articles, including the publication by Akins and colleagues^23^ and the VARC-1/2/3 guidelines on reporting^8–10^ as benchmark review. The WordCloud of the machine learning algorithm is presented in Supplemental Figure 2. For outcomes, 150 references were screened until full saturation of candidate outcomes was achieved, encompassing 52 candidate outcomes. For case mix, 125 articles were screened, encompassing 330 case-mix variables. For PROMs, 856 articles were screened, encompassing 60 potential PROMs.

### Outcome Set

**Fig. 1.**
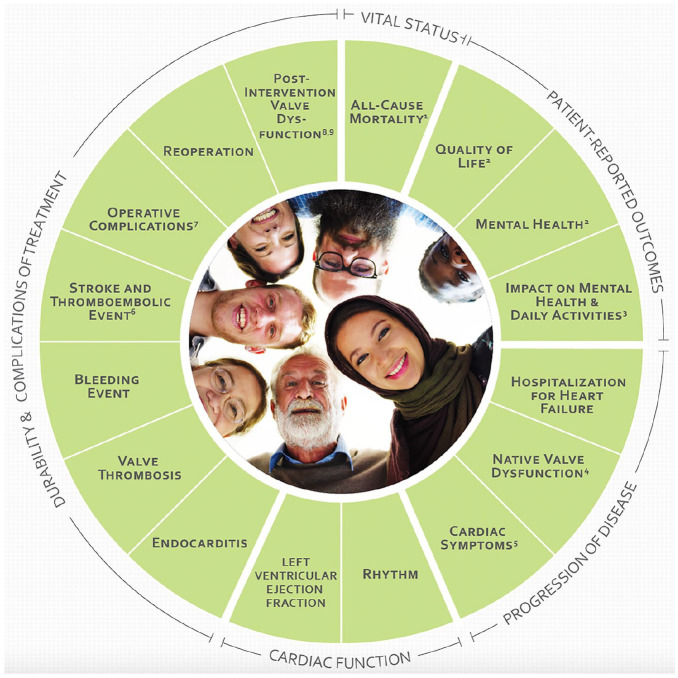
Core domains and included outcomes. (1) Includes early mortality, late mortality, valve-related mortality, and all-cause mortality. (2) Defined by the EQ-5D-5L (EuroQol Group) questionnaire. (3) Defined by the Heart Valve Disease Impact on Daily Life Questionnaire. (4) Includes aortic/mitral/tricuspid valve stenosis and aortic/mitral/tricuspid valve regurgitation. (5) Includes angina pectoris and New York Heart Association functional class. (6) Includes stroke (ischemic/hemorrhagic) and thromboembolic event (noncerebral). (7) Includes conversion to open heart surgery, reoperation for bleeding, periprocedural myocardial infarction, new permanent pacemaker, major/minor vascular complications, and low-cardiac-output syndrome. (8) Includes paravalvular insufficiency and device migration (applicable only to percutaneous devices). (9) Refers to structural deterioration and nonstructural valve dysfunction.

The HVD working group defined 5 domains: vitals status, patient-reported outcomes, progression of disease, cardiac function and durability, and complications of treatment. Subsequently, 16 outcomes were selected to be tracked in the target population (Fig. 1), of which some outcomes measures were further subdivided (Table 2, Table 3).

**Table 2.** Definition of Domains: Vital Status, Patient-Reported Outcomes, Progression of Disease, and Cardiac Function.

**Domain**	**Outcomes/subdivision**	**Definition/questionnaire**
Vital status	Mortality 1. All-cause mortality 2. 30-d mortality 3. Late mortality 4. Valve-related mortality	1. Indicate whether the person has died regardless of cause 2. Mortality after 30 d after a surgical/percutaneous intervention 3. Indicate whether the person has died regardless of cause >30 d after intervention 4. Valve-related mortality is any death caused by structural valve deterioration, nonstructural dysfunction, valve thrombosis, embolism, valve-related bleeding, or prosthetic valve endocarditis; death related to reintervention on the operated valve; or sudden unexplained death.
Patient-reported outcomes	Quality of life	EQ-5D-5L questionnaire^26^
	Mental health	Heart Valve Disease Impact on Daily Life Questionnaire^27^
	Impact on mental health and daily activities	
Progression of disease	Hospitalization for heart failure	Unplanned hospital admission for clinical treatment for heart failure
	Native valve dysfunction 1. Aortic stenosis/regurgitation 2. Mitral stenosis/regurgitation 3. Tricuspid stenosis/regurgitation	1. Measured as continuous maximum transvalvular gradient/velocity and aortic valve area (stenosis) and as grade 1–4 regurgitation (Table 3) 2. Measured as continuous mean transvalvular gradient/velocity and mitral valve area (stenosis) and as grade 1–4 regurgitation (Table 4) 3. Measured as continuous mean transvalvular gradient as grade 1–3 regurgitation (Table 5)
	Cardiac symptoms 1. Dyspnea 2. Angina pectoris	1. NYHA classification^28^ 2. CCS angina classification^29^
Cardiac function	Rhythm	Baseline rhythm of the patient at time of measurement coded as sinus, AF, paced, and other. If a patient has a permanent/temporary pacemaker but has AF/sinus on ECG, AF/sinus should be coded.
	Left ventricular ejection fraction	Left ventricular ejection fraction in percentage; can be measured by 2D/3D echocardiography or MRI

Abbreviations: 2D, 2-dimensional; 3D, 3-dimensional; AF, atrial fibrillation or flutter; CCS, Canadian Cardiovascular Society; MRI, magnetic resonance imaging; NYHA, New York Heart Association.

**Table 3.** Definition of Durability and Complication of Treatment Domain.

**Domain**	**Outcomes/subdivision**	**Definition/questionnaire**
Durability and complication of treatment	Endocarditis	Modified Duke criteria for endocarditis (see Supplemental Reference Guide for extensive criteria)^30^
	Valve thrombosis	Valve thrombosis is any thrombus not caused by infection attached to or near an operated valve that occludes part of the blood flow path, interferes with valve function, or is sufficiently large to warrant treatment
	Bleeding event	Type 1: Overt bleeding that requires medical intervention by a health care professional, leading to hospitalization, an increased level of care, or medical evaluation OR overt bleeding that requires a transfusion of 1 U whole blood/red blood cells Type 2: Overt bleeding that requires a transfusion of 2–4 U whole blood/red blood cells Type 3: Overt bleeding in a critical organ such as intracranial, intraspinal, intraocular, pericardial (associated with hemodynamic compromise/tamponade and necessitating intervention), or intramuscular with compartment syndrome AND/OR overt bleeding causing hypovolemic shock or severe hypotension (systolic blood pressure <90 mm Hg lasting >30 min and not responding to volume resuscitation) or requiring vasopressors or surgery. Overt bleeding requiring a transfusion of 5 U whole blood/red blood cells. Type 4: Overt bleeding leading to death. Should be classified as probable (clinical suspicion) or definite (confirmed by autopsy or imaging). In this case, valve-related mortality should also be documented.
	Stroke and thromboembolic event 1. Stroke (ischemic or hemorrhagic) 2. Noncerebral thromboembolism	1. Acute episode of a focal or global neurological deficit with at least 1 of the following: change in the level of consciousness, hemiplegia, hemiparesis, numbness, or sensory loss affecting 1 side of the body; dysphasia or aphasia; hemianopia; amaurosis fugax; or other neurological signs or symptoms consistent with stroke Stroke: duration of a focal or global neurological deficit ≥24 h OR <24 h if available neuroimaging documents a new hemorrhage or infarct O the neurological deficit results in death. Classified as ischemic and hemorrhagic (see Supplemental Reference Guide for extensive definition) Transient ischemic attack: Transient focal neurological signs or symptoms lasting <24 h presumed to be due to focal brain, spinal cord, or retinal ischemia, but without evidence of acute infarction by neuroimaging or pathology, or with no imaging performed 2. A noncerebral embolic event is an embolus documented operatively, at autopsy, or clinically that produces signs or symptoms attributable to complete or partial obstruction of a noncerebral artery
	Procedural complication 1. Conversion to open heart surgery 2. Reoperation for bleeding 3. Periprocedural myocardial infarction 4. New permanent pacemaker 5. Major/minor vascular complications 6. Low-cardiac output syndrome	1. Need for conversion to open heart surgery during percutaneous/minimally invasive valve interventions 2. In case of surgical intervention, return to the operating room for rethoracotomy/sternotomy; in case of percutaneous intervention, unplanned intervention for the purpose of controlling the bleeding 3. Type 5 MI according the Fourth Universal Definition of Myocardial infarction (see Supplemental Reference Guide for extensive criteria)^24^ 4. Implantation of a new permanent pacemaker; not including pacemaker exchange of previously implanted pacemaker. 5. Any complication related to the device insertion, delivery, and complete removal of all its components (delivery catheter, sheath, guide wire), excluding the actual implantation in the heart. Categorized as major and minor according to the VARC-3 criteria (see Supplemental Reference Guide for extensive criteria).^10^ 6. Need for mechanical circulatory support with IABP, LVAD, or extracorporeal membrane oxygenation during surgery or within 5 postoperative days, and/or hemodynamic instability requiring continued pharmacological support with ≥2 inotropic medications (epinephrine, milrinone dobutamine, dopamine) on postoperative day 1^31^
	Reoperation (valve related)	A valve reintervention after a previous valve intervention recorded in the dataset
	Postintervention valve dysfunction 1. Structural valve deterioration 2. Nonstructural valve dysfunction (including paravalvular insufficiency and device migration)	1. The term structural valve deterioration refers to changes intrinsic to the valve such as wear, fracture, poppet escape, calcification, leaflet tear, stent creep, and suture line disruption of components of a prosthetic valve; it also refers to new chordal rupture, leaflet disruption, or leaflet retraction of a repaired valve. 2. The term nonstructural dysfunction refers to problems (exclusive of thrombosis and infection) that do not directly involve valve components yet result in dysfunction of an operated valve, as diagnosed by reoperation, autopsy, or clinical investigation. Examples of nonstructural dysfunction include the following: entrapment by pannus, tissue, or suture paravalvular leak valvular leak; inappropriate sizing or positioning; residual leak or obstruction after valve implantation or repair; and clinically important intravascular hemolytic anemia. In addition, nonstructural dysfunction includes development of aortic or pulmonic regurgitation as a result of technical errors, dilatation of the sinotubular junction, or dilatation of the valve annulus after either valve replacement with stentless prostheses (e.g., pulmonary autograft, aortic allograft, and xenograft valves) or aortic valve–sparing operations if the cusps are seen to be normal at reoperation, autopsy, or clinical investigation. For percutaneous and transapical approaches to aortic valve replacement or conventional open aortic valve replacement, new onset of coronary ischemia from coronary ostial obstruction or paravalvular aortic regurgitation is considered nonstructural dysfunction.

Abbreviations: IABP, intra-aortic balloon pump; LVAD, left ventricular assist device; MI, myocardial infarction; VARC, Valve Academic Research Consortium.

Definitions of valve regurgitation were discussed separately by a focus group of experts and validated by the HVD working group (Table 4, Table 5, Table 6). Historical evaluation of regurgitation is classified in 3 grades (mild, moderate, and severe); different grading schemes are used in current research. Therefore, the Heart Valve Disease Working Group opted to move toward a uniform up-to-date evaluation of regurgitation in 4 grades (1–4) for aortic and mitral valve regurgitation.

**Table 4.** Grading the Severity of Aortic Valve Regurgitation.

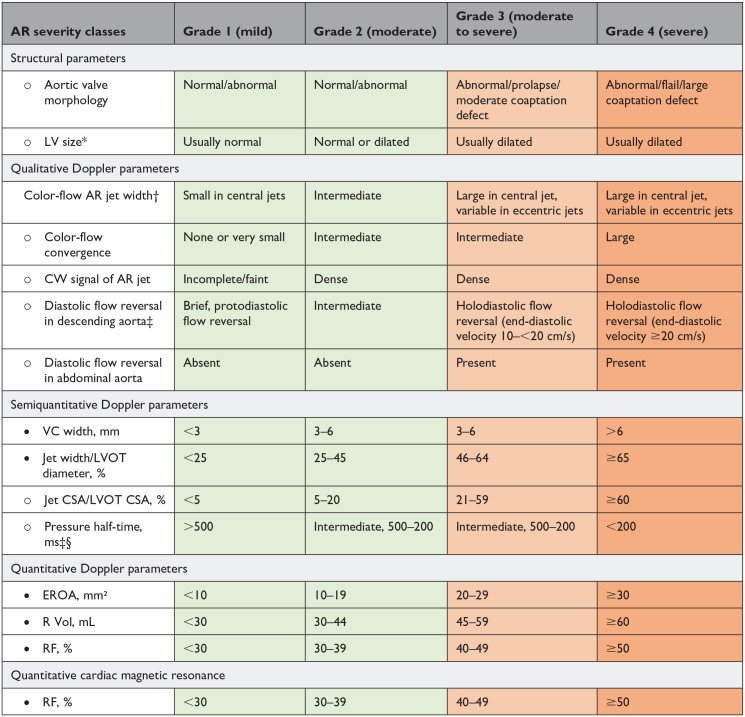

Abbreviations: AR, aortic regurgitation; CSA, cross-sectional area; CW, continuous wave; EROA, effective regurgitant orifice area; LV, left ventricular; LVOT, left ventricular outflow tract; R Vol, regurgitant volume; RF, regurgitant fraction; VC, vena contracta.

• Parameters that are more robust and should be given more weight to grade regurgitation severity by Doppler echocardiography.

○ Parameters that are less often applicable due to pitfalls in the feasibility/accuracy of the measurements or to the interaction with other factors.

* Unless there are other reasons, the LV size is usually normal in patients with mild AR. In acute severe AR, the LV size is often normal. Accepted cutoff values for nonsignificant LV enlargement are as follows: LV end-diastolic diameter <56 mm, LV end-diastolic volume <82 mL/m², LV end-systolic diameter <40 mm, and LV end-systolic volume <30 mL/m².

† At a Nyquist limit of 50 to 60 cm/s.

‡ These parameters are influenced by LV and aortic compliance. Hence, low transvalvular end-diastolic aorta to LV pressure gradient due to concomitant moderate/severe LV diastolic dysfunction may lead to false-positive results. The high dependency of aortic flow reversal on aortic compliance considerably limits the utility of this parameter in the elderly population. These parameters are also influenced by chronotropy.

§ Pressure half-time is shortened with increasing LV diastolic pressure and vasodilator therapy and in patients with a dilated compliant aorta or lengthened chronic AR.

**Table 5.** Grading the Severity of Mitral Regurgitation.

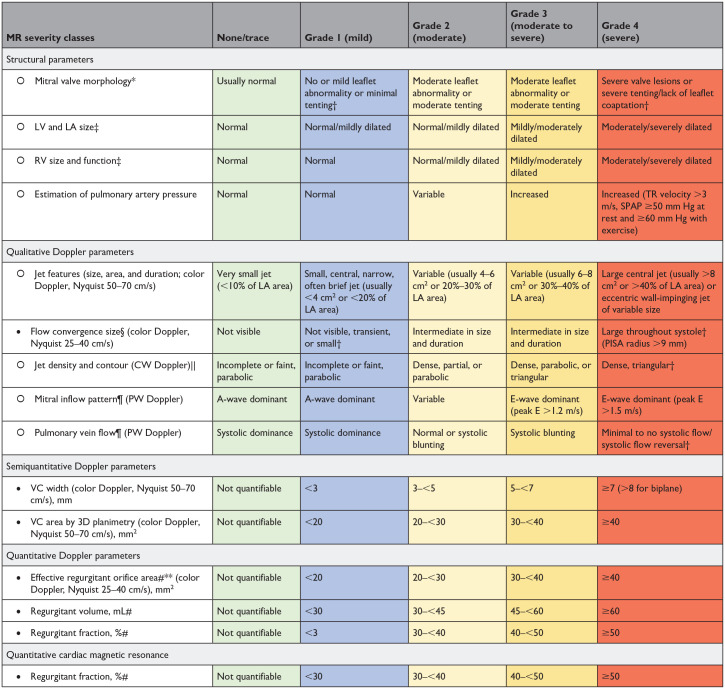

Abbreviations: CW, continuous wave; LA, left atrial; LV, left ventricular; MR, mitral regurgitation; PISA, proximal isovelocity surface area; PW, pulsed wave; RV, right ventricular; SPAP, systolic pulmonary arterial pressure; 3D, 3-dimensional; TR, tricuspid regurgitation; VC, vena contracta.

• Parameters that are more robust and should be given more weight to grade regurgitation severity by Doppler echocardiography.

○ Parameters that are less often applicable due to pitfalls in the feasibility/accuracy of the measurements or to the interaction with other factors.

* Primary MR: leaflet abnormalities include leaflet thickening, calcification, prolapse, flail, retraction, and perforation; secondary MR: mitral valve tenting, leaflet tethering, and lack of leaflet coaptation.

† Considered to be specific for their MR grade.

‡ Dilation of LV, LA, and RV may not be present despite moderate/severe MR in cases of acute MR. These structural parameters and criteria pertain mostly to patients with primary MR. Patients with secondary MR often have dilated LV regardless of the severity of MR.

§ Flow convergence is usually considered small with a PISA radius ≤3 mm and large with a radius ≥10 mm at a Nyquist limit of 25 to 40 cm/s.

|| Care must be taken to avoid overgaining or incomplete spectral traces (i.e., when the jet moves in and out of the Doppler beam).

¶ Mitral inflow pattern and pulmonary vein flow reversal may be influenced by LV systolic and diastolic function, LA size and pressure, atrial arrhythmias, and the presence of mitral inflow obstruction. However, holosystolic flow reversal is specific for severe MR.

# The regurgitant fraction is calculated by dividing the regurgitant volume by the total LV stroke volume measured by 3D echocardiography (total stroke volume=LV end-diastolic volume−LV end-systolic volume) or by Doppler (total stroke volume=regurgitant volume+LV forward stroke volume measured in LV outflow tract by pulsed-wave Doppler). A moderate regurgitant volume or effective regurgitant orifice area may correspond to a large regurgitant fraction and thus to a moderate to severe or severe MR in patients with depressed LV systolic function and low forward stroke volume (such as is often the case in patients with secondary MR). Hence, more weight should be given to the regurgitant fraction rather than other parameters to grade MR severity.

** By PISA or volumetric method.

**Table 6.** Grading Tricuspid Valve Regurgitation.

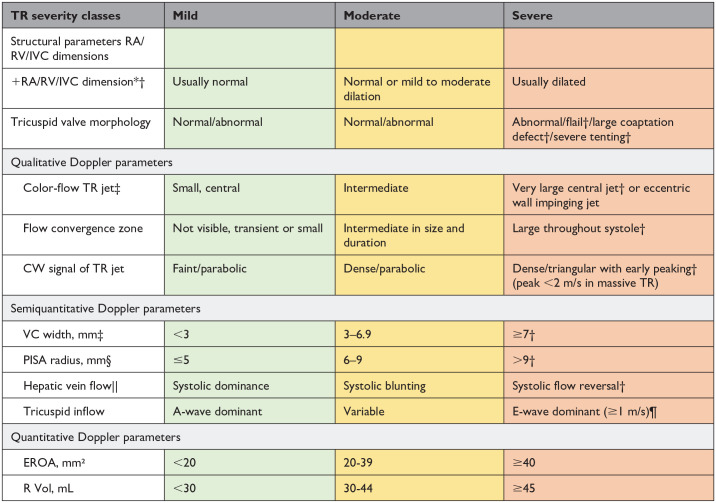

Abbreviations: CW, continuous-wave; EROA, effective regurgitant orifice area; IVC, inferior vena cava; PISA, proximal isovelocity surface area; R Vol, regurgitant volume; RA, right atrial; RV, right ventricular; TR, tricuspid regurgitation; VC, vena contracta.

An IVC diameter <2.1 cm is considered normal. The IVC is dilated when the diameter is >2.5 cm.

* Unless there are other reasons, the RA and RV sizes and IVC are usually normal in patients with mild TR. An end-systolic RV eccentricity index >2 is in favor of severe TR. In acute severe TR, the RV size is often normal. In chronic severe TR, the RV is classically dilated. Accepted cutoff values for nonsignificant right-sided chambers enlargement (measurements obtained from the apical 4-chamber view) are as follows: mid RV dimension ≤33 mm, RV end-diastolic area ≤28 cm², RV end-systolic area ≤16 cm², RV fractional area change >32%, and maximal 2-dimensional RA volume ≤33 mL/m².

† Specific signs for severe TR.

‡ At a Nyquist limit of 50 to 60 cm/s.

§ Baseline Nyquist limit shift of 28 cm/s.

|| Unless there are other reasons for systolic blunting (atrial fibrillation, elevated RA pressure).

¶ In the absence of other causes of elevated RA pressure.

### Outcome Definitions

In several HVD registries, trials, and methodology articles, definitions of the included outcomes differed. To increase granularity, the project group collected all definitions of the included outcomes, and the HVD working group voted on the most appropriate definition for each of them. This was done considering that this is a patient-centered set and that the most appropriate definition should affect patients’ lives and not be subclinical. Most of the definitions selected were based on previous VARC publications^8–10^ and the Akins et al.^23^ reporting guidelines to increase integration of existing databases and registries with the proposed dataset. In cases of postprocedural myocardial infarction, the HVD working group selected the Fourth Universal Definition of Myocardial Infarction.^24^ For a bleeding event, the HVD working group deviated from the VARC definition by excluding the hemoglobin drop because this may be caused by hemodilution during surgery and hemoglobin drop alone may have no effect on patients’ postoperative courses, whereas transfusion may have a profound effect.^11,25^ Definitions of all outcomes are presented in Table 2, Table 3, and in the Supplemental Reference Guide.

### Patient-Reported Outcome Measures

A review of PROMs used to provide health-related quality of life assessment in the setting of HVD revealed that 60 PROMs have previously been used in research settings, most of which have not been validated in the HVD population. Two PROMs were selected to monitor health-related quality of life, mental state, physical fitness, symptoms, and impact of HVD on daily life: the EQ-5D-5L questionnaire (EuroQol Group) and the Heart Valve Disease Impact on Daily Life Questionnaire.

The EQ-5D-5L questionnaire was selected to monitor quality of life and mental state.^26^ The questionnaire comprises 5 dimensions: mobility, self-care, usual activities, pain/discomfort, and anxiety/depression. Each dimension has 5 levels: no problems, slight problems, moderate problems, severe problems, and extreme problems. The patient is asked to indicate their health state by ticking the box next to the most appropriate statement in each of the 5 dimensions. This decision results in a 1-digit number that expresses the level selected for that dimension. The digits for the 5 dimensions can be combined into a 5-digit number that describes the patient’s health state. This specific questionnaire was specifically not validated in the HVD population but was validated in patients with myocardial infarction.^32^ The HVD working group selected this tool because it is widely used and easy to administer to patients.

To measure the impact of the HVD on patient’s daily life, the HVD working group selected the Heart Valve Disease Impact on Daily Life Questionnaire, which was validated in patients with HVD, and has good validity and reliability.^27,33,34^ The questionnaire consists of 14 items; its concept is that the impact is the product of the perceived consequence of the disease (part A) and the assessment of the consequence (part B), measured on a Likert scale 1 to 5 for both parts. The total score can vary between 14 and 350. A high score means that the patient perceives negative consequences of the disease in their life and these consequences are, in fact, interpreted as negative. A low score means that the patient does not perceive the negative consequences of the disease and its treatment, and if they do occur, the patient does not feel that they are too limiting.^34^ This questionnaire has additionally been validated in patients with heart failure and coronary artery disease.^35,36^

For the measure of symptoms, the New York Heart Association classification was selected for dyspnea, and the Canadian Cardiovascular Society grade for angina pectoris was selected; both are 1-question gradations of symptom severity.^28,29^

### Case-Mix Adjustment

**Fig. 2.**
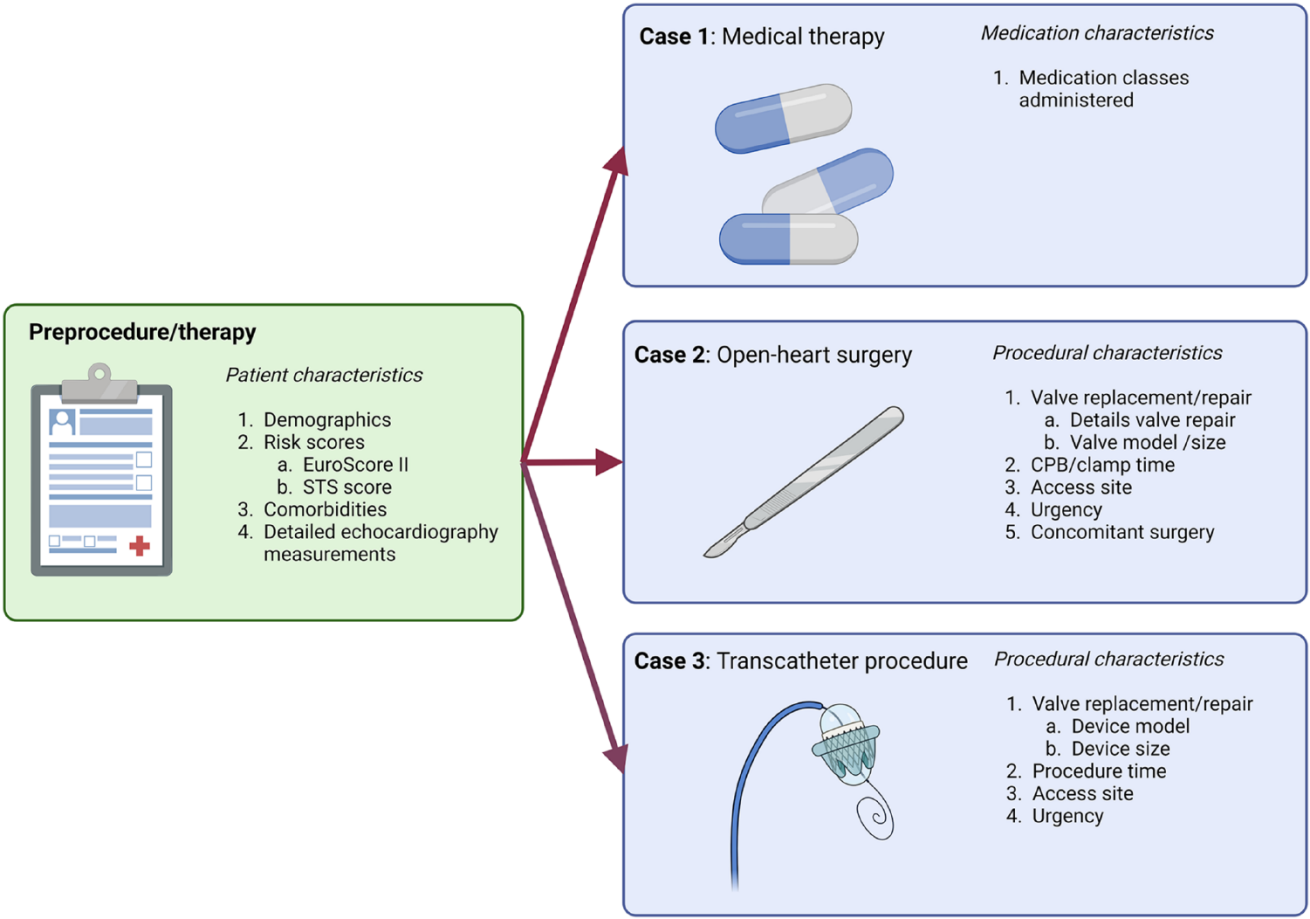
Simplified case mix of patients illustrated by 3 cases of patients undergoing surgical/medical/transcatheter treatment. CPB, cardiopulmonary bypass time; EuroSCORE, European System for Cardiac Operative Risk Evaluation; STS, The Society of Thoracic Surgeons.

Case-mix variables were categorized as demographic variables; echocardiographic variables; heart catheterization variables; and specific details on aortic, mitral, and tricuspid valves and their specific interventions. To allow meaningful comparison among patients undergoing different treatment strategies, 171 case-mix variables were selected. In a post hoc addition, 1 case-mix variable was added (dissection at surgery), and 2 case-mix variables on aortic dimensions were allowed to be measured repeatedly. Not all case-mix variables may be applicable for an individual patient because treatment variables are not applicable if a patient does not undergo invasive treatment, and, for example, specific mitral or tricuspid variables are not applicable for patients with isolated aortic valve disease. Furthermore, the treatment-related case-mix variables have a hierarchical structure (e.g., case-mix variables for percutaneous treatment are not applicable for patients undergoing surgical treatment and vice versa). All case-mix variables are presented in the Supplemental Reference Guide, and the simplified hierarchical structure of the case mix is illustrated by 3 cases in Figure 2. The full hierarchical structure of treatment related case mix is displayed in Supplemental Figure 3. The HVD working group chose to include specific valve repair devices and valve models implanted surgically or by transcatheter to allow meaningful comparison between treatment modalities. This implies that the Set should be updated as novel devices become available. The HVD working group recommends updating the Set every 5 years.

### Time Points

**Fig. 3.**
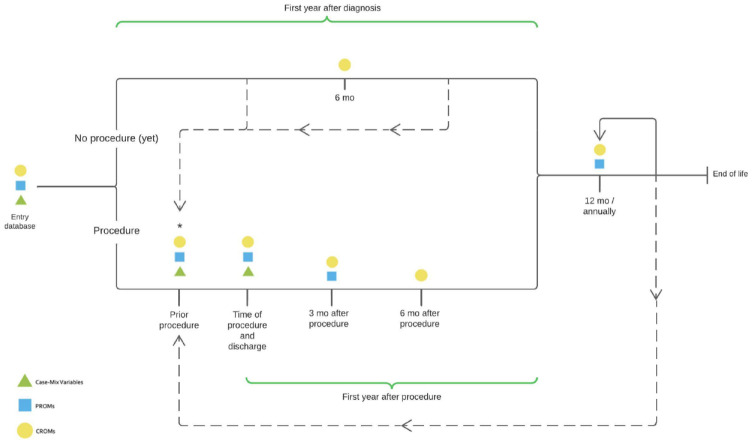
Proposed timelines of collecting outcome measures and case mix. *If <3 months elapsed between database entry and the procedure, the PROMs do not have to be captured. CROM, clinic-reported outcome measure; PROM, patient-reported outcome measure.

The HVD working group recommends collecting case-mix variables, clinical outcome measures, and PROMs at the index event, which is defined as when the patient enters the database. Ideally, the index event is when the patient is diagnosed with HVD, so disease progression and its impact on patients’ lives can be monitored before a procedure, if any. Tracking clinic-reported outcome measures at 6 months within the first year and annually thereafter and PROMs annually is recommended (Fig. 3). In cases of an undiagnosed HVD, patients’ data, including case mix, clinic-reported outcome measures, and PROMs, can be collected at the time of and after the valve procedure (Fig. 3). After the procedure, the recommendation is to track clinic-reported outcome measures at 3, 6, and 12 months and annually thereafter. The PROMs are tracked at 3 months after procedure and annually thereafter (Fig. 3).

## Discussion

**Fig. 4.**
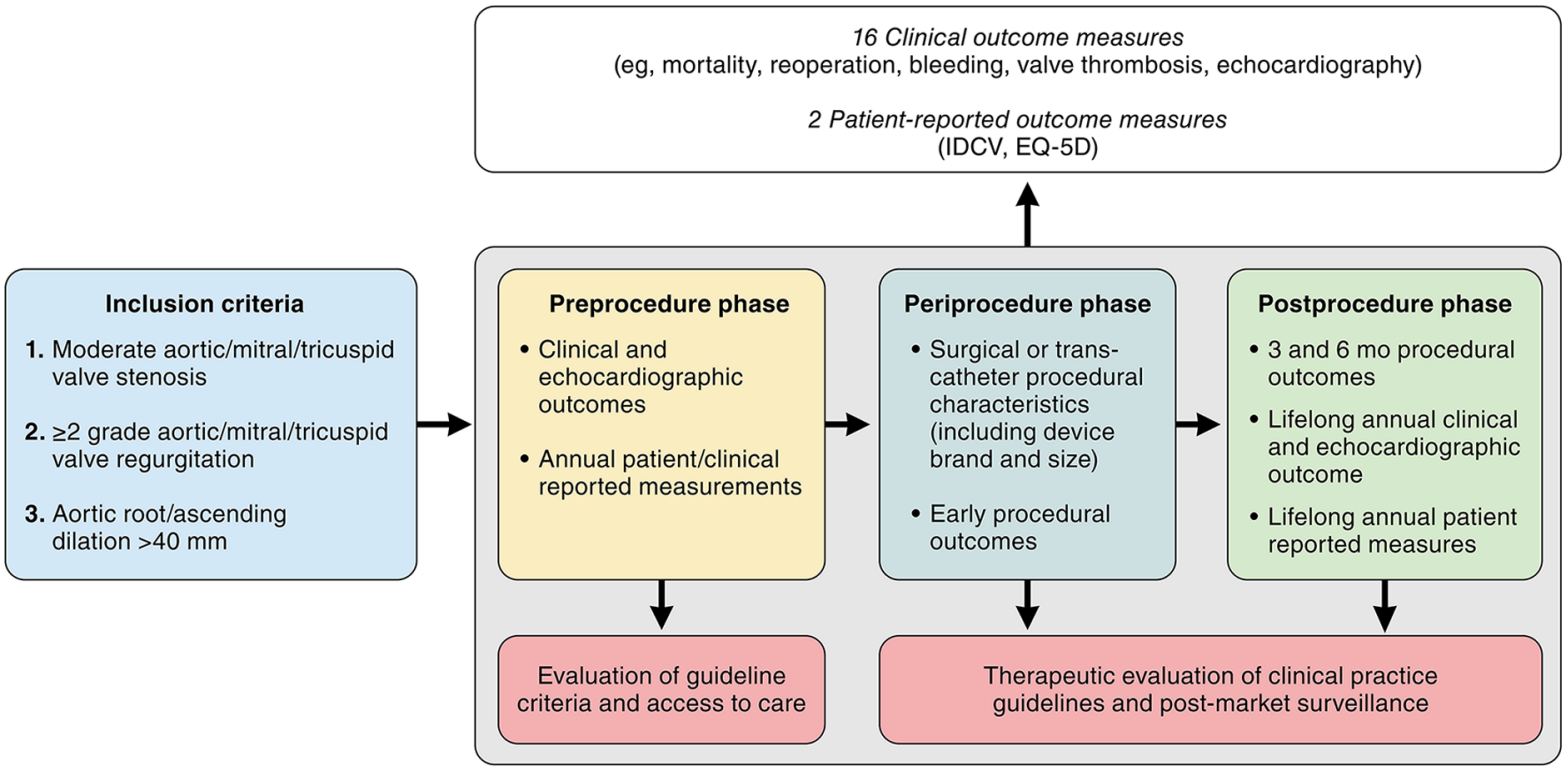
Heart valve disease International Consortium for Health Outcomes Measurement standard set allowing outcome measurement in preprocedural, periprocedural, and postprocedural phases of patients’ lives. IDCV, Heart Valve Disease Impact on Daily Life Questionnaire.

The ICHOM multisociety HVD working group reached consensus on a standardized set of outcomes, treatment, and adjustment variables to be tracked worldwide in patients with HVD. The proposed Set includes 16 (clinical) outcome measures, including 2 PROMs and 171 case-mix variables (Fig. 4). To move from current device-/procedure-/treatment-centered evaluation to patient-centered and disease-related outcomes, the Set was designed to be flexible and able to follow the whole journey of patients with HVD through the health care system, regardless of whether the treatment strategy is surgical or transcatheter. It was our aim to develop a Set that facilitates global standardization of outcomes in patients with HVD. This will allow comparison of the burden, management, and outcomes of HVD care across international borders, thereby contributing to improved patient selection, risk-adjustment models, and eventually higher quality of care for patients with HVD worldwide. The development of this Set was a multisociety and international effort including 12 societies and 12 nations on 4 continents. For the first time, all major surgical and cardiological societies, particularly those responsible for current clinical practice guidelines for the diagnosis and treatment of HVD, worked together to develop the first standardized Set for HVD that may be used as a complementary evaluation tool of future clinical practice guidelines. The HVD working group included not only experts in the field of heart disease but also patient representatives. Patients’ opinions and viewpoints have been invaluable for the development of this Set and allowed the focus to be on both clinical outcomes and outcomes that matter most to patients.

This set was built on prior work and previous efforts; both well-designed registries and clinical practice guidelines for reporting outcomes after valve interventions formed the basis of this Set.^8–10,17,18,21–23^ Overall, this Set combines all previous efforts, including the latest patient-centered definitions, and moves beyond short-term outcomes after valve intervention.^18,22^ Furthermore, this Set relies heavily on PROMs. Whereas several PROMs have been used previously, the HVD working group selected the EQ-5D-5L for quality of life and mental state. In contrast to VARC-3 guidelines for reporting after valve interventions, the HVD working group selected the Heart Valve Disease Impact on Daily Life Questionnaire instead of the recommended Minnesota Living With Heart Failure Questionnaire or the Kansas City Cardiomyopathy Questionnaire.^8^ This decision was also based on patients’ viewpoints; the Heart Valve Disease Impact on Daily Life Questionnaire allows patients to indicate whether a specific consequence of disease applies to them and whether they are bothered by this consequence. However, the Heart Valve Disease Impact on Daily Life Questionnaire does not have specific cutoff points to indicate good or bad outcomes, and analyzing the continuous data outcomes is recommended. Outcome data that are not discrete end points but rather “snapshots in time” such as valve dysfunction, PROMs, and symptoms are followed up longitudinally in this Set, allowing comprehensive models of repeated measures, making inference of imbalanced data possible.^37^

The aortic valve and aortic root/ascending aorta are closely intertwined, both surgically and anatomically. Thus, a significant number of patients will undergo aortic valve surgery with less than grade II regurgitation. To capture preoperative information in these patients, they can be included with root dilatation >40 mm, and in a post hoc adjustment, 1 case-mix variable was added to the set, and 2 case-mix variables for aortic dimensions were allowed to be measured repeatedly. However, the HVD working group acknowledges that the literature searches were not focused on identifying aneurysm-specific clinical outcome measures or PROMs. This adjustment should be viewed as a preliminary step toward the creation of a dedicated (aortic) aneurysm set, which is greatly needed.

Because the Set is endorsed by major societies, next steps include implementation of the Set in HVD clinical practice guidelines and societal databases. The HVD working group recognizes that this is not a straightforward task, and many challenges lie ahead. These include (1) ensuring local, regional, or national agreement to use the Set; (2) linking with existing registries; (3) building a user-friendly interface to enter data; (4) building a patient-friendly platform (e.g., application [app]) for PROMs collection; and (5) budgeting for the costs of numbers 3 and 5. As previous ICHOM set implementation efforts have shown,^38^ it seems essential that leadership support is present and an adequate health information system for collecting the data is developed. Although our intention is to eventually obtain a global evaluation of outcomes of patients who have HVD, a stepwise strategy is applied by initiating a pilot study in a small number of hospitals and performing a gap analysis to investigate which data are already collected and what infrastructure is most appropriate for adequate data collection.

A true global evaluation of patients’ outcomes measures will be possible only with deep commitment of health authorities, regulators, and payers, moving away from delegating the evaluation to industry like in the European Union Medical Device Regulation, with the inherent conflict of interest. Integration of ICHOM standards within the codification of health payment systems would allow exhaustive implementation, early warning for defective devices, and the necessary independent evaluation tools of patient’s outcomes measures. To date, ICHOM is collaborating with >650 organizations, including hospitals, universities, private insurance companies, and public payers (e.g., National Health Service). On a larger political scale, the Organisation for Economic Co-operation and Development and ICHOM signed a Letter of Intent in January 2017 to collaborate on the collection, analysis, and publishing of PROMs for international comparison (Patients-Reported Indications Surveys program).

## Conclusions

Through a unique collaborative effort among patients and cardiology and cardiothoracic surgery societies, including patient representatives, a standard Set for HVD was developed. This dataset focuses on outcome measurement regardless of treatment, moving from device- to patient-centered outcomes. Implementation of this Set will facilitate global standardization of outcome measurement and allow meaningful comparison among health care systems, evaluation of clinical practice guidelines, and eventual improvement of patient care worldwide for those who experience HVD.

## Supplemental Material

sj-pdf-1-inv-10.1177_15569845241269309 – Supplemental material for The First International Consortium for Health Outcomes Measurement (ICHOM) Standard Dataset for Reporting Outcomes in Heart Valve Disease: Moving From Device- to Patient-Centered Outcomes Supplemental material, sj-pdf-1-inv-10.1177_15569845241269309 for The First International Consortium for Health Outcomes Measurement (ICHOM) Standard Dataset for Reporting Outcomes in Heart Valve Disease: Moving From Device- to Patient-Centered Outcomes by Emmanuel Lansac, Kevin M. Veen, Andria Joseph, Paula Blancarte Jaber, Frieda Sossi, Zofia Das-Gupta, Suleman Aktaa, J. Rafael Sádaba, Vinod H. Thourani, Gry Dahle, Wilson Y. Szeto, Faisal Bakaeen, Elena Aikawa, Frederick J. Schoen, Evaldas Girdauskas, Aubrey Almeida, Andreas Zuckermann, Bart Meuris, John Stott, Jolanda Kluin, Ruchika Meel, Wil Woan, Daniel Colgan, Husam Balkhy, Molly Szerlip, Ourania Preventza, Pinak Shah, Vera H. Rigolin, Silvana Medica, Philip Holmes, Marta Sitges, Philippe Pibarot, Erwan Donal, Rebecca T. Hahn and Johanna J.M. Takkenberg in Innovations

sj-pdf-2-inv-10.1177_15569845241269309 – Supplemental material for The First International Consortium for Health Outcomes Measurement (ICHOM) Standard Dataset for Reporting Outcomes in Heart Valve Disease: Moving From Device- to Patient-Centered Outcomes Supplemental material, sj-pdf-2-inv-10.1177_15569845241269309 for The First International Consortium for Health Outcomes Measurement (ICHOM) Standard Dataset for Reporting Outcomes in Heart Valve Disease: Moving From Device- to Patient-Centered Outcomes by Emmanuel Lansac, Kevin M. Veen, Andria Joseph, Paula Blancarte Jaber, Frieda Sossi, Zofia Das-Gupta, Suleman Aktaa, J. Rafael Sádaba, Vinod H. Thourani, Gry Dahle, Wilson Y. Szeto, Faisal Bakaeen, Elena Aikawa, Frederick J. Schoen, Evaldas Girdauskas, Aubrey Almeida, Andreas Zuckermann, Bart Meuris, John Stott, Jolanda Kluin, Ruchika Meel, Wil Woan, Daniel Colgan, Husam Balkhy, Molly Szerlip, Ourania Preventza, Pinak Shah, Vera H. Rigolin, Silvana Medica, Philip Holmes, Marta Sitges, Philippe Pibarot, Erwan Donal, Rebecca T. Hahn and Johanna J.M. Takkenberg in Innovations

sj-pdf-3-inv-10.1177_15569845241269309 – Supplemental material for The First International Consortium for Health Outcomes Measurement (ICHOM) Standard Dataset for Reporting Outcomes in Heart Valve Disease: Moving From Device- to Patient-Centered Outcomes Supplemental material, sj-pdf-3-inv-10.1177_15569845241269309 for The First International Consortium for Health Outcomes Measurement (ICHOM) Standard Dataset for Reporting Outcomes in Heart Valve Disease: Moving From Device- to Patient-Centered Outcomes by Emmanuel Lansac, Kevin M. Veen, Andria Joseph, Paula Blancarte Jaber, Frieda Sossi, Zofia Das-Gupta, Suleman Aktaa, J. Rafael Sádaba, Vinod H. Thourani, Gry Dahle, Wilson Y. Szeto, Faisal Bakaeen, Elena Aikawa, Frederick J. Schoen, Evaldas Girdauskas, Aubrey Almeida, Andreas Zuckermann, Bart Meuris, John Stott, Jolanda Kluin, Ruchika Meel, Wil Woan, Daniel Colgan, Husam Balkhy, Molly Szerlip, Ourania Preventza, Pinak Shah, Vera H. Rigolin, Silvana Medica, Philip Holmes, Marta Sitges, Philippe Pibarot, Erwan Donal, Rebecca T. Hahn and Johanna J.M. Takkenberg in Innovations
