# Supplementary material for: The First International Consortium for Health Outcomes Measurement (ICHOM) Standard Dataset for Reporting Outcomes in Heart Valve Disease: Moving From Device- to Patient-Centered Outcomes
Source: Innovations (Phila). 2025 Feb 19;20(2):133–47. doi: 10.1177/15569845241269309 (PMC12089684; doi:10.1177/15569845241269309)
Supplement: sj-pdf-1-inv-10.1177_15569845241269309 – Supplemental material for The First International Consortium for Health Outcomes Measurement (ICHOM) Standard Dataset for Reporting Outcomes in Heart Valve Disease: Moving From Device- to Patient-Centered Outcomes [file sj-pdf-1-inv-10.1177_15569845241269309.pdf]

ICHOM International Consortium for Health Outcomes Measurement

HEART VALVE DISEASE DATA COLLECTION REFERENCE GUIDE

Version 5.0.2
Revised: December 2023

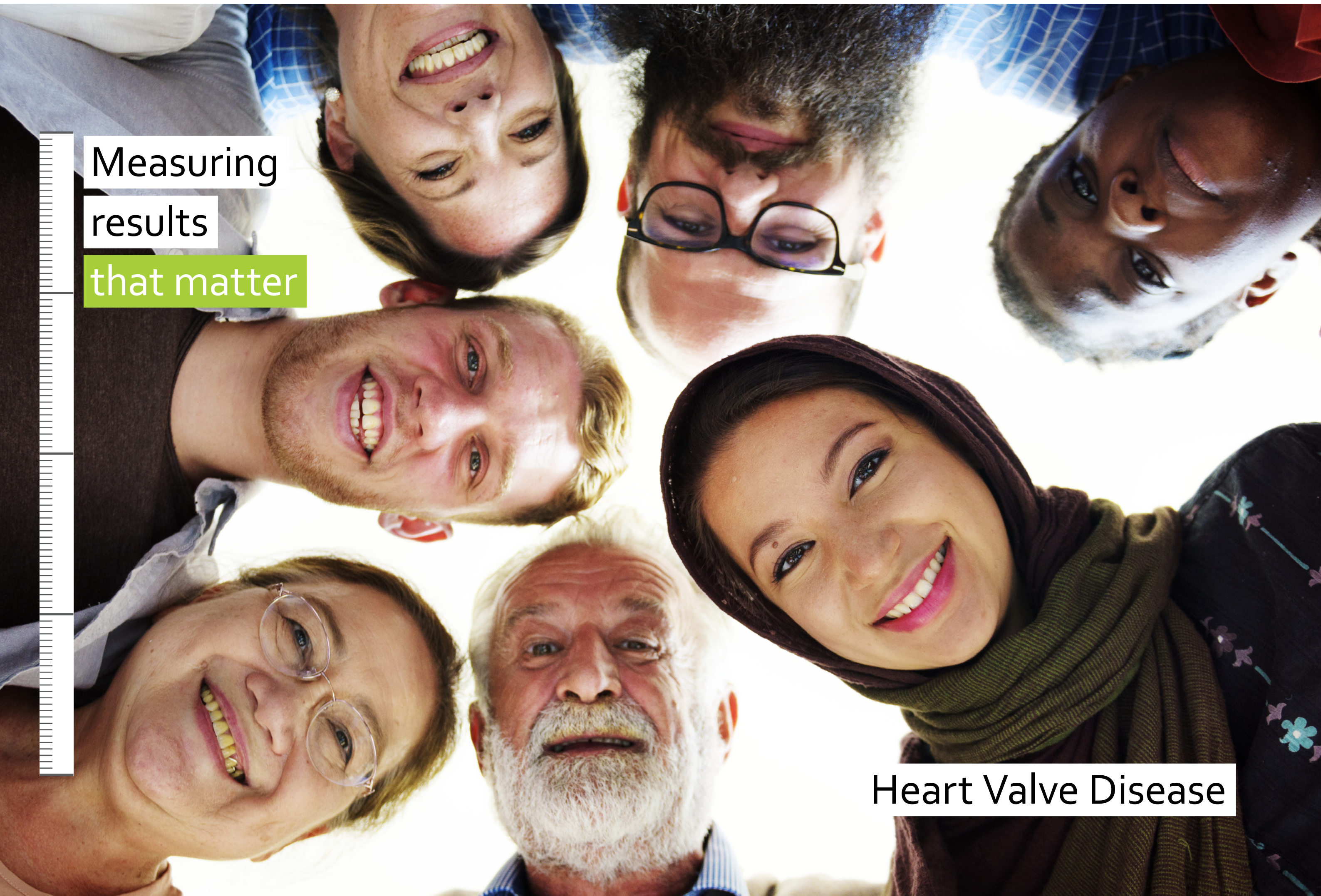

ICHOM HEART VALVE DISEASE

We are thrilled that you are interested in measuring outcomes for your Heart Valve Disease patients according to ICHOM standards. It is our hope that this Reference Guide will facilitate the process of implementing our Set of Patient-Centered Outcome Measures and ensure collection of comparable data for global benchmarking and learning.

© 2023 ICHOM. All rights reserved. When using this set of outcomes, or quoting therefrom, in any way, we solely require that you always make a reference to ICHOM as the source so that this organization can continue its work to define more standard outcome sets.

Introducing ICHOM and the Reference Guide

ICHOM brings together patient representatives, clinician leaders, and registry leaders from all over the world to develop Sets of Patient-Centered Outcome Measures, comprehensive yet parsimonious sets of outcomes and case-mix variables we recommend all providers track.

Each Set of Patient-Centered Outcome Measures focuses on patient-centered results and provides an internationally-agreed upon method for measuring each of these outcomes. We do this because we believe that standardized outcomes measurement will open up new possibilities to compare performance globally, allow clinicians to learn from each other, and rapidly improve the care we provide our patients.

Our Sets include initial conditions and risk factors to enable meaningful case-mix adjustment globally, ensuring that comparisons of outcomes will take into account the differences in patient populations across not just providers, but also countries and regions. A comprehensive data dictionary, as well as scoring guides for patient-reported outcomes, is included in the appendix.

Our aim is to make Sets of Patient-Centered Outcome Measures freely accessible to healthcare institutions worldwide to begin measuring, and ultimately benchmark the outcomes they achieve. In order to have a guide from which we can benchmark outcomes, we require feedback from initial implementation efforts. As such, this Reference Guide may undergo revisions on a regular basis. If you have any suggestions or would like to provide feedback, please contact info@ichom.org.

ICHOM Cardiometabolic Family of Sets - Updates

As ICHOM strives to keep our Sets up to date with clinical advancements, implementation requirements, and relevant to patient interests, we have begun an initiative to review and update all our Sets routinely every three years.

The Cardiometabolic Family of Sets represents the first group of Sets to be revised and updated as a group simultaneously. This marks an important milestone in ICHOM's journey to promote value-based healthcare from an evidence-based and patient-centered perspective. For this process, we have worked with a Steering Committee, a group of experts from the original Working Groups involved in the development of these Sets, implementers from around the world, and patient representatives, to make necessary changes to the Sets in order to ensure that they are clinically up to date and harmonized in line with ICHOM standards.

These changes include:

- Set specific updates (specific updates to Sets individually, based on feedback and in order to keep in line with clinical advancements)
- Cross-Set updates (updates made across all Cardiometabolic Sets, with the aim to harmonize standardized variables throughout different Sets in order to facilitate simultaneous implementation)

A full list of changes specific to the Heart Valve Disease Set can be seen in the Appendix.

Working Group Members for Heart Valve Disease

The following individuals dedicated both time and expertise to develop the ICHOM Sets of Patient-Centered Outcome Measures for Heart Valve Disease in partnership with ICHOM, under the leadership of Prof. Hanneke Takkenberg from the Erasmus University Medical Center, and Dr. Emmanuel Lansac from the Hôpital Pitié Salpétrière, Paris, as ICHOM Chairs; the project was supported by Dr Kevin Veen from the Erasmus University Medical Center as Reasearch Fellow, Dr. Zofia Das-Gupta as Director of Outcomes Research, Andria Joseph as Project Leader, and Dr. Paula Blancarte Jaber and Frieda Sossi as Research Associates.

Australia
Aubrey Almeida
Philip Holmes

Austria
Andreas Zuckermann

Belgium
Bart Meuris

Canada
John Stott

Germany
Evaldas Girdauskas

Italy
Silvana Medica

The Netherlands
Jolanda Kluin

Norway
Gry Dahle

Spain
Rafael Sádaba

South Africa
Ruchika Meel

United Kingdom
Suleman Aktaa
Wil Woan
Daniel Colgan

United States
Elena Aikawa
Faisal G. Bakaeen
Frederick Schoen
Husam Balkhy
Ourania Preventza
Pinak Shah
Vera H. Rigolin
Wilson Y. Szeto
Hani Jneid
Martin Leon
Molly Szerlip

Steering Committee Members who contributed to the update of the Cardiometabolic Family of Sets

The following individuals dedicated both time and expertise to update the ICHOM CardioMetabolic Family of Sets. The work was supported by ICHOM Project Managers Paula Blancarte Jaber and Spencer Connell, ICHOM Director of Outcomes Research Zofia Das-Gupta, and ICHOM Research Associate Isabel Miller.

Elena Arbelo
Menno Huisman
Andreas Bollman
Benjamin Steinberg
John Beltrame
Tom Lumbers
Cristina García Ulloa
Andrew Pumerantz
Sergio Hernández
Søren Skovlund
Mark Peyrot
Magdalena Walbaum
Erik (F.A.) Klok
Albertino Damasceno
Camila de Menezes Succi
Jana Nano
Kevin Veen
Cindy de Jong
Tim Benson

Supporting Organizations

This Set of Patient-Centered Outcome Measures is made possible only through the partnership with Heart Valve Society, the support of Edwards LifeSciences and Adetec Coeur, and in collaborations with the European Society of Cardiology, the Australian and New Zealand Society of Cardiac &Thoracic Surgeons, the Cardiovascular Research Foundation, the American Heart Association, the International Society for Applied Cardiovascular Biology, the American College of Cardiology, the South Africa Heart Association, the American Association for Thoracic Surgery, the Society of Thoracic Surgeons, the European Association for Cardio-Thoracic Surgery, and the International Society for Minimally Invasive Cardiothoracic Surgery.

Thank you.

DEVELOPED IN PARTNERSHIP WITH

Endorsed by:

ICHOM Sponsors

The Cardiometabolic Family Set Updates would not have been possible without the support of the following sponsor:

Conditions and Treatment Approaches Covered for Heart Valve Disease

For Heart Valve Disease, the following conditions and treatment approaches (or interventions) are covered by our Set of Patient-Centered Outcome Measures.

**Conditions**	Adults (≥18 years old) with heart valve disease
**Treatment Approaches**	Pharmacological | Transcatheter/Surgical Intervention | Communicate steps for management, self-care, and follow-up

Notes on the Heart Valve Disease Set

This Set represents the first ever multi-society taskforce, which involves Working Group members representing the multiple cardiology societies and associations presented above. We are currently in the process of seeking official and public endorsement from these societies, in order to expand our outreach in the cardiology field and become leaders in the area of patient-centered outcome measures for heart valve disease globally.

A number of the variables included in this dataset require specific clinical information, as the responses given can largely affect the outcome of a patient; notably, the manufacturer used for valve replacements may result in different outcomes among patients. Please see the additional attached documents for more information.

ICHOM Set for Heart Valve Disease

Case-Mix Variables

Patient Population	Measure	Timing	Reporting Source
**Demographic factors**			
All patients	Year of birth	Baseline; at time of procedure	Clinical
	Sex		Clinical
	Gender		Patient-reported
	Body height		Clinical
	Body weight		Clinical
	Anticoagulants		Clinical
	Extracardiac arteriopathy		Clinical
	Critical Preoperative State		Clinical
	Pulmonary hypertension		Clinical
	Dialysis		Clinical
	Recent Myocardial infarction		Clinical
	Prior cardiac procedure		Clinical
	Diabetes		Clinical
	Chronic Lung Disease		Clinical
	NYHA		Clinical
	Endocarditis		Clinical
	Creatinine		Clinical
	Poor mobility		Clinical
	Angina		Clinical
Patients undergoing intervention	Euroscore II	At time of procedure	Clinical
**Echocardiographic factors**			
All patients	Left ventricle dimensions	Index event; at time of procedure	Clinical
	Left ventricle function		
	Right ventricle function		
**Valve-related factors**			
All patients	Aortic valve details	Index event; at time of procedure	Clinical
	Mitral valve details		
	Tricuspid valve details		
**Treatment-related factors**			
All patients	Heart catheterization	Index event; at time of procedure	Clinical
	Baseline rhythm		
	Treatment		

Outcomes

Patient-reported outcomes			
All patients	All cause mortality	Annually	Clinical
	Quality of Life	Baseline; annually; at procedure; 3 months after procedure	Patient-reported
	Mental Health		
	Impact on Mental Health and Daily Activities		

Clinical outcomes			
All patients	Hospitalisation for heart failure	Annually	Clinical
	Valve dysfunction	At discharge, 3 months, and 6 months after intervention; then annually	
	Cardiac status	Annually (if 0)<br>6 months (if >0)	
	Rhythm	Prior to and post procedure, 3 and 6 months after discharge; then annually	
	Left ventricular ejection fraction		
	Bleeding event	Index event	
	Endocarditis	Annually	
	Valve Thrombosis	Annually	
Patients that underwent valve replacement	Structural Valve Deterioration	At discharge, 3 months, and 6 months after intervention; then annually	
	Non-structural valve deterioration		
All patients	Stroke	Annually	
	Thromboembolic Event (non-cerebral)		
All patients who have undergone a heart valve procedure	Re-intervention	At intervention	
All patients who have undergone a minimally invasive heart valve procedure.	Operative complications		

Follow-Up Timeline

The following timeline illustrates when Set variables should be collected from patients, clinicians, and administrative sources.

All patients with Heart Valve Disease, regardless of treatment

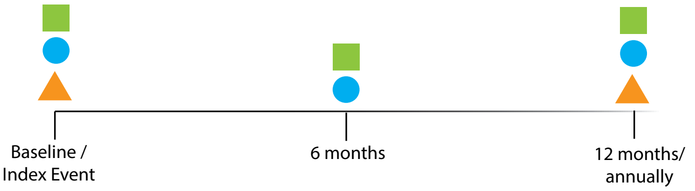

All patients with Heart Valve Disease who have undergone a procedure

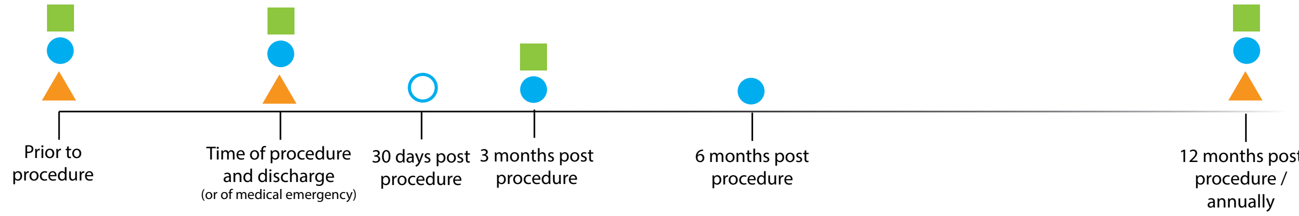

*Index event is considered as the time the patient is diagnosed with heart valve disease and/or enters the database.

The timeline is not reset in the case of hospitalization or medical emergency

The following questionnairs should be adminis-tered at the indicated time points:

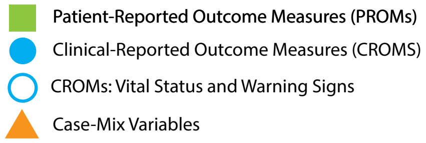

Collecting Patient-Reported Outcome Measures

Survey(s) Used	Licensing Information	Scoring Guide
EQ-5D-5L - EuroQol Group	Free to use for non-commercial purposes and in clinical practice without a license. Information available at: https://euroqol.org/eq-5d-instruments/eq-5d-5l-about/	A user guide for this questionnaire can be found here: https://euroqol.org/publications/user-guides/
Heart Valve Disease Impact on Daily Life (IDCV)	Free access.	Information on this questionnaire can be found through the following sources: https://pubmed.ncbi.nlm.nih.gov/17584347/ ; https://pubmed.ncbi.nlm.nih.gov/27508475/

The **IDCV** was developed as a screening measure for Activities for Daily Life . It is a **14 item self-report** instrument.
Created and validated in Heart Valve Disease Patients

- **5 point Likert scale**
- Minimum score of 1 and a maximum of 25 for each statement evaluated. The closer the score is to 1, the lower the impact felt by the subject, and the closer it is to 25, the greater the impact.

Time	9.9 - 10 minutes
Languages	-
Ages	Adults

The Growing ICHOM Community

There is a growing community of healthcare providers implementing the ICHOM Sets. To support your organization in implementing the set and the measurement of outcomes data, we have outlined a framework to guide the implementation and reporting of patient-centered outcomes. All Set materials can be downloaded for free from ICHOM Connect, for further information or to enquire about implementation support offered by ICHOM Partners, please contact us: info@ichom.org.

Implementation framework:
The framework below, outlines the structured process to guide the implementation of an ICHOM Set at your organization. Typically, an implementation project takes 9 months to complete.

Diagnostic and Service Scoping

- Assess IT and informatics infrastructure within pilot site
- Perform a gap analysis of what data is collected versus what isn't
- Assess and define scope for project
- Secure PROM licenses for Standard Set as required
- Determine and process map pilot implementation site

Preparation

- Recruit local Project Manager
- Secure additional IT/Information platforms to address data gaps
- Establish project team and governance structure
- Plan for launch with key stakeholders to achieve clinical buy-in
- Develop an implementation plan to guide the project

Roll Out

- Deploy IT solution
- Pilot data collection with part of dataset
- Assess Pilot period
- Refine Workflow and IT systems using PDSA cycles

Measurement

- Scale up to implement full dataset for all patients within scope
- Collect data on every patient, ensure data completeness and validity
- Troubleshoot full dataset issues & quality assure data through audit
- Begin to analyze full dataset and report to clinicians and patients

Change Management

Implementation Study:
We are keen to find out if you have implemented or are implementing our ICHOM Sets. Please fill in this survey: bit.ly/InitialImp or contact info@ichom.org for more information.

Translating the Set Tools:

PROMs within the ICHOM Sets are available in a number of languages. To check the availability of translations, we advise contacting the Tool authors directly to obtain and translate the PROM surveys into your desired language. To independently translate PROM surveys, if permitted by its license, we recommend following the 10 steps outlined below:*[1]

**Step 1**	Preparation	Initial work carried out before the translation work begins
**Step 2**	Forward Translation	Translation of the original language, also called source, version of the instrument into another language, often called the target language
**Step 3**	Reconciliation	Comparing and merging more than one forward translation into a single forward translation
**Step 4**	Back Translation	Translation of the new language version back into the original language
**Step 5**	Back Translation Review	Comparison of the back-translated versions of the instrument with the original to highlight and investigate discrepancies between the original and the reconciled translation, which is then revised in the process of resolving the issues
**Step 6**	Harmonization	Comparison of back translations of multiple language versions with each other and the original instrument to highlight discrepancies between the original and its derivative translations, as well as to achieve a consistent approach to translation problems
**Step 7**	Cognitive Debriefing	Testing the instrument on a small group of relevant patients or lay people in order to test alternative wording and to check understandability, interpretation, and cultural relevance of the translation
**Step 8**	Review of Cognitive Debriefing Results and Finalization	Comparison of the patients' or lay persons' interpretation of the translation with the original version to highlight and amend discrepancies
**Step 9**	Proofreading	Final review of the translation to highlight and correct any typographic, grammatical or other errors
**Step 10**	Final Report	Report written at the end of the process documenting the development of each translation

*These ten steps follow the ISPOR Principles of Good Practice: The Cross-Cultural Adaptation Process for Patient-Reported Outcomes Measures [1] Wild, D., Grove, A., Martin, M., Eremenco, S., McElroy, S., Verjee-Lorenz, A., et al. (2005). Principles of good practice for the translation and cultural adaptation process for patient-reported outcomes (PRO) measures: Report of the ISPOR task force for translation and cultural adaptation. Value in Health, 8(2), 94–104. doi:10.1111/j.1524-4733.2005.04054.

Appendix

Introduction to the Data Dictionary

This data dictionary is designed to help you measure the ICHOM Heart Valve Disease Set as consistently as possible to the Working Group recommendation. **For technical use an Excel version of this data dictionary is also available for download on ICHOM Connect. Excel data dictionary is the most up-to-date version and it is the recommended document to plan data collection.**

Please timestamp all variables. Some Set variables are collected at multiple timepoints, and we will ask you to submit these variables in a concatenated VARIABLEID_TIMESTAMP form for future analyses. For example, VARIABLEID_BASE (baseline); VARIABLEID_6MO (6 month follow-up); VARIABLEID_1YR (1 year follow-up), etc.

**Variable ID:**	N/A
**Variable:**	Patient ID
**Definition:**	Create a unique patient identifier (e.g. medical record number)
**Supporting Definition:**	This number will not be shared with ICHOM. In the case patient-level data is submitted to ICHOM for benchmarking or research purposes, a separate ICHOM Patient Identifier will be created and cross-linking between the ICHOM Patient Identifier and the medical record number will only be known at the treating institution
**Displayed Value**	None
**Inclusion Criteria:**	All patients
**Timing:**	On all forms
**Reporting Source:**	Administrative or clinical
**Type:**	Numerical
**Value Domain:**	None
**Response Options:**	According to institution

Demographic Factors

**Variable ID:**	YearOfBirth
**Variable:**	Year of birth
**Definition:**	Year of birth
**Supporting Definition:**	None
**Displayed Value**	In what year were you born?
**Inclusion Criteria:**	All patients
**Timing:**	Baseline; at time of procedure
**Reporting Source:**	Clinical
**Type:**	Date by YYYY
**Value Domain:**	Date
**Response Options:**	YYYY

**Variable ID:**	Sex
**Variable:**	Sex
**Definition:**	The patient's sex at birth
**Supporting Definition:**	For statistical purposes, the following category codes, labels and definitions are preferred:<br>CODE 1 Male: Persons who have male or predominantly masculine biological characteristics, or male sex assigned at birth.<br>CODE 2 Female: Persons who have female or predominantly feminine biological characteristics, or female sex assigned at birth.<br>CODE 3 Other: Persons who have mixed or non-binary biological characteristics (if known), or a non-binary sex assigned at birth<br>The value meaning of 'Other' has been assigned to Code 3 for this value domain, which replaces 'Intersex or indeterminate' for the superseded value domain Sex code N. Terms

such as 'indeterminate,' 'intersex', 'non-binary', and 'unspecified' are variously used to describe the 'Other' category of sex. The label 'Other' is used because a more descriptive term has not been widely agreed within the general community.
Sex refers to the chromosomal, gonadal and anatomical characteristics associated with biological sex. Where there is an inconsistency between anatomical and chromosomal characteristics, sex is based on anatomical characteristics.

Displayed Value Please indicate your sex at birth.
Inclusion Criteria: All patients
Timing: Baseline; at time of procedure
Reporting Source: Clinical
Type: Single answer
Value Domain: Code
Response Options: 1 = Male
2 = Female
3 = Other
999 = Undisclosed

Variable ID: Gender_CVD
Variable: Gender identity
Definition: The patient's gender identity
Supporting Definition: This measure should be recorded if appropriate and legal based on local standards in the particular geographic region, and should be self-reported by the patient. This is an optional question but ICHOM encourages that this information is collected. This data will help to support combating health disparities based on gender identity but all patient data regarding gender identity will be kept confidential. The patient's response will then be coded based on LOINC's standards. All patients may choose not to answer as well.
Displayed Value Do you think of yourself as ... ?
Inclusion Criteria: All patients
Timing: Baseline; at time of procedure
Reporting Source: Patient-reported
Type: Single answer
Value Domain: Code
Response Options: 1 = Boy/Man
2 = Girl/Woman
3 = Non-Binary
4 = Trans man/Transgender Man/FTM
5= Trans woman/Transgender woman/MTF
6 = None of these describe me
999 = Prefer not to answer

Variable ID: heightvalue
Variable: body height
Definition: the height of a person measured in the indicated units
Supporting Definition: The measurement protocol described below are those recommended by the International Society for the Advancement of Kinanthropometry as described by Norton et al. (1996), and the World Health Organization (WHO Expert Committee 1995), which was adapted from Lohman et al. (1988).Measurement protocol:Height measurements can be based on recumbent length or standing height. In general, length measurements are recommended for children under 2 years of age and height measurements for others.The measurement of height requires a vertical metric rule, a horizontal headboard, and a non-compressible flat even surface on which the subject stands. The equipment may be fixed or portable, and should be described and reported.The graduations on the metric rule should be at 0.1 cm intervals, and the metric rule should have the capacity to measure up to at least 210 cm.Measurement intervals and labels should be clearly readable under all conditions of use of the instrument.Apparatus that allows height to be measured while the subject stands on a platform scale is not recommended.Adults and children who can stand:The subject should be measured without shoes (i.e. is barefoot or wears thin socks) and wears little clothing so that the positioning of the body can be seen. Anything that may affect or interfere with the measurement should be noted on the data collection form (e.g. hairstyles and accessories, or physical problems). The subject stands with weight distributed evenly on both feet, heels together, and the head positioned so that the line of vision is at right angles to the body. The correct position for the head is in the Frankfort horizontal plan (Norton et al. 1996). The arms hang freely by the sides. The head, back,

buttocks and heels are positioned vertically so that the buttocks and the heels are in contact with the vertical board. To obtain a consistent measure, the subject is asked to inhale deeply and stretch to their fullest height. The measurer applies gentle upward pressure through the mastoid processes to maintain a fully erect position when the measurement is taken. Ensure that the head remains positioned so that the line of vision is at right angles to the body, and the heels remain in contact with the base board.The movable headboard is brought onto the top of the head with sufficient pressure to compress the hair.The measurement is recorded to the nearest 0.1 cm. Take a repeat measurement. If the two measurements disagree by more than 0.5 cm, then take a third measurement. All raw measurements should be recorded on the data collection form. If practical, it is preferable to enter the raw data into the database as this enables intra-observer and, where relevant, inter-observer errors to be assessed. The subject's measured height is subsequently calculated as the mean of the two observations, or the mean of the two closest measurements if a third is taken, and recorded on the form. If only a mean value is entered into the database then the data collection forms should be retained.It may be necessary to round the mean value to the nearest 0.1 cm. If so, rounding should be to the nearest even digit to reduce systematic over reporting (Armitage & Berry 1994). For example, a mean value of 172.25 cm would be rounded to 172.2 cm, while a mean value of 172.35 cm would be rounded to 172.4 cm.Infants:For the measurement of supine length of children up to and including 2 years of age, two observers are required. One observer positions the head correctly while the other ensures the remaining position is correct and brings the measuring board in contact with the feet. The subject lies in a supine position on a recumbent length table or measuring board. The crown of the head must touch the stationary, vertical headboard. The subject's head is held with the line of vision aligned perpendicular to the plane of the measuring surface. The shoulders and buttocks must be flat against the table top, with the shoulders and hips aligned at right angles to the long axis of the body. The legs must be extended at the hips and knees and lie flat against the table top and the arms rest against the sides of the trunk. The measurer must ensure that the legs remain flat on the table and must shift the movable board against the heels. In infants care has to be taken to extend the legs gently. In some older children two observers may also be required.In general, length or height is measured and reported to the nearest 0.1 cm. For any child, the length measurement is approximately 0.5–1.5 cm greater than the height measurement. It is therefore recommended that when a length measurement is applied to a height-based reference for children over 24 months of age (or over 85 cm if age is not known), 1.0 cm be subtracted before the length measurement is compared with the reference. It is also recommended that as a matter of procedure and data recording accuracy, the date be recorded when the change is made from supine to standing height measure.Validation and quality control measures:All equipment, whether fixed or portable should be checked prior to each measurement session to ensure that both the headboard and floor (or footboard) are at 90 degrees to the vertical rule. With some types of portable anthropometer it is necessary to check the correct alignment of the headboard, during each measurement, by means of a spirit level. Within- and, if relevant, between-observer variability should be reported. They can be assessed by the same (within-) or different (between-) observers repeating the measurement of height, on the same subjects, under standard conditions after a short time interval. The standard deviation of replicate measurements (technical error of measurement (Pederson & Gore 1996)) between observers should not exceed 5 mm and be less than 5 mm within observers.Extreme values at the lower and upper end of the distribution of measured height should be checked both during data collection and after data entry. Individuals should not be excluded on the basis of true biological difference. Last digit preference, and preference or avoidance of certain values, should be analysed in the total sample and (if relevant) by observer, survey site and over time if the survey period is long.

Displayed Value please indicate your body height
Inclusion Criteria: All patients
Timing: Baseline
Reporting Source: Clinical
Type: Numerical
Value Domain: quantity
Response Options: Numerical value of height

Variable ID: heightunit
Variable: body height units

Definition: units of body height
Supporting Definition: None
Displayed Value Please indicate what units of measurement (centimeters or inches) that you recorded your height in.
Inclusion Criteria: All patients
Timing: Baseline; at time of procedure
Reporting Source: Clinical
Type: Singe answer
Value Domain: Code
Response Options: 1 = centimeters
2 = inches

Variable ID: weightvalue
Variable: body weight
Definition: the body weight of a person measured in the indicated units
Supporting Definition: the collection of anthropometric measurements particularly in those who are overweight or obese or who are concerned about their weight should be performed with great sensitivity and without drawing attention to an individuals weight
Displayed Value please indicate your body weight
Inclusion Criteria: All patients
Timing: Baseline; at time of procedure
Reporting Source: Clinical
Type: Numerical
Value Domain: quantity
Response Options: Numerical value of weight

Variable ID: weightunit
Variable: Body weight units
Definition: Units of body weight
Supporting Definition: None
Displayed Value Please indicate what units of measurement (kilograms or pounds) that you recorded your weight in.
Inclusion Criteria: All patients
Timing: Baseline; at time of procedure
Reporting Source: Clinical
Type: Singe answer
Value Domain: Code
Response Options: 1 = kilograms
2 = lbs

Variable ID: Anticoagulants
Variable: Anticoagulants
Definition: Anticoagulants currently being taken
Supporting Definition: None
Displayed Value Please indicate which anticogulant medication is currently being taken.
Inclusion Criteria: All patients
Timing: Baseline; at time of procedure
Reporting Source: Clinical
Type: Multiple answer
Value Domain: Code
Response Options: 1 = Thrombocyte Aggregation Inhibitors
2 = Cumarins
3 = DOACs
999 = unknown

Variable ID: ECA
Variable: Extracardiac arteriopathy
Definition: Please indicate if the patient has been diagnosed with extracardiac arteriopathy.
Supporting Definition: Including: claudication, carotid occlusion or >50% stenosis, amputation for arterial disease, previous or planned intervention on the abdominal aorta, limb arteries or carotids
Displayed Value Has the patient been diagnosed with extracardiac arteriopathy, including carotid and femoral?
Inclusion Criteria: All patients
Timing: Baseline; at time of procedure
Reporting Source: Clinical

**Type:**	Single answer
**Value Domain:**	Code
**Response Options:**	0= No<br>1= Yes<br>999= Unknown

**Variable ID:**	CPS
**Variable:**	Critical Preoperative State
**Definition:**	Critical preoperative state based on the Euro Score II definition.
**Supporting Definition:**	Ventricular tachycardia or ventricular fibrillation or aborted sudden death, preoperative cardiac massage, preoperative ventilation before anaesthetic room, preoperative inotropes or IABP, preoperative acute renal failure (anuria or oliguria <10ml/hr)
**Displayed Value**	Please indicate whether the patient has been in critical preoperative state at any point.
**Inclusion Criteria:**	All patients
**Timing:**	at time of procedure
**Reporting Source:**	Clinical
**Type:**	Single answer
**Value Domain:**	Code
**Response Options:**	0= No<br>1= Yes<br>999= Unknown

**Variable ID:**	PulmHT
**Variable:**	Pulmonary hypertension
**Definition:**	Please indicate if the patient has been diagnosed with pulmonary hypertension.
**Supporting Definition:**	Based on PA systolic pressure
**Displayed Value**	Please indicate if the patient has been diagnosed with pulmonary hypertension.
**Inclusion Criteria:**	All patients
**Timing:**	Baseline; at time of procedure
**Reporting Source:**	Clinical
**Type:**	Single answer
**Value Domain:**	Code
**Response Options:**	0= None /mild<br>1 = moderate: PA systolic pressure (31-55 mm Hg)<br>3 = severe: PA systolic pressure (>55mm Hg)<br>999= Unknown

**Variable ID:**	Dialysis
**Variable:**	Dialysis
**Definition:**	Please indicate if the patient is currently under any dialysis treatment.
**Supporting Definition:**	None
**Displayed Value**	Please indicate if the patient is currently under any dialysis treatment.
**Inclusion Criteria:**	All patients
**Timing:**	Baseline; at time of procedure
**Reporting Source:**	Clinical
**Type:**	Single answer
**Value Domain:**	Code
**Response Options:**	0= No<br>1= Yes<br>999= Unknown

**Variable ID:**	cardiovascularevent
**Variable:**	cardiovascular event
**Definition:**	has the patient been diagnosed with any cardiovascular event
**Supporting Definition:**	cardiovascular events of interest are acute myocardial infarction stroke excluding transient ischemic attacks and limb amputation excluding traumatic injury
**Displayed Value**	none
**Inclusion Criteria:**	All patients
**Timing:**	Baseline; at time of procedure
**Reporting Source:**	Clinical
**Type:**	Single answer
**Value Domain:**	Code
**Response Options:**	0 = No<br>1 = Yes<br>999 = Unknown

**Variable ID:**	myocardialinfarction
**Variable:**	myocardial infarction
**Definition:**	indicate whether the patient has a documented history of myocardial infarction
**Supporting Definition:**	item is phrased as a patient reported measure however if the patient is unable to answer this information can be abstracted from the medical records
**Displayed Value**	have you ever been told by your doctor that youve had a heart attack this is sometimes called a myocardial infarction or mi
**Inclusion Criteria:**	if 1 yes to cardiovascularevent
**Timing:**	Baseline; at time of procedure
**Reporting Source:**	Clinical
**Type:**	Single answer
**Value Domain:**	Code
**Response Options:**	0= No<br>1= Yes<br>999= Unknown

**Variable ID:**	PriorCardiacProcedure
**Variable:**	Prior cardiac procedure
**Definition:**	Please indicate if the patient has previously undergone cardiac procedure.
**Supporting Definition:**	None
**Displayed Value**	Please indicate if the patient has previously undergone cardiac procedure.
**Inclusion Criteria:**	All patients
**Timing:**	Baseline; at time of procedure
**Reporting Source:**	Clinical
**Type:**	Multiple answer
**Value Domain:**	Code
**Response Options:**	0= No<br>1= Yes, CABG<br>3= Yes, valve surgery<br>4= Yes, othter cardiac surgery<br>5= Yes, prior percutanous coronary procedure<br>6=Yes, prior percutaneous valve procedure<br>999= Unknown

**Variable ID:**	diabetesmellitus
**Variable:**	diabetes mellitus
**Definition:**	indicate if the patient has a documented history of diabetes mellitus regardless of duration of disease or need for anti-diabetic agents
**Supporting Definition:**	item is phrased as a patient reported measure however if the patient is unable to answer this information can be abstracted from the medical records
**Displayed Value**	have you ever been told by your doctor that you have diabetes
**Inclusion Criteria:**	All patients
**Timing:**	Baseline; at time of procedure
**Reporting Source:**	Clinical
**Type:**	Single answer
**Value Domain:**	Code
**Response Options:**	0 = No<br>1 = Yes<br>999 = Unknown

**Variable ID:**	insulin
**Variable:**	past medical history insulin dependent
**Definition:**	indicate if the patient is insulin dependent
**Supporting Definition:**	none
**Displayed Value**	none
**Inclusion Criteria:**	all patientsif answered 1 yes to diabetesmellitus
**Timing:**	Baseline; at time of procedure
**Reporting Source:**	Clinical
**Type:**	Single answer
**Value Domain:**	Code
**Response Options:**	0 = No<br>1 = Yes

**Variable ID:**	ChronicLungDis
**Variable:**	Chronic Lung Disease

**Definition:**	indicate whether the patient has a documented history or is currently diagnosed with chronic lung disease.
**Supporting Definition:**	Long term use of bronchodilators or steroids for lung disease.
**Displayed Value**	Has the patient ever been diagnosed with chronic lung disease?
**Inclusion Criteria:**	All patients
**Timing:**	Baseline; at time of procedure
**Reporting Source:**	Clinical
**Type:**	Single answer
**Value Domain:**	Code
**Response Options:**	0= No<br>1= Yes<br>999= Unknown
**Variable ID:**	nyha-q01
**Variable:**	question 1 of nyha
**Definition:**	patient symptoms
**Supporting Definition:**	nyha - new york heart association classification of heart failure functional capacity
**Displayed Value**	none
**Inclusion Criteria:**	All patients
**Timing:**	Baseline; at time of procedure
**Reporting Source:**	Clinical
**Type:**	Single answer
**Value Domain:**	Code
**Response Options:**	0 = Class I: No limitation of physical activity. Ordinary physical activity does not cause undue fatigue, palpitation, dyspnea (shortness of breath).<br>1 = Class II: Slight limitation of physical activity. Comfortable at rest. Ordinary physical activity res
**Variable ID:**	nyha-q02
**Variable:**	question 2 of nyha
**Definition:**	objective assessment of patient
**Supporting Definition:**	nyha - new york heart association classification of heart failure objective assessment by clinician
**Displayed Value**	none
**Inclusion Criteria:**	All patients
**Timing:**	Baseline; at time of procedure
**Reporting Source:**	Clinical
**Type:**	Single answer
**Value Domain:**	Code
**Response Options:**	0 = Class A: No objective evidence of cardiovascular disease. No symptoms and no limitation in ordinary physical activity.<br>1 = Class B: Objective evidence of minimal cardiovascular disease. Mild symptoms and slight limitation during ordinary activity. Com
**Variable ID:**	Prior_Endocarditis
**Variable:**	Prior_Endocarditis
**Definition:**	Please indicate whether the patient has ever been diagnosed with endocarditis or has active endocarditis at this moment. Endocarditis is diagnosed according to the modified duke criteria (reference). Active is defined as still under antibiotic treatment a
**Supporting Definition:**	Reference: Durack DT, Lukes AS, Bright DK. New criteria for diagnosis of infective endocarditis: utilization of<br>specific echocardiographic findings. Duke Endocarditis Service. Am J Med. 1994;96(3):200-9.
**Displayed Value**	Has the patient been diagnosed with endocarditis?
**Inclusion Criteria:**	All patients
**Timing:**	Baseline; at time of procedure
**Reporting Source:**	Clinical
**Type:**	Single answer
**Value Domain:**	Code
**Response Options:**	0= No<br>1= Yes, active<br>3= Yes, not active<br>999= Unknown
**Variable ID:**	CreatinineUnit

**Variable:**	Serum Creatinine units
**Definition:**	Indicate the units used for serum creatinine measurement
**Supporting Definition:**	None
**Displayed Value**	Indicate the units used for serum creatinine measurement
**Inclusion Criteria:**	All patients
**Timing:**	Baseline; at time of procedure
**Reporting Source:**	Clinical
**Type:**	Single answer
**Value Domain:**	code
**Response Options:**	1 = µmol/l<br>2 = mg/dl

**Variable ID:**	CreatinineValue
**Variable:**	Serum Creatinine
**Definition:**	Provide the serum creatinine value in the indicated units
**Supporting Definition:**	None
**Displayed Value**	Provide the serum creatinine value in the indicated units
**Inclusion Criteria:**	All patients
**Timing:**	Baseline; at time of procedure
**Reporting Source:**	Clinical
**Type:**	Numerical value
**Value Domain:**	quantity
**Response Options:**	None

**Variable ID:**	PoorMobility
**Variable:**	Poor mobility
**Definition:**	Poor mobility as defined according to ESII. ref: https://www.euroscore.org/index.php?id=17&lang=en
**Supporting Definition:**	Severe impairment of mobility secondary to musculoskeletal or neurological dysfunction.
**Displayed Value**	Does the patient have poor mobility?
**Inclusion Criteria:**	All patients
**Timing:**	Baseline; at time of procedure
**Reporting Source:**	Clinical
**Type:**	Single answer
**Value Domain:**	Code
**Response Options:**	0= No<br>1= Yes<br>999= Unknown

**Variable ID:**	CCS4
**Variable:**	CCS class 4 agina
**Definition:**	Angina at rest
**Supporting Definition:**	Angina classification according to the Canadian Cardiovascular Society grading. ref: https://www.euroscore.org/index.php?id=17&lang=en
**Displayed Value**	Has the patient been diagnosed with angina at rest?
**Inclusion Criteria:**	All patients
**Timing:**	Baseline; at time of procedure
**Reporting Source:**	Clinical
**Type:**	Single answer
**Value Domain:**	Code
**Response Options:**	0= No<br>1= Yes<br>999= Unknown

**Variable ID:**	Dissection
**Variable:**	Type A Dissection
**Definition:**	Type A dissection according to stanford criteria.
**Supporting Definition:**	Type A dissection is defined as a dissection proximal to the brachiocephalic artery.
**Displayed Value**	Has the patient a Type A aortic dissection at time of intervention
**Inclusion Criteria:**	Patients undergoing intervention
**Timing:**	at time of procedure
**Reporting Source:**	Clinical
**Type:**	Single answer
**Value Domain:**	Code
**Response Options:**	0= No

	1= Yes
	999= Unknown
**Variable ID:**	ESII
**Variable:**	Euroscore II
**Definition:**	Automtically caculated score from items YearofBirth, Sex, Creatinin, weightvalue, PoorMobility, CCS4, PriorCardiacProcedure, CPS, PulmHT, Dialysis, RMI, PriorCardiacProcedure, DiabetesOninsulin, ChronicLungDis, nyha, Endocarditis, LVF, UrgencyTreatment, M
**Supporting Definition:**	None
**Displayed Value**	None
**Inclusion Criteria:**	Patients undergoing intervention
**Timing:**	at time of procedure
**Reporting Source:**	Clinical
**Type:**	Numerical value
**Value Domain:**	quantity
**Response Options:**	None
**Variable ID:**	STSscore
**Variable:**	Society of Thoracic Surgeons score
**Definition:**	The STS score caculated according to: https://www.sts.org/resources/risk-calculator
**Supporting Definition:**	None
**Displayed Value**	None
**Inclusion Criteria:**	Patients undergoing intervention
**Timing:**	at time of procedure
**Reporting Source:**	Clinical
**Type:**	Numerical value
**Value Domain:**	quantity
**Response Options:**	None

Echocardiography general

**Variable ID:**	LVD
**Variable:**	Left ventricle dimensions
**Definition:**	Please indicate if the patient's left ventricle dimensions have been measured.
**Supporting Definition:**	None
**Displayed Value**	Have the patient's left ventricle dimensions been measured?
**Inclusion Criteria:**	All patients
**Timing:**	Index event; at time of procedure
**Reporting Source:**	Clinical
**Type:**	Single answer
**Value Domain:**	Code
**Response Options:**	0= No
	1= Yes
	999= Unknown
**Variable ID:**	LVD1
**Variable:**	Left ventricle dimensions: Follow-up question 1
**Definition:**	Please indicate which dimensions of the left ventricle have been measured.
**Supporting Definition:**	None
**Displayed Value**	Which dimensions of the left ventricle have been measured?
**Inclusion Criteria:**	If answered "1= Yes" to LVD
**Timing:**	Index event; at time of procedure
**Reporting Source:**	Clinical
**Type:**	Multiple answer
**Value Domain:**	Code
**Response Options:**	1 = LVESD (left ventricular end systolic diameter)
	2 = LVEDD (left ventricular end diastolic diameter)
	3 = IVSd (Intra ventricular septum diameter)
**Variable ID:**	LVD2
**Variable:**	Left ventricle dimensions: Follow-up question 2
**Definition:**	Please indicate the dimensions of the left ventricular end systolic diameter in mm.
**Supporting Definition:**	None

**Displayed Value**	Please indicate the dimensions of the left ventricular end systolic diameter in mm.
**Inclusion Criteria:**	If answered "1=LVESD (left ventricular end systolic diameter) to LVD1
**Timing:**	Index event; at time of procedure
**Reporting Source:**	Clinical
**Type:**	Numerical value
**Value Domain:**	quantity
**Response Options:**	None
**Variable ID:**	LVD3
**Variable:**	Left ventricle dimensions: Follow-up question 3
**Definition:**	Please indicate the dimensions of the left ventricular end diastolic diameter in mm.
**Supporting Definition:**	None
**Displayed Value**	Please indicate the dimensions of the left ventricular end diastolic diameter in mm.
**Inclusion Criteria:**	If answered "2=LVEDD (left ventricular end diastolic diameter) to LVD1.
**Timing:**	Index event; at time of procedure
**Reporting Source:**	Clinical
**Type:**	Numerical value
**Value Domain:**	quantity
**Response Options:**	None
**Variable ID:**	LVD4
**Variable:**	Left ventricle dimensions: Follow-up question 4
**Definition:**	Please indicate the dimensions of the intraventricular septum diameter in mm.
**Supporting Definition:**	None
**Displayed Value**	Please indicate the dimensions of the intraventricular septum diameter in mm.
**Inclusion Criteria:**	If answered "3=IVSd (Intra ventricular septum diameter) to LVD1.
**Timing:**	Index event; at time of procedure
**Reporting Source:**	Clinical
**Type:**	Numerical value
**Value Domain:**	quantity
**Response Options:**	None
**Variable ID:**	LVF
**Variable:**	Left ventricle function
**Definition:**	Please indicate the left ventricle function
**Supporting Definition:**	None
**Displayed Value**	Please indicatie the left ventricle function
**Inclusion Criteria:**	All patients
**Timing:**	Index event; at time of procedure
**Reporting Source:**	Clinical
**Type:**	Single answer
**Value Domain:**	Code
**Response Options:**	0 = good (LVEF >50%)<br>1= moderate (LVEF 31-50%)<br>2= poor (LVEF 21-30%)<br>3= very poor (=<20%)<br>999= unknown/not measured
**Variable ID:**	RVF
**Variable:**	Right ventricle function
**Definition:**	Please indicate the right ventricle function.
**Supporting Definition:**	None
**Displayed Value**	Please indicate the right ventricle function.
**Inclusion Criteria:**	All patients
**Timing:**	Index event; at time of procedure
**Reporting Source:**	Clinical
**Type:**	Single answer
**Value Domain:**	Code
**Response Options:**	0= normal<br>1= mildy impaired<br>2= moderately impared<br>3= severly impaired<br>999= unknown/not measured

Aortic valve details

Variable ID: NativeAV
Variable: Native aortic valve
Definition: Please indicatie if the patient still has an unrepaired native aortic valve or that a mechanical/bioprostheses/autograft/homograft is present.
Supporting Definition: None
Displayed Value Please indicatie if the patient still has an unrepaired native aortic valve or that a mechanical/bioprostheses is present.
Inclusion Criteria: All patients
Timing: Index event; at time of procedure
Reporting Source: Clinical
Type: Single answer
Value Domain: Code
Response Options: 0= Native valve
1= Prior AV repair
2= Prior AV replacement

Variable ID: AoDil
Variable: Aortic Dilatation
Definition: Please indicate if there is any aortic dilatation >40 mm.
Supporting Definition: None
Displayed Value Please indicate if there is any aortic dilatation >40 mm.
Inclusion Criteria: All patients
Timing: Index event; at time of procedure
Reporting Source: Clinical
Type: Multiple answer
Value Domain: Code
Response Options: 0= No
1= Yes, of aortic root
2= Yes, aortic ascendens
999 = unknown

Variable ID: AoDilMaxRoot
Variable: Maximum Diameter Aortic Root
Definition: Please indicate the maximum diameter of the aortic root in millimeters.
Supporting Definition: None
Displayed Value Please indicate the maximum diameter of the aortic root in millimeters.
Inclusion Criteria: If answered "2= Yes, aortic root" to AoDil
Timing: Index event; at time of procedure; (optional) annually
Reporting Source: Clinical
Type: Numerical value
Value Domain: quantity
Response Options: None

Variable ID: AoDilMaxAsc
Variable: Maximum diameter aortic ascendens
Definition: Please indicate the maximum diameter of the aortic ascendens in millimeters.
Supporting Definition: None
Displayed Value Please indicate the maximum diameter of the aortic ascendens in millimeters.
Inclusion Criteria: If answered "3= Yes, Aortic acendens"to AoDil
Timing: Index event; at time of procedure; (optional) annually
Reporting Source: Clinical
Type: Numerical value
Value Domain: quantity
Response Options: Numerical value of max diameter aortic ascendens in mm.

Variable ID: AVCusps
Variable: Aortic valve cusps
Definition: Please indicate the number of cusps detected in the aortic valve.
Supporting Definition: None
Displayed Value Please indicate the number of cusps detected in the aortic valve.
Inclusion Criteria: If answered "0= Native valve" OR "1= Prior repair" to NativeAV
Timing: Index event; at time of procedure
Reporting Source: Clinical

**Type:**	Single answer
**Value Domain:**	Code
**Response Options:**	1= Tricuspid<br>2= Bicuspid<br>3= Unicuspid<br>4= Quadricuspid<br>999 = Unknown

**Variable ID:**	AnnulusDiameter
**Variable:**	Annulus diameter
**Definition:**	Please indicate the annulus diameter measured.
**Supporting Definition:**	None
**Displayed Value**	Please indicate the annulus diameter measured.
**Inclusion Criteria:**	If answered "0= Native valve" OR "1= Prior repair" to NativeAV
**Timing:**	Index event; at time of procedure
**Reporting Source:**	Clinical
**Type:**	Numerical value
**Value Domain:**	quantity
**Response Options:**	Annulus size in mm

**Variable ID:**	AoValveDysfunction
**Variable:**	Aortic valve dysfunction
**Definition:**	Please indicate if aortic valve dysfunction is present.
**Supporting Definition:**	None
**Displayed Value**	Has the patient been diagnosed with aortic valve dysfunction?
**Inclusion Criteria:**	All patients
**Timing:**	Index event; at time of procedure
**Reporting Source:**	Clinical
**Type:**	Multiple answer
**Value Domain:**	Code
**Response Options:**	0= No<br>1= Yes, aortic valve stenosis<br>2= Yes aortic valve regurgitation

**Variable ID:**	AoVStenosis1
**Variable:**	Aortic valve velocity
**Definition:**	Please indicate the velocity of the flow overt the aortic valve.
**Supporting Definition:**	None
**Displayed Value**	What is the velocity of the blood flow over the aortic valve?
**Inclusion Criteria:**	If answered "1= Yes, aortic valve stenosis" to AoValveDysfunction
**Timing:**	Index event; at time of procedure
**Reporting Source:**	Clinical
**Type:**	Numerical value
**Value Domain:**	quantity
**Response Options:**	Numerical value of blood flow velocity in cm/s

**Variable ID:**	AoVStenosis2
**Variable:**	Aortic valve gradient
**Definition:**	Please indicate the gradient over the aortic valve.
**Supporting Definition:**	None
**Displayed Value**	What is the gradient over the aortic valve?
**Inclusion Criteria:**	If answered "1= Yes, aortic valve stenosis" to AoValveDysfunction
**Timing:**	Index event; at time of procedure
**Reporting Source:**	Clinical
**Type:**	Numerical value
**Value Domain:**	quantity
**Response Options:**	Numerical value of gradient in mm/Hg

**Variable ID:**	AoVStenosis3
**Variable:**	Aortic valve area
**Definition:**	Please indicate the aortic valve area.
**Supporting Definition:**	None
**Displayed Value**	What is the aortic valve area?
**Inclusion Criteria:**	If answered "1= Yes, aortic valve stenosis" to AoValveDysfunction
**Timing:**	Index event; at time of procedure
**Reporting Source:**	Clinical

**Type:**	Single answer
**Value Domain:**	Numerical value
**Response Options:**	Numerical value of valve area in cm2

**Variable ID:**	AoRegurg
**Variable:**	Aortic valve regurgitation
**Definition:**	Please indicate the grade of aortic valve regurgitation.
**Supporting Definition:**	Grade 0 to 4 according to Table AR
**Displayed Value**	What is the grade of aortic valve regurgitation?
**Inclusion Criteria:**	If answered "Yes, aortic valve regurgitation" to AoValveDysfunction
**Timing:**	Index event; at time of procedure
**Reporting Source:**	Clinical
**Type:**	Single answer
**Value Domain:**	Code
**Response Options:**	0 = None<br>1= Grade 1<br>2= Grade 2<br>3= Grade 3<br>4= Grade 4

**Variable ID:**	AVRegurgMech
**Variable:**	Aortic regurgitation mechanism classification
**Definition:**	Please indicate which classification the aortic regurgitation mechanism belongs to.
**Supporting Definition:**	According to Carpentiers functional AR classification: reference: Patrizio Lancellotti, Christophe Tribouilloy, Andreas Hagendorff, Bogdan A. Popescu, Thor Edvardsen, Luc A. Pierard, Luigi Badano, Jose L. Zamorano, On behalf of the Scientific Document Committee of the European Association of Cardiovascular Imaging: Thor Edvardsen, Oliver Bruder, Bernard Cosyns, Erwan Donal, Raluca Dulgheru, Maurizio Galderisi, Patrizio Lancellotti, Denisa Muraru, Koen Nieman, Rosa Sicari, Document reviewers: Erwan Donal, Kristina Haugaa, Giovanni La Canna, Julien Magne, Edyta Plonska, Recommendations for the echocardiographic assessment of native valvular regurgitation: an executive summary from the European Association of Cardiovascular Imaging, European Heart Journal - Cardiovascular Imaging, Volume 14, Issue 7, July 2013, Pages 611–644,
**Displayed Value**	Please indicate which classification the aortic regurgitation mechanism belongs to.
**Inclusion Criteria:**	If answered ("0= Native valve" OR "1= Prior repair") to NativeAV AND "1= Yes, Aortic valve regurgitation" to AoValveDysfunction
**Timing:**	Index event; at time of procedure
**Reporting Source:**	Clinical
**Type:**	Single answer
**Value Domain:**	Code
**Response Options:**	1= Type I<br>2= Type II<br>3= Type III

**Variable ID:**	AVDiseaseEtiology
**Variable:**	AV Disease etiology
**Definition:**	Please indicate which disease etiology has been found leading to regurgitation or stenosis.
**Supporting Definition:**	None
**Displayed Value**	Please indicate which disease etiology has been defined.
**Inclusion Criteria:**	If answered "1= Yes, aorti valve stenosis" OR "2= Yes, aortic valve regurgitation" to AoValveDysfunction
**Timing:**	Index event; at time of procedure
**Reporting Source:**	Clinical
**Type:**	Single answer
**Value Domain:**	Code
**Response Options:**	1= bicuspid/unicuspid/quadricuspid<br>2= congenital<br>3= degenerative/dystrofy<br>4= endocarditis<br>5= Rheumatic<br>6= Prostethic valve failure

Mitral valve details

**Variable ID:**	NativeMV
**Variable:**	Native mitral valve
**Definition:**	Please indicate if the patient still has an unrepaired native mitral valve or that a mechanical/bioprostheses is present.
**Supporting Definition:**	None
**Displayed Value**	Please indicate if the patient still has an unrepaired native mitral valve or that a mechanical/bioprostheses is present.
**Inclusion Criteria:**	All patients
**Timing:**	Index event; at time of procedure
**Reporting Source:**	Clinical
**Type:**	Single answer
**Value Domain:**	Code
**Response Options:**	0= Native valve<br>1= Prior MV repair<br>2= Prior MV replacement

**Variable ID:**	MVdysfunction
**Variable:**	Mitral valve dysfunction
**Definition:**	Please indicate if Mitral valve dysfunction is present.
**Supporting Definition:**	None
**Displayed Value**	Please indicate if Mitral valve dysfunction is present.
**Inclusion Criteria:**	All patients
**Timing:**	Index event; at time of procedure
**Reporting Source:**	Clinical
**Type:**	Multiple answer
**Value Domain:**	Code
**Response Options:**	0= No<br>1= Yes, mitral valve stenosis<br>2= Yes mitral valve regurgitation

**Variable ID:**	MVstenosis1
**Variable:**	Mitral valve stenosis velocity
**Definition:**	Please indicate the maximum valve velocity (cm/s).
**Supporting Definition:**	None
**Displayed Value**	Please indicate the maximum valve velocity (cm/s).
**Inclusion Criteria:**	If answered "1=Yes, mitral valve stenosis" to MVdysfunction
**Timing:**	Index event; at time of procedure
**Reporting Source:**	Clinical
**Type:**	Numerical value
**Value Domain:**	quantity
**Response Options:**	Numerical value of blood flow velocity in cm/s

**Variable ID:**	MVstenosis2
**Variable:**	Mitral valve stenosis gradient
**Definition:**	Please indicate the mean transvalvular gradient (mm/Hg)
**Supporting Definition:**	None
**Displayed Value**	Please indicate the mean transvalvular gradient (mm/Hg)
**Inclusion Criteria:**	If answered "1=Yes, mitral valve stenosis" to Mvdysfunction
**Timing:**	Index event; at time of procedure
**Reporting Source:**	Clinical
**Type:**	Numerical value
**Value Domain:**	quantity
**Response Options:**	Numerical value of gradient in mm/Hg

**Variable ID:**	Mvstenosis3
**Variable:**	Mitral valve stenosis valve area
**Definition:**	Please indicate the valve area (in cm2) of the mitral valve.
**Supporting Definition:**	None
**Displayed Value**	Please indicate the valve area (in cm2) of the mitral valve.
**Inclusion Criteria:**	If answered "1=Yes, mitral valve stenosis" to Mvdysfunction
**Timing:**	Index event; at time of procedure
**Reporting Source:**	Clinical
**Type:**	Numerical value
**Value Domain:**	quantity
**Response Options:**	Numerical value of valve area in cm2

**Variable ID:**	MVregurg
**Variable:**	Mitral valve reguritgation
**Definition:**	Please indicate which grade of regurgitation is present.
**Supporting Definition:**	Grade 0 to 4 according to Table MR
**Displayed Value**	Please indicate which grade of regurgitation is present.
**Inclusion Criteria:**	If answered "2= Yes, mitral valve regurgitation" to MV dysfunction
**Timing:**	Index event; at time of procedure
**Reporting Source:**	Clinical
**Type:**	Single answer
**Value Domain:**	Code
**Response Options:**	0 = None<br>1= Grade 1<br>2= Grade 2<br>3= Grade 3<br>4= Grade 4

**Variable ID:**	ModeMVRegurg
**Variable:**	Mode mitral valve regurgitation
**Definition:**	Please indicate the mode of mitral valve regurgitation.
**Supporting Definition:**	None
**Displayed Value**	Please indicate the mode of mitral valve regurgitation.
**Inclusion Criteria:**	If answered "2= Yes, mitral valve regurgitation" to MV dysfunction AND ("0=native valve" OR "1= Prior MV repair") to NativeMV
**Timing:**	Index event; at time of procedure
**Reporting Source:**	Clinical
**Type:**	Multiple answer
**Value Domain:**	Code
**Response Options:**	1= Primary<br>2= Functional/Secondary

**Variable ID:**	MechMVRegurg
**Variable:**	Mechanism mitral valve regurgitation (Carpentier)
**Definition:**	Please indicate which mechanism classification the mitral valve regurgitation belongs to according to the carpentier classification. Ref: https://doi.org/10.1016/j.jcmg.2018.01.009
**Supporting Definition:**	None
**Displayed Value**	Please indicate which mechanism classification the mitral valve regurgitation belongs to.
**Inclusion Criteria:**	If answered "2= Yes, mitral valve regurgitation" to MV dysfunction AND ("0=native valve" OR "1= Prior MV repair") to NativeMV
**Timing:**	Index event; at time of procedure
**Reporting Source:**	Clinical
**Type:**	Single answer
**Value Domain:**	Code
**Response Options:**	1= Type I<br>2= Type II<br>3= Type IIIa<br>4= Type IIIb

**Variable ID:**	MVLesion2
**Variable:**	Mitral Valve Lesion Leaflet
**Definition:**	Please indicate the location of the leaflet prolapse in case there is one.
**Supporting Definition:**	None
**Displayed Value**	Please indicate the location of the leaflet prolapse in case there is one.
**Inclusion Criteria:**	If answered "2= Yes, mitral valve regurgitation" to MV dysfunction AND ("0=native valve" OR "1= Prior MV repair") to NativeMV
**Timing:**	Index event; at time of procedure
**Reporting Source:**	Clinical
**Type:**	Single answer
**Value Domain:**	Code
**Response Options:**	1= Posterior<br>2= Bi-leaflet<br>3= Anterior<br>999 = Unknown

**Variable ID:**	MVDiseaseEtiology

**Variable:**	MV Disease etiology: follow-up question 1
**Definition:**	Please indicate which disease etiology has been defined.
**Supporting Definition:**	None
**Displayed Value**	Please indicate which disease etiology has been defined.
**Inclusion Criteria:**	If answered "2= Yes, mitral valve regurgitation" to MV dysfunction AND ("0=native valve" OR "1= Prior MV repair") to NativeMV
**Timing:**	Index event; at time of procedure
**Reporting Source:**	Clinical
**Type:**	Multiple answer
**Value Domain:**	Numerical value
**Response Options:**	1= Myxomatous degeneration/prolapse<br>2= Endocarditis<br>3= Rheumatic<br>4= Ischemic<br>5= Cardiomyopathy<br>6= Tumor<br>7= Trauma<br>8= Carcinoid<br>9= Congenital<br>10= Pure annular dilation<br>11= Reoperation for failure of previous MV repair/replacement<br>12= Acute papilary muscle

Tricuspid valve details

**Variable ID:**	NativeTV
**Variable:**	Native tricuspid valve
**Definition:**	Please indicate if the patient still has an unrepaired native tricuspid valve or that a mechanical/bioprostheses is present
**Supporting Definition:**	None
**Displayed Value**	Please indicate if the patient still has an unrepaired native mitral valve or that a mechanical/bioprostheses is present
**Inclusion Criteria:**	All patients
**Timing:**	Index event; at time of procedure
**Reporting Source:**	Clinical
**Type:**	Single answer
**Value Domain:**	Code
**Response Options:**	0= Native valve<br>1= Prior TV repair<br>2= Prior TV replacement

**Variable ID:**	TVdysfunction
**Variable:**	Tricuspid valve dysfunction
**Definition:**	Please indicate if tricuspid valve dysfunction is present
**Supporting Definition:**	None
**Displayed Value**	Please indicate if tricuspid valve dysfunction is present
**Inclusion Criteria:**	All patients
**Timing:**	Index event; at time of procedure
**Reporting Source:**	Clinical
**Type:**	Multiple answer
**Value Domain:**	Code
**Response Options:**	0= No<br>1= Yes, Tricuspid valve stenosis<br>2= Yes Tricuspid valve regurgitation

**Variable ID:**	TVregurg
**Variable:**	Tricuspid valve regurgitation
**Definition:**	Please indicate which grade of regurgitation is present
**Supporting Definition:**	Grade 0 to 3 according to Table TR
**Displayed Value**	Please indicate which grade of regurgitation is present
**Inclusion Criteria:**	If answered "2= Yes, tricuspid valve regurgitation" to TVdysfunction
**Timing:**	Index event; at time of procedure
**Reporting Source:**	Clinical

**Type:**	Single answer
**Value Domain:**	Code
**Response Options:**	0 = None<br>1= Grade 1<br>2= Grade 2<br>3= Grade 3

**Variable ID:**	TVStenosis
**Variable:**	Tricuspid Valve Stenosis: mean transvalvular gradient (mm/Hg)
**Definition:**	Please indicate the mean transvalvular gradient (mm/Hg)
**Supporting Definition:**	None
**Displayed Value**	Please indicate the mean transvalvular gradient (mm/Hg)
**Inclusion Criteria:**	If answered "1= Yes, tricuspid valve stenosis" to Tvdysfunction
**Timing:**	Index event; at time of procedure
**Reporting Source:**	Clinical
**Type:**	Single answer
**Value Domain:**	quantity
**Response Options:**	Gradient (mm/Hg)

**Variable ID:**	ModeTVregurg
**Variable:**	Mode tricuspid valve regurgitation
**Definition:**	Please indicate the mode of tricuspid valve regurgitation.
**Supporting Definition:**	None
**Displayed Value**	Please indicate the mode of mitral valve regurgitation.
**Inclusion Criteria:**	If answered "2= Yes, tricuspid valve regurgitation" to TVdysfunction AND ("0=native valve" OR "1= Prior TV repair") to NativeTV
**Timing:**	Index event; at time of procedure
**Reporting Source:**	Clinical
**Type:**	Multiple answer
**Value Domain:**	Code
**Response Options:**	1= Primary<br>2= Functional/Secondary

**Variable ID:**	TVDiseaseEtiology
**Variable:**	Tricuspid Valve Disease etiology
**Definition:**	Please indicate the TV disease eatiology.
**Supporting Definition:**	None
**Displayed Value**	Please indicate the TV disease eatiology.
**Inclusion Criteria:**	If answered ("2= Yes, tricuspid valve regurgitation" OR "1= Tricuspid valve stenosis") to TVdysfunction AND ("0=native valve" OR "1= Prior TV repair") to NativeTV
**Timing:**	Index event; at time of procedure
**Reporting Source:**	Clinical
**Type:**	Multiple answer
**Value Domain:**	Code
**Response Options:**	1= Endocarditis<br>2= Rheumatic<br>3= Tumor<br>4= Trauma<br>5= Carcinoid<br>6= Congenital<br>7= Pure annular dilation<br>8= Reoperation for failure of previous TV repair/replacement<br>9= Pacemaker lead<br>999= unknown

Hartcath

**Variable ID:**	HeartCath
**Variable:**	Heart catheterization
**Definition:**	Please indicate a heart catherisation has been performed.
**Supporting Definition:**	None
**Displayed Value**	Please indicate a heart catherisation has been performed.
**Inclusion Criteria:**	All patients

**Timing:**	Index event; at time of procedure
**Reporting Source:**	Clinical
**Type:**	Single answer
**Value Domain:**	Code
**Response Options:**	0= No<br>1= Yes<br>999= Unknown

**Variable ID:**	No_DisVess
**Variable:**	Number of diseased coronary vessels
**Definition:**	Please indicate the number of diseased vessels
**Supporting Definition:**	None
**Displayed Value**	Please indicate the number of diseased vessels
**Inclusion Criteria:**	If answered "1= Yes"to HeartCath
**Timing:**	Index event; at time of procedure
**Reporting Source:**	Clinical
**Type:**	Single answer
**Value Domain:**	Code
**Response Options:**	0 = None<br>1 = One<br>2 = Two<br>3 = Three

**Variable ID:**	LMS
**Variable:**	Left Main Stenosis
**Definition:**	Please indicate whether the patient has been diagnosed with left main stenosis >50%.
**Supporting Definition:**	>50%
**Displayed Value**	Please indicate whether the patient has been diagnosed with left main stenosis >50%.
**Inclusion Criteria:**	If answered "1= Yes"to HeartCath
**Timing:**	Index event; at time of procedure
**Reporting Source:**	Clinical
**Type:**	Single answer
**Value Domain:**	Code
**Response Options:**	0= No<br>1= Yes<br>999= Unknown

**Variable ID:**	BaselineRhythm
**Variable:**	Baseline rhythm
**Definition:**	Please indicate which baseline rhythm was detected.
**Supporting Definition:**	None
**Displayed Value**	Please indicate which baseline rhythm was detected.
**Inclusion Criteria:**	All patients
**Timing:**	Index event; at time of procedure
**Reporting Source:**	Clinical
**Type:**	Single answer
**Value Domain:**	Code
**Response Options:**	1 = Sinus<br>2 = Atrium flutter<br>3 = Atrial fibrillation<br>4 = Paced<br>999 =Unknown/not measured

Treatment

**Variable ID:**	Treatment
**Variable:**	Treatment
**Definition:**	Please indicate if a medical or intervention is performed
**Supporting Definition:**	None
**Displayed Value**	Is the valve disease treated medically or with surgical/transcathether intervention?
**Inclusion Criteria:**	All patients
**Timing:**	Index event; at time of procedure
**Reporting Source:**	Clinical
**Type:**	Single answer

**Value Domain:**	Code
**Response Options:**	0= No<br>1=Yes, medical treatment<br>2= Yes, percutanous treatment<br>3= yes, surgical treatment
**Variable ID:**	UrgencyTreatment
**Variable:**	Urgency of treatment
**Definition:**	Please indicate the treatment urgency
**Supporting Definition:**	elective : routine admission for operation.<br>urgent: patients who have not been electively admitted for operation but who require intervention or surgery on the current admission for medical reasons. These patients cannot be sent home without a definitive procedure.<br>emergency: operation before the beginning of the next working day after decision to operate.<br>salvage: patients requiring cardiopulmonary resuscitation (external cardiac massage) en route to the operating theatre or prior to induction of anaesthesia. This does not include cardiopulmonary resuscitation following induction of anaesthesia
**Displayed Value**	Please indicate the treatment urgency
**Inclusion Criteria:**	If answered "2= Yes, percutanous treatment" OR "3= yes, surgical treatment" to Treatment
**Timing:**	at time of procedure
**Reporting Source:**	Clinical
**Type:**	Single answer
**Value Domain:**	Code
**Response Options:**	0=Elective<br>1= Urgent<br>2= Emergency<br>3= Salvage
**Variable ID:**	ValveTreatment
**Variable:**	Valve treatment
**Definition:**	Please specify: on which valve was a procedure performed?
**Supporting Definition:**	None
**Displayed Value**	On which valve was the procedure/surgery performed?
**Inclusion Criteria:**	If answered ("2= Yes, percutanous treatment" OR "3= yes, surgical treatment") on Treatment
**Timing:**	at time of procedure
**Reporting Source:**	Clinical
**Type:**	Multiple answer
**Value Domain:**	Code
**Response Options:**	1= Aortic valve<br>2= Mitral valve<br>3= Tricuspid valve
**Variable ID:**	TreatmentDate
**Variable:**	Date of treatment
**Definition:**	Please indicate the date of the event
**Supporting Definition:**	None
**Displayed Value**	Please indicate the date of the event
**Inclusion Criteria:**	If answered ("2= Yes, percutanous treatment" OR "3= yes, surgical treatment") on Treatment
**Timing:**	at time of procedure
**Reporting Source:**	Clinical
**Type:**	Date by DD/MM/YYYY
**Value Domain:**	date
**Response Options:**	Date (DD-MM-YYYY)
**Variable ID:**	ACC
**Variable:**	Cross clamp time
**Definition:**	Please indicate the cross clamp time.
**Supporting Definition:**	Please enter 0 if no cross clamping was performed
**Displayed Value**	Please indicate the cross clamp time.
**Inclusion Criteria:**	If answered ("3= yes, surgical treatment") on Treatment
**Timing:**	at time of procedure
**Reporting Source:**	Clinical

**Type:**	Numerical value
**Value Domain:**	quantity
**Response Options:**	Time in minutes
**Variable ID:**	CPB
**Variable:**	Cardiopulmonary bypass time
**Definition:**	Please indicate the cardiopulmonary bypass time
**Supporting Definition:**	Please enter 0 if no cardiopulmonary bypass was performed
**Displayed Value**	Please indicate the cardiopulmonary bypass time
**Inclusion Criteria:**	If answered "3= yes, surgical treatment" on Treatment
**Timing:**	at time of procedure
**Reporting Source:**	Clinical
**Type:**	Numerical value
**Value Domain:**	quantity
**Response Options:**	Time in minutes
**Variable ID:**	SurgeryAccess
**Variable:**	Surgical access site
**Definition:**	Please indicate which surgical access site was used.
**Supporting Definition:**	None
**Displayed Value**	Please indicate which surgical access side was used.
**Inclusion Criteria:**	If answered "3= yes, surgical treatment" on Treatment
**Timing:**	at time of procedure
**Reporting Source:**	Clinical
**Type:**	Single answer
**Value Domain:**	Code
**Response Options:**	1= Full sternotomy<br>2 = Thoracotomy<br>3 = Mini sternotomy: J/U shape
**Variable ID:**	TCAcces
**Variable:**	Transcatheter intervention: follow-up question 1
**Definition:**	Please indicate the access site used for the transcatheter intervention.
**Supporting Definition:**	None
**Displayed Value**	Please indicate the access side used for the transcatheter intervention.
**Inclusion Criteria:**	If answered "2= Yes, percutanous treatment" on Treatment
**Timing:**	at time of procedure
**Reporting Source:**	Clinical
**Type:**	Single answer
**Value Domain:**	Code
**Response Options:**	1= Transapical<br>2= Transaxillary<br>3= Transfemoral<br>4= Transaortic<br>5= Subclavian<br>6= Transiliac<br>7= Transseptal<br>8= Transcarotid<br>9= Transcaval<br>888 = Other
**Variable ID:**	ProcedureTime
**Variable:**	Procedure time
**Definition:**	Please indicate the procedure time of the percutanous intervention
**Supporting Definition:**	None
**Displayed Value**	Please indicate the procedure time for the percutanous intervention
**Inclusion Criteria:**	If answered "2= Yes, percutanous treatment" on Treatment
**Timing:**	at time of procedure
**Reporting Source:**	Clinical
**Type:**	Numerical value
**Value Domain:**	quantity
**Response Options:**	Time in minutes
**Variable ID:**	ConcomitantQx
**Variable:**	Concomitant surgery

**Definition:**	Please indicate if the patient had concomitant surgery.
**Supporting Definition:**	None
**Displayed Value**	Please indicate if the patient had concomitant surgery.
**Inclusion Criteria:**	If answered "3= yes, surgical treatment" on Treatment
**Timing:**	at time of procedure
**Reporting Source:**	Clinical
**Type:**	Single answer
**Value Domain:**	Code
**Response Options:**	0= None<br>1= Yes, coronary artery bypass grafting<br>3= Yes, atrium ablation (MAZE) procedure<br>4= Aortic (hemi) arch surgery
**Variable ID:**	TCIembolicPD
**Variable:**	Transcatheter intervention: follow-up question 2
**Definition:**	Please indicate whether an emoblic protective device was used.
**Supporting Definition:**	None
**Displayed Value**	Please indicate whether an emoblic protective device was used.
**Inclusion Criteria:**	If answered "2= Yes, percutanous treatment" on Treatment
**Timing:**	At time of procedure
**Reporting Source:**	Clinical
**Type:**	Single answer
**Value Domain:**	Code
**Response Options:**	0= No<br>1= Yes<br>999= Unknown

AV intervention

**Variable ID:**	TypeAVsurgery
**Variable:**	Type of aortic valve surgery
**Definition:**	Please indicate if the aortic valve was repaired or replaced.
**Supporting Definition:**	None
**Displayed Value**	Please indicate if the aortic valve was repaired or replaced
**Inclusion Criteria:**	If answered "3= Yes, surgical treatment" on Treatment AND "1= Aortic Valve" on ValveTreatment
**Timing:**	at time of procedure
**Reporting Source:**	Clinical
**Type:**	Single answer
**Value Domain:**	Code
**Response Options:**	1= Aortic valve replacement<br>2= Aortic valve repair
**Variable ID:**	AoValveReplaceType
**Variable:**	Aortic Valve Replacement: type
**Definition:**	Please indicate the aortic valve replacement type.
**Supporting Definition:**	None
**Displayed Value**	Please indicate the aortic valve replacement type.
**Inclusion Criteria:**	If answered "1= AV replacement"to TypeAVsurgery
**Timing:**	at time of procedure
**Reporting Source:**	Clinical
**Type:**	Single answer
**Value Domain:**	Code
**Response Options:**	1= Isolated valve replacement<br>2= Tubular aorta + valve replacement<br>3= Root + valve replacement
**Variable ID:**	AoValveReplaceProsthesis
**Variable:**	Aortic Valve Replacement: prothesis
**Definition:**	Please indicate the aortic valve prosthesis type.
**Supporting Definition:**	None
**Displayed Value**	Please indicate the aortic valve prosthesis type.
**Inclusion Criteria:**	If answered "1= Isolated valve replacement" to AoValveReplaceType or "0= No" to AoValveReplacePrefacturedValveGraft.

**Timing:**	at time of procedure
**Reporting Source:**	Clinical
**Type:**	Single answer
**Value Domain:**	Code
**Response Options:**	1= Mechanical<br>2= Biological<br>3= Surgeon fashioned (Ozaki)<br>4= Ross<br>5= Homograft
**Variable ID:**	AoValveReplaceMan_Mech
**Variable:**	Aortic Valve Replacement: Manufacturer mechanical valve
**Definition:**	Please indicate the aortic valve replacement manufacturer.
**Supporting Definition:**	None
**Displayed Value**	Please indicate the aortic valve replacement manufacturer.
**Inclusion Criteria:**	If answered "1= Mechanical" to AoValveReplaceProsthesis.
**Timing:**	at time of procedure
**Reporting Source:**	Clinical
**Type:**	Single answer
**Value Domain:**	Code
**Response Options:**	1= Medtronic<br>2= Abbot<br>3= Sorin Liva Nova<br>4= On-X<br>888 = Other
**Variable ID:**	AVReplaceMedtronicModel_Mech
**Variable:**	Aortic Valve Replacement: Medtronic Model Mechanical
**Definition:**	Please indicate the valve model that was used.
**Supporting Definition:**	Aortic valve replacement: Medtronic mechanical prosthesis
**Displayed Value**	Please indicate the valve model that was used.
**Inclusion Criteria:**	If answered "1= Medtronic" to AoValveReplaceMan_Mech
**Timing:**	at time of procedure
**Reporting Source:**	Clinical
**Type:**	Single answer
**Value Domain:**	Code
**Response Options:**	1= ATS Open Pivot Aortic Standard<br>2= ATS Open Pivot AP<br>3= ATS Open Pivot AP 360<br>888 = Other
**Variable ID:**	AVReplaceAbbotModel_Mech
**Variable:**	Aortic Valve Replacement: Abbot Model Mechanical
**Definition:**	Please indicate the valve model that was used.
**Supporting Definition:**	Aortic valve replacement: Abbot Mechanical prosthesis
**Displayed Value**	Please indicate the valve model that was used.
**Inclusion Criteria:**	If answered "2= Abbot" to AoValveReplaceMan_Mech
**Timing:**	at time of procedure
**Reporting Source:**	Clinical
**Type:**	Single answer
**Value Domain:**	Code
**Response Options:**	1= Regent<br>2= Regent Flexcuff<br>3= Masters<br>4= Masters HP<br>5= Masters Series 15<br>6= Masters MECJ<br>888 = Other
**Variable ID:**	AVReplaceSorinLiveNovaModel_Mech
**Variable:**	Aortic Valve Replacement: Sorin Liva Nova Model Mechanical
**Definition:**	Please indicate the valve model that was used.
**Supporting Definition:**	Aortic valve replacement: Sorin Liva Nova Mechanical prosthesis
**Displayed Value**	Please indicate the valve model that was used.
**Inclusion Criteria:**	If answered "3= Sorin Liva Nova" to AoValveReplaceMan_Mech

**Timing:**	at time of procedure
**Reporting Source:**	Clinical
**Type:**	Single answer
**Value Domain:**	Code
**Response Options:**	1= Carbomedics Standard<br>2= Carbomedics Top-Hat<br>3= Carbomedics Reduced<br>4= Carbomedics Pediatric<br>5= Bicarbon Slimline<br>6= Bicarbon Overline<br>7= Bicarbon Fitline<br>8= Carbomedics Standard Mitral<br>9= Carbomedics Optiform<br>10= Bicarbon Fitline Mitral<br>11= Cardio

**Variable ID:**	AVReplaceOnX_Mech
**Variable:**	Aortic Valve Replacement: On-X Model Mechanical
**Definition:**	Please indicate the valve model that was used.
**Supporting Definition:**	Aortic valve replacement: On-X Mechanical prosthesis
**Displayed Value**	Please indicate the valve model that was used.
**Inclusion Criteria:**	If answered "4= On-X" to AoValveReplaceMan_Mech
**Timing:**	at time of procedure
**Reporting Source:**	Clinical
**Type:**	Single answer
**Value Domain:**	Code
**Response Options:**	1= On-X Standard<br>2= On-X Conform X<br>3= On-X Anatomic<br>888= Other

**Variable ID:**	AVReplaceOther_Mech
**Variable:**	Aortic Valve Replacement: Other Mechanical
**Definition:**	Please indicate the valve model that was used.
**Supporting Definition:**	Aortic valve replacement: other mechanical prosthesis manufacturer
**Displayed Value**	Please indicate the valve model that was used.
**Inclusion Criteria:**	If answered "888= Other" to AoValveReplaceMan_mech or "11= Other" to AoValveReplaceProsthesis1.
**Timing:**	at time of procedure
**Reporting Source:**	Clinical
**Type:**	Free text
**Value Domain:**	String
**Response Options:**	None

**Variable ID:**	AoValveReplacePrefacturedValveGraft
**Variable:**	Aortic Valve Replacement: prefactured valve and graft
**Definition:**	Please indicate if a prefactured valve and vascular protheses used.
**Supporting Definition:**	None
**Displayed Value**	Was a prefactured valve and vascular prostheses used?
**Inclusion Criteria:**	If answered "2= Tubular aorta + valve replacement" or "3= Root+ valve replacement" to AoValveReplaceType
**Timing:**	at time of procedure
**Reporting Source:**	Clinical
**Type:**	Single answer
**Value Domain:**	Code
**Response Options:**	0= No<br>1= Yes<br>999= Unknown

**Variable ID:**	AoValveReplaceProsthesis1
**Variable:**	Aortic Valve Replacement: prothesis for prefactured valve
**Definition:**	Please indicate the aortic valve prosthesis type.
**Supporting Definition:**	None
**Displayed Value**	Please indicate the aortic valve prosthesis type.
**Inclusion Criteria:**	If answered "1= Yes" to AoValveReplacePrefacturedValve.

**Timing:**	at time of procedure
**Reporting Source:**	Clinical
**Type:**	Single answer
**Value Domain:**	Code
**Response Options:**	1= Freestyle Stentless<br>2= SJM Masters Valved graft with Gelweave Valsalva technology<br>3= SJM Masters HP Valved graft with Gelweave Valsalva technology<br>4= Carboseal<br>5= Carboseal Valsalva<br>6= Mitroflow Valsalva<br>7= On-X ascending aortic prosthesis<br>8= Tube valv

**Variable ID:**	AoValveReplaceMan_Bio
**Variable:**	Aortic Valve Replacement: Manufacturer biological valve
**Definition:**	Please indicate the aortic valve replacement manufacturer.
**Supporting Definition:**	None
**Displayed Value**	Please indicate the aortic valve replacement manufacturer.
**Inclusion Criteria:**	If answered "2= Biological" to AoValveReplaceProsthesis or "0= No" to AoValveReplacePrefacturedValveGraft
**Timing:**	at time of procedure
**Reporting Source:**	Clinical
**Type:**	Single answer
**Value Domain:**	Code
**Response Options:**	1= Medtronic<br>2= Abbot<br>3= Sorin Liva Nova<br>4= Edwards<br>5= Labcor<br>6= Meril<br>888= Other

**Variable ID:**	AVReplaceMedtronicModel_Bio
**Variable:**	Aortic Valve Replacement: Medtronic Model Biological
**Definition:**	Please indicate the valve model that was used.
**Supporting Definition:**	Aortic valve replacement: Medtronic biological prosthesis
**Displayed Value**	Please indicate the valve model that was used.
**Inclusion Criteria:**	If answered "1= Medtronic" to AoValveReplaceMan_Bio
**Timing:**	at time of procedure
**Reporting Source:**	Clinical
**Type:**	Single answer
**Value Domain:**	Code
**Response Options:**	1= Mosaïc<br>2= Mosaïc Ultra<br>3= Hancock II<br>4= Hancock II Ultra<br>5= Avalus<br>6= Corevalve<br>7= Corevalve Evolut-R<br>8= Corevalve Evolut Pro<br>888= Other

**Variable ID:**	AVReplaceAbbotModel_Bio
**Variable:**	Aortic Valve Replacement: Abbot Model Biological
**Definition:**	Please indicate the valve model that was used.
**Supporting Definition:**	Aortic valve replacement: Abbot biological prosthesis
**Displayed Value**	Please indicate the valve model that was used.
**Inclusion Criteria:**	If answered "2= Abbot" to AoValveReplaceMan_Bio
**Timing:**	at time of procedure
**Reporting Source:**	Clinical
**Type:**	Single answer
**Value Domain:**	Code
**Response Options:**	1= Epic<br>2= Epic Supra

	3= Epic plus supra<br>4= Trifecta<br>5= Trifecta glide<br>6= Biocor<br>7= Biocor supra<br>888 = Other
**Variable ID:**	AVReplaceSorinLiveNovaModel_Bio
**Variable:**	Aortic Valve Replacement: Sorin Liva Nova Model Biological
**Definition:**	Please indicate the valve model that was used.
**Supporting Definition:**	Aortic valve replacement: Sorin Liva Nova Biological prosthesis
**Displayed Value**	Please indicate the valve model that was used.
**Inclusion Criteria:**	If answered "3= Sorin Liva Nova" to AoValveReplaceMan_Bio
**Timing:**	at time of procedure
**Reporting Source:**	Clinical
**Type:**	Single answer
**Value Domain:**	Code
**Response Options:**	1= Crown PRT<br>2= Solo Smart Sutureless<br>3= Perceval sutureless<br>4= Pericarbon more<br>888 = Other
**Variable ID:**	AVReplaceEdwardsModel_Bio
**Variable:**	Aortic Valve Replacement: Edwards Model Biological
**Definition:**	Please indicate the valve model that was used.
**Supporting Definition:**	Aortic valve replacement: Edwards Biological prosthesis
**Displayed Value**	Please indicate the valve model that was used.
**Inclusion Criteria:**	If answered "4= Edwards" to AoValveReplaceMan_Bio
**Timing:**	at time of procedure
**Reporting Source:**	Clinical
**Type:**	Single answer
**Value Domain:**	Code
**Response Options:**	1= Perimount Aortic CEP 2900<br>2= Perimount Magna Ease Aortic 3300 TFX<br>3= Inspiris Resilia 11500A<br>4= Perimount Mitral CEP Plus 6900 PC<br>5= Perimount Magna Ease Mitral 7300 TFX<br>888= Other
**Variable ID:**	AVReplaceLabcorModel_Bio
**Variable:**	Aortic Valve Replacement: Labcor Model Biological
**Definition:**	Please indicate the valve model that was used.
**Supporting Definition:**	Aortic valve replacement: Labcor Biological prosthesis
**Displayed Value**	Please indicate the valve model that was used.
**Inclusion Criteria:**	If answered "5= Labcor" to AoValveReplaceMan_Bio
**Timing:**	at time of procedure
**Reporting Source:**	Clinical
**Type:**	Single answer
**Value Domain:**	Code
**Response Options:**	1= Labcor TLBP<br>2= Dokimos plus<br>888= Other
**Variable ID:**	AVReplaceMericalModel_Bio
**Variable:**	Aortic Valve Replacement: Meril Model Biological
**Definition:**	Please indicate the valve model that was used.
**Supporting Definition:**	Aortic valve replacement:Meril Biological prosthesis
**Displayed Value**	Please indicate the valve model that was used.
**Inclusion Criteria:**	If answered "6= Meril" to AoValveReplaceMan_Bio
**Timing:**	at time of procedure
**Reporting Source:**	Clinical
**Type:**	Single answer
**Value Domain:**	Code
**Response Options:**	1= Dafodil

888= Other

Variable ID: AoValveReplaceSize
Variable: Aortic Valve Replacement: Size
Definition: Please indicate the aortic valve replacement valve prosthesis label size.
Supporting Definition: None
Displayed Value Please indicate the aortic valve replacement prosthesis label size.
Inclusion Criteria: If answered "1= AV replacement"to TypeAVsurgery AND ("1= Mechanical"OR "2= Biological") to AoValveReplaceProsthesis OR "1= AV replacement"to TypeAVsurgery AND AoValveReplacePrefacturedValve = yes
Timing: at time of procedure
Reporting Source: Clinical
Type: Numerical value
Value Domain: quantity
Response Options: Size in mm

Variable ID: IntentionrepairAV
Variable: Intention repair
Definition: Please indicate whether the AV was replaced because of a failed repair during the same surgery.
Supporting Definition: None
Displayed Value Please indicate whether the AV was replaced because of a failed repair during the same surgery.
Inclusion Criteria: If answered "1= AV replacement"to TypeAVsurgery AND ("1= Mechanical"OR "2= Biological") to AoValveReplaceProsthesis
Timing: at time of procedure
Reporting Source: Clinical
Type: Single answer
Value Domain: Code
Response Options: 0= No
1= Yes
999= Unknown

Variable ID: AoValveRepair
Variable: Aortic Valve Repair
Definition: Please indicate which AV repair technique was used.
Supporting Definition: None
Displayed Value Please indicatie which AV repair technique was used.
Inclusion Criteria: If answered "2= AV repair"to TypeAVsurgery
Timing: at time of procedure
Reporting Source: Clinical
Type: Single answer
Value Domain: Code
Response Options: 1= AV reimplantation
2= Root remodeling

Variable ID: AVPercutInt_Man
Variable: Aortic Valve percutaneous intervention device: Manufacturer
Definition: Please indicate the device manufacturere for the replacement/repair valve used in the percutaneous intervention.
Supporting Definition: None
Displayed Value Please indicate the device manufacturere for the replacement/repair valve used in the percutaneous intervention.
Inclusion Criteria: If answerd "2= yes, percutanous treatment" to treatment AND "1= Aortic valve" to ValveTreatment.
Timing: at time of procedure
Reporting Source: Clinical
Type: Single answer
Value Domain: Code
Response Options: 1= Medtronic
2= Abbot
3= Boston Scientific
4= New Valve Technology
5= Edwards
888= Other

**Variable ID:**	AVPercutIntModel_Medtronic
**Variable:**	Aortic Valve percutaneous intervention device: Medtronic Model
**Definition:**	Please indicate the valve model that was used.
**Supporting Definition:**	Aortic Valve percutaneous intervention device: Medtronic Model
**Displayed Value**	Please indicate the valve model that was used.
**Inclusion Criteria:**	If answered "1= Medtronic" to AVPercutInt_Man
**Timing:**	at time of procedure
**Reporting Source:**	Clinical
**Type:**	Single answer
**Value Domain:**	Code
**Response Options:**	1= Evolut R<br>2= Evolut Pro<br>888= Other

**Variable ID:**	AVPercutIntModel_Abbot
**Variable:**	Aortic Valve percutaneous intervention device: Abbot Model
**Definition:**	Please indicate the valve model that was used.
**Supporting Definition:**	Aortic Valve percutaneous intervention device: Abbot Model
**Displayed Value**	Please indicate the valve model that was used.
**Inclusion Criteria:**	If answered "2= Abbot" to AVPercutInt_Man
**Timing:**	at time of procedure
**Reporting Source:**	Clinical
**Type:**	Single answer
**Value Domain:**	Code
**Response Options:**	1= Mitraclip<br>2= Triclip<br>3= Portico<br>888= Other

**Variable ID:**	AVPercutIntModel_BostonSci
**Variable:**	Aortic Valve percutaneous intervention device: Boston Scientific Model
**Definition:**	Please indicate the valve model that was used.
**Supporting Definition:**	Aortic Valve percutaneous intervention device: Boston Scientific Model
**Displayed Value**	Please indicate the valve model that was used.
**Inclusion Criteria:**	If answered "3= Boston Scientific" to AVPercutInt_Man
**Timing:**	at time of procedure
**Reporting Source:**	Clinical
**Type:**	Single answer
**Value Domain:**	Code
**Response Options:**	1= Accurate Neo<br>2= Accurate Neo2<br>3= Portico<br>888= Other

**Variable ID:**	AVPercutIntModel_NVT
**Variable:**	Aortic Valve percutaneous intervention device: New Valve Technology Model
**Definition:**	Please indicate the valve model that was used.
**Supporting Definition:**	Aortic Valve percutaneous intervention device: New Valve Technology Model
**Displayed Value**	Please indicate the valve model that was used.
**Inclusion Criteria:**	If answered "4= New Valve Technology" to AVPercutInt_Man
**Timing:**	at time of procedure
**Reporting Source:**	Clinical
**Type:**	Single answer
**Value Domain:**	Code
**Response Options:**	1= Allegra<br>888= Other

**Variable ID:**	AVPercutIntModel_Edwards
**Variable:**	Aortic Valve percutaneous intervention device: Edwards Model
**Definition:**	Please indicate the valve model that was used.
**Supporting Definition:**	Aortic Valve percutaneous intervention device: Edwards Model
**Displayed Value**	Please indicate the valve model that was used.
**Inclusion Criteria:**	If answered "5= Edwards" to AVPercutInt_Man
**Timing:**	at time of procedure
**Reporting Source:**	Clinical

**Type:**	Single answer
**Value Domain:**	Code
**Response Options:**	1= Sapien 3<br>2= Sapien XT<br>888= Other

**Variable ID:**	AoValvePercutIntSize
**Variable:**	Aortic Valve Percutaneous Intervention Valve Size
**Definition:**	Please indicate the aortic valve replacement/repair label size.
**Supporting Definition:**	None
**Displayed Value**	Please indicate the aortic valve replacement/repair label size.
**Inclusion Criteria:**	If answered "2= yes, percutanous treatment" to treatment AND "1= Aortic valve" to ValveTreatment.
**Timing:**	at time of procedure
**Reporting Source:**	Clinical
**Type:**	Numerical value
**Value Domain:**	quantity
**Response Options:**	Size in mm

**Variable ID:**	TCIdeviceOtherAV
**Variable:**	Name and manufacturer device aortic valve
**Definition:**	Please indicate the name/manufacturer of the transcatheter AV device if it does not appear in the list provided.
**Supporting Definition:**	None
**Displayed Value**	Please indicate the name/manufacturer of the transcatheter AV device if it does not appear in the list provided.
**Inclusion Criteria:**	if answered "888= Other" to AVPercutInt_Man
**Timing:**	at time of procedure
**Reporting Source:**	Clinical
**Type:**	Free text
**Value Domain:**	String
**Response Options:**	None

**Variable ID:**	TCInvalve
**Variable:**	Transcatheter intervention: follow-up question 3
**Definition:**	Please indicate if a second valve was implanted during the transcatheter implantation.
**Supporting Definition:**	None
**Displayed Value**	Please indicate if a second valve was implanted during the transcatheter implantation.
**Inclusion Criteria:**	If answered "2= yes, percutanous treatment" to treatment AND "1= Aortic valve" to ValveTreatment.
**Timing:**	at time of procedure
**Reporting Source:**	Clinical
**Type:**	Single answer
**Value Domain:**	Code
**Response Options:**	0= No<br>1= Yes<br>999= Unknown

MV Intervention

**Variable ID:**	TypeMVsurgery
**Variable:**	Type of mitral valve surgery
**Definition:**	Please indicate if the MV was repaired or replaced
**Supporting Definition:**	None
**Displayed Value**	Please indicatie if the MV was repaired or replaced
**Inclusion Criteria:**	If answered "3= Yes, surgical treatment" on Treatment AND "2= Mitral Valve" on ValveTreatment
**Timing:**	at time of procedure
**Reporting Source:**	Clinical
**Type:**	Singe answer
**Value Domain:**	Code
**Response Options:**	1= MV replacement<br>2= MV repair

**Variable ID:**	MVReplaceProsthesis

**Variable:**	Mitral Valve Replacement: follow-up question 1
**Definition:**	Please indicate the valve prosthesis type.
**Supporting Definition:**	None
**Displayed Value**	Please indicate the valve prosthesis type.
**Inclusion Criteria:**	If answered "1= MV replacement"to TypeMVsurgery
**Timing:**	at time of procedure
**Reporting Source:**	Clinical
**Type:**	Single answer
**Value Domain:**	Code
**Response Options:**	1= Mechanical<br>2= Bioprosthesis
**Variable ID:**	MVReplaceMan_Mech
**Variable:**	Mitral Valve Replacement: Manufacturer Mechanical
**Definition:**	Please indicate the mitral valve replacement manufacturer.
**Supporting Definition:**	None
**Displayed Value**	Please indicate the mitral valve replacement manufacturer.
**Inclusion Criteria:**	If answered "1= Mechanical" to MVReplaceProsthesis.
**Timing:**	at time of procedure
**Reporting Source:**	Clinical
**Type:**	Single answer
**Value Domain:**	Code
**Response Options:**	1= Medtronic<br>2= Abbot<br>3= Sorin Liva Nova<br>4= On-X<br>888= Other
**Variable ID:**	MVReplaceMedtronicModel_Mech
**Variable:**	Mitral Valve Replacement: Medtronic Mechanical Model
**Definition:**	Please indicate the valve model that was used.
**Supporting Definition:**	Mitral valve replacement: Medtronicmechanical prosthesis
**Displayed Value**	Please indicate the valve model that was used.
**Inclusion Criteria:**	If answered "1= Medtronic" to MVReplaceMan_Mech
**Timing:**	at time of procedure
**Reporting Source:**	Clinical
**Type:**	Single answer
**Value Domain:**	Code
**Response Options:**	1= ATS Open Pivot Aortic Standard<br>2= ATS Open Pivot AP<br>3= ATS Open Pivot AP 360<br>888= Other
**Variable ID:**	MVReplaceAbbotModel_Mech
**Variable:**	Mitral Valve Replacement: Abbot Model Mechanical
**Definition:**	Please indicate the valve model that was used.
**Supporting Definition:**	Mitral valve replacement: Abbot mechanical prosthesis
**Displayed Value**	Please indicate the valve model that was used.
**Inclusion Criteria:**	If answered "2= Abbot" to MVReplaceMan_Mech
**Timing:**	at time of procedure
**Reporting Source:**	Clinical
**Type:**	Single answer
**Value Domain:**	Code
**Response Options:**	1= Regent<br>2= Regent Flexcuff<br>3= Masters<br>4= Masters HP<br>5= Masters Series 15<br>6= Masters MECJ<br>888= Other
**Variable ID:**	MVReplaceSorinLiveNovaModel_Mech
**Variable:**	Mitral Valve Replacement: Sorin Liva Nova Model Mechanical
**Definition:**	Please indicate the valve model that was used.
**Supporting Definition:**	Mitral valve replacement: Sorin Liva Nova mechanical prosthesis

**Displayed Value**	Please indicate the valve model that was used.
**Inclusion Criteria:**	If answered "3= Sorin Liva Nova" to MVReplaceMan_Mech
**Timing:**	at time of procedure
**Reporting Source:**	Clinical
**Type:**	Single answer
**Value Domain:**	Code
**Response Options:**	1= Carbomedics Standard<br>2= Carbomedics Top-Hat<br>3= Carbomedics Reduced<br>4= Carbomedics Pediatric<br>5= Bicarbon Slimline<br>6= Bicarbon Overline<br>7= Bicarbon Fitline<br>8= Carbomedics Standard Mitral<br>9= Carbomedics Optiform<br>10= Bicarbon Fitline Mitral<br>11= Cardio
**Variable ID:**	MVReplaceOnX_Mech
**Variable:**	Mitral Valve Replacement: On-X Mechanical Model
**Definition:**	Please indicate the valve model that was used.
**Supporting Definition:**	Mitral valve replacement: On-X mechanical prosthesis
**Displayed Value**	Please indicate the valve model that was used.
**Inclusion Criteria:**	If answered "4= On-X" to MVReplaceMan_Mech
**Timing:**	at time of procedure
**Reporting Source:**	Clinical
**Type:**	Single answer
**Value Domain:**	Code
**Response Options:**	1= On-X Standard<br>2= On-X Conform X<br>3= On-X Anatomic
**Variable ID:**	MVReplaceOther
**Variable:**	Mitral Valve Replacement:Other
**Definition:**	Please indicate the valve model that was used.
**Supporting Definition:**	Mitral valve replacement: other prosthesis manufacturer
**Displayed Value**	Please indicate the valve model that was used.
**Inclusion Criteria:**	If answered "888= Other" to MVReplaceMan.
**Timing:**	at time of procedure
**Reporting Source:**	Clinical
**Type:**	Free text
**Value Domain:**	String
**Response Options:**	None
**Variable ID:**	MVReplaceMan_Bio
**Variable:**	Mitral Valve Replacement: Manufacturer biological valve
**Definition:**	Please indicate the mitral valve replacement manufacturer.
**Supporting Definition:**	None
**Displayed Value**	Please indicate the mitral valve replacement manufacturer.
**Inclusion Criteria:**	If answered "2= Biological" to MVReplaceProsthesis.
**Timing:**	at time of procedure
**Reporting Source:**	Clinical
**Type:**	Single answer
**Value Domain:**	Code
**Response Options:**	1= Medtronic<br>2= Abbot<br>3= Sorin Liva Nova<br>4= Edwards<br>5= Labcor<br>6= Meril<br>888= Other
**Variable ID:**	MVReplaceMedtronicModel_Bio
**Variable:**	Mitral Valve Replacement: Medtronic Model Biological
**Definition:**	Please indicate the valve model that was used.

**Supporting Definition:**	Mitral valve replacement: Medtronic biological prosthesis
**Displayed Value**	Please indicate the valve model that was used.
**Inclusion Criteria:**	If answered "1= Medtronic" to MVReplaceMan_Bio
**Timing:**	at time of procedure
**Reporting Source:**	Clinical
**Type:**	Single answer
**Value Domain:**	Code
**Response Options:**	1= Mosaïc<br>2= Mosaïc Ultra<br>3= Hancock II<br>4= Hancock II Ultra<br>5= Avalus<br>6= Corevalve<br>7= Corevalve Evolut-R<br>8= Corevalve Evolut Pro<br>888= Other

**Variable ID:**	MVReplaceAbbotModel_Bio
**Variable:**	Mitral Valve Replacement: Abbot Model Biological
**Definition:**	Please indicate the valve model that was used.
**Supporting Definition:**	Mitral valve replacement: Abbot biological prosthesis
**Displayed Value**	Please indicate the valve model that was used.
**Inclusion Criteria:**	If answered "2= Abbot" to MVReplaceMan_Bio
**Timing:**	at time of procedure
**Reporting Source:**	Clinical
**Type:**	Single answer
**Value Domain:**	Code
**Response Options:**	1= Epic<br>2= Epic Supra<br>3= Epic plus supra<br>4= Trifecta<br>5= Trifecta glide<br>6= Biocor<br>7= Biocor supra<br>888= Other

**Variable ID:**	MVReplaceSorinLiveNovaModel_Bio
**Variable:**	Mitral Valve Replacement: Sorin Liva Nova Model Biological
**Definition:**	Please indicate the valve model that was used.
**Supporting Definition:**	Mitral valve replacement: Sorin Liva Nova Biological prosthesis
**Displayed Value**	Please indicate the valve model that was used.
**Inclusion Criteria:**	If answered "3= Sorin Liva Nova" to MVReplaceMan_Bio
**Timing:**	at time of procedure
**Reporting Source:**	Clinical
**Type:**	Single answer
**Value Domain:**	Code
**Response Options:**	1= Crown PRT<br>2= Solo Smart Sutureless<br>3= Perceval sutureless<br>4= Pericarbon more<br>888= Other

**Variable ID:**	MVReplaceEdwardsModel_Bio
**Variable:**	Mitral Valve Replacement: Edwards Model Biological
**Definition:**	Please indicate the valve model that was used.
**Supporting Definition:**	Mitral valve replacement: Edwards Biological prosthesis
**Displayed Value**	Please indicate the valve model that was used.
**Inclusion Criteria:**	If answered "4= Edwards" to MVReplaceMan_Bio
**Timing:**	at time of procedure
**Reporting Source:**	Clinical
**Type:**	Single answer
**Value Domain:**	Code
**Response Options:**	1= Perimount Aortic CEP 2900

	2= Perimount Magna Ease Aortic 3300 TFX<br>3= Inspiris Resilia 11500A<br>4= Perimount Mitral CEP Plus 6900 PC<br>5= Perimount Magna Ease Mitral 7300 TFX<br>888= Other

**Variable ID:**	MVReplaceLabcorModel_Bio
**Variable:**	Mitral Valve Replacement: Labcor Model Biological
**Definition:**	Please indicate the valve model that was used.
**Supporting Definition:**	Mitral valve replacement: Labcor Biological prosthesis
**Displayed Value**	Please indicate the valve model that was used.
**Inclusion Criteria:**	If answered "5= Labcor" to MVReplaceMan_Bio
**Timing:**	at time of procedure
**Reporting Source:**	Clinical
**Type:**	Single answer
**Value Domain:**	Code
**Response Options:**	1= Labcor TLBP<br>2= Dokimos plus<br>888= Other

**Variable ID:**	MVReplaceMericalModel_Bio
**Variable:**	Mitral Valve Replacement: Meril Model Biological
**Definition:**	Please indicate the valve model that was used.
**Supporting Definition:**	Mitral valve replacement:Meril Biological prosthesis
**Displayed Value**	Please indicate the valve model that was used.
**Inclusion Criteria:**	If answered "6= Meril" to MVReplaceMan_Bio
**Timing:**	at time of procedure
**Reporting Source:**	Clinical
**Type:**	Single answer
**Value Domain:**	Code
**Response Options:**	1= Dafodil<br>888= Other

**Variable ID:**	MVReplaceSize
**Variable:**	Mitral Valve Replacement: follow-up question 2
**Definition:**	Please indicate the label size.
**Supporting Definition:**	None
**Displayed Value**	Please indicate the label size.
**Inclusion Criteria:**	If answered "1= MV replacement"to TypeMVsurgery
**Timing:**	at time of procedure
**Reporting Source:**	Clinical
**Type:**	Numerical value
**Value Domain:**	quantity
**Response Options:**	Size

**Variable ID:**	MVrepairType
**Variable:**	Type of mitral valve repair
**Definition:**	Please indicate the type of mitral valve repair
**Supporting Definition:**	None
**Displayed Value**	Please indicate the type of mitral valve repair
**Inclusion Criteria:**	If answered "2= MV repair" to TypeMVsurgery
**Timing:**	at time of procedure
**Reporting Source:**	Clinical
**Type:**	Single answer
**Value Domain:**	Code
**Response Options:**	1= Annuloplasty<br>2= Leaflet repair<br>3= Neochord repair

**Variable ID:**	MVPercutInt_Man
**Variable:**	Mitral Valve percutaneous intervention device: Manufacturer
**Definition:**	Please indicate the device manufacturer for the replacement/repair valve used in the percutaneous intervention.
**Supporting Definition:**	None
**Displayed Value**	Please indicate the device manufacturer for the replacement/repair valve used in the percutaneous intervention.

**Inclusion Criteria:**	If answerd "2= yes, percutanous treatment" to treatment AND "2= Mitral valve" to ValveTreatment.
**Timing:**	at time of procedure
**Reporting Source:**	Clinical
**Type:**	Single answer
**Value Domain:**	Code
**Response Options:**	1= Medtronic<br>2= Abbot<br>3= Boston Scientific<br>4= New Valve Technology<br>5= Edwards<br>888= Other

**Variable ID:**	MVPercutIntModel_Medtronic
**Variable:**	Mitral Valve percutaneous intervention device: Medtronic Model
**Definition:**	Please indicate the valve model that was used.
**Supporting Definition:**	Mitral Valve percutaneous intervention device: Medtronic Model
**Displayed Value**	Please indicate the valve model that was used.
**Inclusion Criteria:**	If answered "1= Medtronic" to MVPercutInt_Man
**Timing:**	at time of procedure
**Reporting Source:**	Clinical
**Type:**	Single answer
**Value Domain:**	Code
**Response Options:**	1= Evolut R<br>2= Evolut Pro<br>888= Other

**Variable ID:**	MVPercutIntModel_Abbot
**Variable:**	Mitral Valve percutaneous intervention device: Abbot Model
**Definition:**	Please indicate the valve model that was used.
**Supporting Definition:**	Mitral Valve percutaneous intervention device: Abbot Model
**Displayed Value**	Please indicate the valve model that was used.
**Inclusion Criteria:**	If answered "2= Abbot" to MVPercutInt_Man
**Timing:**	at time of procedure
**Reporting Source:**	Clinical
**Type:**	Single answer
**Value Domain:**	Code
**Response Options:**	1= Mitraclip<br>2= Triclip<br>3= Portico<br>888= Other

**Variable ID:**	MVPercutIntModel_BostonSci
**Variable:**	Mitral Valve percutaneous intervention device: Boston Scientific Model
**Definition:**	Please indicate the valve model that was used.
**Supporting Definition:**	Mitral Valve percutaneous intervention device: Boston Scientific Model
**Displayed Value**	Please indicate the valve model that was used.
**Inclusion Criteria:**	If answered "3= Boston Scientific" to MVPercutInt_Man
**Timing:**	at time of procedure
**Reporting Source:**	Clinical
**Type:**	Single answer
**Value Domain:**	Code
**Response Options:**	1= Accurate Neo<br>2= Accurate Neo2<br>3= Portico<br>888= Other

**Variable ID:**	MVPercutIntModel_NVT
**Variable:**	Mitral Valve percutaneous intervention device: New Valve Technology Model
**Definition:**	Please indicate the valve model that was used.
**Supporting Definition:**	Mitral Valve percutaneous intervention device: New Valve Technology Model
**Displayed Value**	Please indicate the valve model that was used.
**Inclusion Criteria:**	If answered "4= New Valve Technology" to MVPercutInt_Man
**Timing:**	at time of procedure
**Reporting Source:**	Clinical

**Type:**	Single answer
**Value Domain:**	Code
**Response Options:**	1= Allegra<br>888= Other

**Variable ID:**	MVPercutIntModel_Edwards
**Variable:**	Mitral Valve percutaneous intervention device: Edwards Model
**Definition:**	Please indicate the valve model that was used.
**Supporting Definition:**	Mitral Valve percutaneous intervention device: Edwards Model
**Displayed Value**	Please indicate the valve model that was used.
**Inclusion Criteria:**	If answered "5= Edwards" to MVPercutInt_Man
**Timing:**	at time of procedure
**Reporting Source:**	Clinical
**Type:**	Single answer
**Value Domain:**	Code
**Response Options:**	1= Sapien 3<br>2= Sapien XT<br>888= Other

**Variable ID:**	MVPercutIntSize
**Variable:**	Mitral Valve Percutaneous Intervention Valve Size
**Definition:**	Please indicate the mitral valve replacement/repair label size.
**Supporting Definition:**	None
**Displayed Value**	Please indicate the mitral valve replacement/repair label size.
**Inclusion Criteria:**	If answerd "2= yes, percutanous treatment" to treatment AND "2= Mitral valve" to ValveTreatment.
**Timing:**	at time of procedure
**Reporting Source:**	Clinical
**Type:**	Numerical value
**Value Domain:**	quantity
**Response Options:**	Size in mm

**Variable ID:**	TCIdeviceOtherMV
**Variable:**	Name and manufacturer device mitral valve
**Definition:**	Please indicate the name/manufacturer of the transcatheter MV device if it does not appear in the list provided.
**Supporting Definition:**	None
**Displayed Value**	Please indicate the name/manufacturer of the transcatheter MV device if it does not appear in the list provided.
**Inclusion Criteria:**	if answered "888= Other" to MVPercutInt_Man
**Timing:**	at time of procedure
**Reporting Source:**	Clinical
**Type:**	Free text
**Value Domain:**	String
**Response Options:**	None

TV intervention

**Variable ID:**	TypeTVsurgery
**Variable:**	Type of tricuspid valve surgery
**Definition:**	Please indicate if the TV was repaired or replaced.
**Supporting Definition:**	None
**Displayed Value**	Please indicatie if the MV was repaired or replaced.
**Inclusion Criteria:**	If answered "3= yes, surgical treatment" on Treatment AND "3=Tricuspid valve" on ValveTreatment
**Timing:**	at time of procedure
**Reporting Source:**	Clinical
**Type:**	Single answer
**Value Domain:**	Code
**Response Options:**	1= "TV replacement" 2= "TV repair"

**Variable ID:**	TVReplaceProsthesis
**Variable:**	Tricuspid Valve Replacement: follow-up question 1
**Definition:**	Please indicate the valve prosthesis type.

**Supporting Definition:**	None
**Displayed Value**	Please indicate the valve prosthesis type.
**Inclusion Criteria:**	If answered "1= TV replacement"to TypeTVsurgery
**Timing:**	at time of procedure
**Reporting Source:**	Clinical
**Type:**	Single answer
**Value Domain:**	Code
**Response Options:**	1= Mechanical<br>2= Bioprosthesis<br>3= Homograft<br>888=other

**Variable ID:**	TVReplaceMan_Mech
**Variable:**	Tricuspid Valve Replacement: Manufacturer Mechanical
**Definition:**	Please indicate the tricuspid valve replacement manufacturer.
**Supporting Definition:**	None
**Displayed Value**	Please indicate the tricuspid valve replacement manufacturer.
**Inclusion Criteria:**	If answered "1= Mechanical" to TVReplaceProsthesis.
**Timing:**	at time of procedure
**Reporting Source:**	Clinical
**Type:**	Single answer
**Value Domain:**	Code
**Response Options:**	1= Medtronic<br>2= Abbot<br>3= Sorin Liva Nova<br>4= On-X<br>888= Other

**Variable ID:**	TVReplaceMedtronicModel_Mech
**Variable:**	Tricuspid Valve Replacement: Medtronic Mechanical Model
**Definition:**	Please indicate the valve model that was used.
**Supporting Definition:**	Tricuspid valve replacement: Medtronicmechanical prosthesis
**Displayed Value**	Please indicate the valve model that was used.
**Inclusion Criteria:**	If answered "1= Medtronic" to TVReplaceMan_Mech
**Timing:**	at time of procedure
**Reporting Source:**	Clinical
**Type:**	Single answer
**Value Domain:**	Code
**Response Options:**	1= ATS Open Pivot Aortic Standard<br>2= ATS Open Pivot AP<br>3= ATS Open Pivot AP 360<br>888= Other

**Variable ID:**	TVReplaceAbbotModel_Mech
**Variable:**	Tricuspid Valve Replacement: Abbot Model Mechanical
**Definition:**	Please indicate the valve model that was used.
**Supporting Definition:**	Tricuspid valve replacement: Abbot mechanical prosthesis
**Displayed Value**	Please indicate the valve model that was used.
**Inclusion Criteria:**	If answered "2= Abbot" to TVReplaceMan_Mech
**Timing:**	at time of procedure
**Reporting Source:**	Clinical
**Type:**	Single answer
**Value Domain:**	Code
**Response Options:**	1= Regent<br>2= Regent Flexcuff<br>3= Masters<br>4= Masters HP<br>5= Masters Series 15<br>6= Masters MECJ<br>888= Other

**Variable ID:**	TVReplaceSorinLiveNovaModel_Mech
**Variable:**	Tricuspid Valve Replacement: Sorin Liva Nova Model Mechanical
**Definition:**	Please indicate the valve model that was used.
**Supporting Definition:**	Tricuspid valve replacement: Sorin Liva Nova mechanical prosthesis

**Displayed Value**	Please indicate the valve model that was used.
**Inclusion Criteria:**	If answered "3= Sorin Liva Nova" to TVReplaceMan_Mech
**Timing:**	at time of procedure
**Reporting Source:**	Clinical
**Type:**	Single answer
**Value Domain:**	Code
**Response Options:**	1= Carbomedics Standard<br>2= Carbomedics Top-Hat<br>3= Carbomedics Reduced<br>4= Carbomedics Pediatric<br>5= Bicarbon Slimline<br>6= Bicarbon Overline<br>7= Bicarbon Fitline<br>8= Carbomedics Standard Mitral<br>9= Carbomedics Optiform<br>10= Bicarbon Fitline Mitral<br>11= Cardio

**Variable ID:**	TVReplaceOnX_Mech
**Variable:**	Tricuspid Valve Replacement: On-X Mechanical Model
**Definition:**	Please indicate the valve model that was used.
**Supporting Definition:**	Tricuspid valve replacement: On-X mechanical prosthesis
**Displayed Value**	Please indicate the valve model that was used.
**Inclusion Criteria:**	If answered "4= On-X" to TVReplaceMan_Mech
**Timing:**	at time of procedure
**Reporting Source:**	Clinical
**Type:**	Single answer
**Value Domain:**	Code
**Response Options:**	1= On-X Standard<br>2= On-X Conform X<br>3= On-X Anatomic

**Variable ID:**	TVReplaceOther
**Variable:**	Tricuspid Valve Replacement:Other
**Definition:**	Please indicate the valve model that was used.
**Supporting Definition:**	Tricuspid valve replacement: other prosthesis manufacturer
**Displayed Value**	Please indicate the valve model that was used.
**Inclusion Criteria:**	If answered "888= Other" to TVReplaceMan.
**Timing:**	at time of procedure
**Reporting Source:**	Clinical
**Type:**	Free text
**Value Domain:**	String
**Response Options:**	None

**Variable ID:**	TVReplaceMan_Bio
**Variable:**	Tricuspid Valve Replacement: Manufacturer biological valve
**Definition:**	Please indicate the tricuspid valve replacement manufacturer.
**Supporting Definition:**	None
**Displayed Value**	Please indicate the tricuspid valve replacement manufacturer.
**Inclusion Criteria:**	If answered "2= Biological" to TVReplaceProsthesis.
**Timing:**	at time of procedure
**Reporting Source:**	Clinical
**Type:**	Single answer
**Value Domain:**	Code
**Response Options:**	1= Medtronic<br>2= Abbot<br>3= Sorin Liva Nova<br>4= Edwards<br>5= Labcor<br>6= Meril<br>888= Other

**Variable ID:**	TVReplaceMedtronicModel_Bio
**Variable:**	Tricuspid Valve Replacement: Medtronic Model Biological

**Definition:**	Please indicate the valve model that was used.
**Supporting Definition:**	Tricuspid valve replacement: Medtronic biological prosthesis
**Displayed Value**	Please indicate the valve model that was used.
**Inclusion Criteria:**	If answered "1= Medtronic" to TVReplaceMan_Bio
**Timing:**	at time of procedure
**Reporting Source:**	Clinical
**Type:**	Single answer
**Value Domain:**	Code
**Response Options:**	1= Mosaïc<br>2= Mosaïc Ultra<br>3= Hancock II<br>4= Hancock II Ultra<br>5= Avalus<br>6= Corevalve<br>7= Corevalve Evolut-R<br>8= Corevalve Evolut Pro<br>888= Other
**Variable ID:**	TVReplaceAbbotModel_Bio
**Variable:**	Tricuspid Valve Replacement: Abbot Model Biological
**Definition:**	Please indicate the valve model that was used.
**Supporting Definition:**	Tricuspid valve replacement: Abbot biological prosthesis
**Displayed Value**	Please indicate the valve model that was used.
**Inclusion Criteria:**	If answered "2= Abbot" to TVReplaceMan_Bio
**Timing:**	at time of procedure
**Reporting Source:**	Clinical
**Type:**	Single answer
**Value Domain:**	Code
**Response Options:**	1= Epic<br>2= Epic Supra<br>3= Epic plus supra<br>4= Trifecta<br>5= Trifecta glide<br>6= Biocor<br>7= Biocor supra<br>888= Other
**Variable ID:**	TVReplaceSorinLiveNovaModel_Bio
**Variable:**	Tricuspid Valve Replacement: Sorin Liva Nova Model Biological
**Definition:**	Please indicate the valve model that was used.
**Supporting Definition:**	Tricuspid valve replacement: Sorin Liva Nova Biological prosthesis
**Displayed Value**	Please indicate the valve model that was used.
**Inclusion Criteria:**	If answered "3= Sorin Liva Nova" to TVReplaceMan_Bio
**Timing:**	at time of procedure
**Reporting Source:**	Clinical
**Type:**	Single answer
**Value Domain:**	Code
**Response Options:**	1= Crown PRT<br>2= Solo Smart Sutureless<br>3= Perceval sutureless<br>4= Pericarbon more<br>888= Other
**Variable ID:**	TVReplaceEdwardsModel_Bio
**Variable:**	Tricuspid Valve Replacement: Edwards Model Biological
**Definition:**	Please indicate the valve model that was used.
**Supporting Definition:**	Tricuspid valve replacement: Edwards Biological prosthesis
**Displayed Value**	Please indicate the valve model that was used.
**Inclusion Criteria:**	If answered "4= Edwards" to TVReplaceMan_Bio
**Timing:**	at time of procedure
**Reporting Source:**	Clinical
**Type:**	Single answer
**Value Domain:**	Code

**Response Options:**	1= Perimount Aortic CEP 2900<br>2= Perimount Magna Ease Aortic 3300 TFX<br>3= Inspiris Resilia 11500A<br>4= Perimount Mitral CEP Plus 6900 PC<br>5= Perimount Magna Ease Mitral 7300 TFX<br>888= Other

**Variable ID:**	TVReplaceLabcorModel_Bio
**Variable:**	Tricuspid Valve Replacement: Labcor Model Biological
**Definition:**	Please indicate the valve model that was used.
**Supporting Definition:**	Tricuspid valve replacement: Labcor Biological prosthesis
**Displayed Value**	Please indicate the valve model that was used.
**Inclusion Criteria:**	If answered "5= Labcor" to TVReplaceMan_Bio
**Timing:**	at time of procedure
**Reporting Source:**	Clinical
**Type:**	Single answer
**Value Domain:**	Code
**Response Options:**	1= Labcor TLBP<br>2= Dokimos plus<br>888= Other

**Variable ID:**	TVReplaceMericalModel_Bio
**Variable:**	Tricuspid Valve Replacement: Meril Model Biological
**Definition:**	Please indicate the valve model that was used.
**Supporting Definition:**	Tricuspid valve replacement:Meril Biological prosthesis
**Displayed Value**	Please indicate the valve model that was used.
**Inclusion Criteria:**	If answered "6= Meril" to TVReplaceMan_Bio
**Timing:**	at time of procedure
**Reporting Source:**	Clinical
**Type:**	Single answer
**Value Domain:**	Code
**Response Options:**	1= Dafodil<br>888= Other

**Variable ID:**	TVReplaceOther
**Variable:**	Tricuspid Valve Replacement:Other
**Definition:**	Please indicate the valve model that was used.
**Supporting Definition:**	Tricuspid valve replacement: other prosthesis manufacturer
**Displayed Value**	Please indicate the valve model that was used.
**Inclusion Criteria:**	If answered "888= Other" to TVReplaceMan_Bio
**Timing:**	at time of procedure
**Reporting Source:**	Clinical
**Type:**	Free text
**Value Domain:**	String
**Response Options:**	None

**Variable ID:**	TVReplaceSize
**Variable:**	Tricuspid Valve Replacement: follow-up question 2
**Definition:**	Please indicate the label size.
**Supporting Definition:**	None
**Displayed Value**	Please indicate the label size.
**Inclusion Criteria:**	If answered "1= TV replacement"to TypeTVsurgery
**Timing:**	at time of procedure
**Reporting Source:**	Clinical
**Type:**	Numerical value
**Value Domain:**	quantity
**Response Options:**	Size

**Variable ID:**	TVrepairType
**Variable:**	Type of tricuspid valve repair
**Definition:**	Please indicate the type of tricuspid valve repair
**Supporting Definition:**	None
**Displayed Value**	Please indicate the type of mitral valve repair
**Inclusion Criteria:**	If answered "2= TV repair" to TypeTVsurgery
**Timing:**	at time of procedure
**Reporting Source:**	Clinical

**Type:**	Single answer
**Value Domain:**	Code
**Response Options:**	1= Annuloplasty<br>2= leaflet repair<br>3= Pannus removal

**Variable ID:**	Tvannuloplastydetails
**Variable:**	Details of TV annuloplasty
**Definition:**	Please indicate which matrial was used for the tricuspid valve annuloplasty
**Supporting Definition:**	None
**Displayed Value**	Please indicate which matrial was used for the tricuspid valve annuloplasty
**Inclusion Criteria:**	If answered "1= annuloplasty"at TVrepairType
**Timing:**	at time of procedure
**Reporting Source:**	Clinical
**Type:**	Single answer
**Value Domain:**	
**Response Options:**	TBA

**Variable ID:**	TVAnnuloplastyMaterial
**Variable:**	Tricuspid valve annuloplasty material
**Definition:**	Please indicate which matrial was used for the tricuspid valve annuloplasty
**Supporting Definition:**	None
**Displayed Value**	Please indicate which matrial was used for the tricuspid valve annuloplasty
**Inclusion Criteria:**	If answered "1= Annuloplasty" to TVrepairType
**Timing:**	At time of procedure
**Reporting Source:**	Clinical
**Type:**	Single answer
**Value Domain:**	Code
**Response Options:**	1= Prosthetic ring band<br>2= Pericardium<br>3= Suture<br>888= Other

**Variable ID:**	TVRepairRingMan
**Variable:**	Details ring annuloplasty
**Definition:**	Please indicate the manufacturer if a prosthetic ring/band was used in the annuloplasty.
**Supporting Definition:**	None
**Displayed Value**	Please indicate the manufacturer if a prosthetic ring was used in the annuloplasty.
**Inclusion Criteria:**	If answered "1= Prosthetic ring/band" to TVAnnuloplastyMaterial
**Timing:**	at time of procedure
**Reporting Source:**	Clinical
**Type:**	Single answer
**Value Domain:**	Code
**Response Options:**	1= Medtronic<br>2= Abbot<br>3= Sorin Liva Nova<br>4= Edwards<br>5= Labcor<br>888= Other

**Variable ID:**	TVRepairMedtronicModel
**Variable:**	Tricuspid Valve Repair: Medtronic Model
**Definition:**	Please indicate the valve model that was used.
**Supporting Definition:**	Tricuspid valve repair: Medtronic prosthesis
**Displayed Value**	Please indicate the valve model that was used.
**Inclusion Criteria:**	If answered "1= Medtronic" to TVRepairRingMan
**Timing:**	at time of procedure
**Reporting Source:**	Clinical
**Type:**	Single answer
**Value Domain:**	Code
**Response Options:**	1= Anneau Simplici-T<br>2= Anneau Simulus Ring<br>3= Anneau Simulus Band<br>4= Anneau Simulus Flexible Ring

	5= Anneau Simulus Flexible Band<br>6= Anneau Contour 3D<br>7= Anneau Tri-Ad Adams<br>888= Other
**Variable ID:**	TVRepairAbbotModel
**Variable:**	Tricuspid Valve Repair: Abbot Model
**Definition:**	Please indicate the valve model that was used.
**Supporting Definition:**	Tricuspid valve repair: Abbot prosthesis
**Displayed Value**	Please indicate the valve model that was used.
**Inclusion Criteria:**	If answered "2= Abbot" to TVRepairRingMan
**Timing:**	at time of procedure
**Reporting Source:**	Clinical
**Type:**	Single answer
**Value Domain:**	Code
**Response Options:**	1= Anneau Tailor<br>888= Other
**Variable ID:**	TVRepairSorinLivaNovaModel
**Variable:**	Tricuspid Valve Repair: Sorin Liva Nova Model
**Definition:**	Please indicate the valve model that was used.
**Supporting Definition:**	Tricuspid valve repair: Sorin Liva Nova prosthesis
**Displayed Value**	Please indicate the valve model that was used.
**Inclusion Criteria:**	If answered "3= Sorin Liva Nova" to TVRepairRingMan
**Timing:**	at time of procedure
**Reporting Source:**	Clinical
**Type:**	Single answer
**Value Domain:**	Code
**Response Options:**	1= Anneau Sovering Tricuspid Ring<br>2= Anneau Carbomedics AnnuloFlex<br>3= Sovering Band<br>4= Soverin Mini-band<br>888= Other
**Variable ID:**	TVRepairEdwardsModel
**Variable:**	Tricuspid Valve Repair: Edwards Model
**Definition:**	Please indicate the valve model that was used.
**Supporting Definition:**	Tricuspid valve repair: Edwards prosthesis
**Displayed Value**	Please indicate the valve model that was used.
**Inclusion Criteria:**	If answered "4= Edwards" to TVRepairRingMan
**Timing:**	at time of procedure
**Reporting Source:**	Clinical
**Type:**	Single answer
**Value Domain:**	Code
**Response Options:**	1= Anneau Edwards MC3 Tricuspid 4900T<br>2= Anneau Edwards Physio Tricuspid 6200<br>3= Anneau Cosgrove Tricuspid 4600 GK<br>4= Anneau Cosgrove Tricuspid Duraflo 4625 GL<br>888= Other
**Variable ID:**	TVRepairLabcorModel
**Variable:**	Tricuspid Valve Repair: Labcor Model
**Definition:**	Please indicate the valve model that was used.
**Supporting Definition:**	Tricuspid valve repair: Labcor prosthesis
**Displayed Value**	Please indicate the valve model that was used.
**Inclusion Criteria:**	If answered "5= Labcor" to TVRepairRingMan
**Timing:**	at time of procedure
**Reporting Source:**	Clinical
**Type:**	Single answer
**Value Domain:**	Code
**Response Options:**	1= Anneau Star<br>888= Other
**Variable ID:**	TVRepairSize
**Variable:**	Tricuspid Valve Repair: follow-up question 2
**Definition:**	Please indicate the label size.

**Supporting Definition:**	None
**Displayed Value**	Please indicate the label size.
**Inclusion Criteria:**	If answered "2= TV repair"to TypeTVsurgery
**Timing:**	at time of procedure
**Reporting Source:**	Clinical
**Type:**	Numerical value
**Value Domain:**	quantity
**Response Options:**	Size

**Variable ID:**	TVPercutInt_Man
**Variable:**	Tricuspid Valve percutaneous intervention device: Manufacturer
**Definition:**	Please indicate the device manufacturer for the replacement/repair valve used in the percutaneous intervention.
**Supporting Definition:**	None
**Displayed Value**	Please indicate the device manufacturer for the replacement/repair valve used in the percutaneous intervention.
**Inclusion Criteria:**	If answerd "2= yes, percutanous treatment" to treatment AND "3= Tricuspid valve" to ValveTreatment.
**Timing:**	at time of procedure
**Reporting Source:**	Clinical
**Type:**	Single answer
**Value Domain:**	Code
**Response Options:**	1= Medtronic<br>2= Abbot<br>3= Boston Scientific<br>4= New Valve Technology<br>5= Edwards<br>888= Other

**Variable ID:**	TVPercutIntModel_Medtronic
**Variable:**	Tricuspid Valve percutaneous intervention device: Medtronic Model
**Definition:**	Please indicate the valve model that was used.
**Supporting Definition:**	Tricuspid Valve percutaneous intervention device: Medtronic Model
**Displayed Value**	Please indicate the valve model that was used.
**Inclusion Criteria:**	If answered "1= Medtronic" to TVPercutInt_Man
**Timing:**	at time of procedure
**Reporting Source:**	Clinical
**Type:**	Single answer
**Value Domain:**	Code
**Response Options:**	1= Evolut R<br>2= Evolut Pro<br>888= Other

**Variable ID:**	TVPercutIntModel_Abbot
**Variable:**	Tricuspid Valve percutaneous intervention device: Abbot Model
**Definition:**	Please indicate the valve model that was used.
**Supporting Definition:**	Tricuspid Valve percutaneous intervention device: Abbot Model
**Displayed Value**	Please indicate the valve model that was used.
**Inclusion Criteria:**	If answered "2= Abbot" to TVPercutInt_Man
**Timing:**	at time of procedure
**Reporting Source:**	Clinical
**Type:**	Single answer
**Value Domain:**	Code
**Response Options:**	1= Mitraclip<br>2= Triclip<br>3= Portico<br>888= Other

**Variable ID:**	TVPercutIntModel_BostonSci
**Variable:**	Tricuspid Valve percutaneous intervention device: Boston Scientific Model
**Definition:**	Please indicate the valve model that was used.
**Supporting Definition:**	Tricuspid Valve percutaneous intervention device: Boston Scientific Model
**Displayed Value**	Please indicate the valve model that was used.
**Inclusion Criteria:**	If answered "3= Boston Scientific" to TVPercutInt_Man

**Timing:**	at time of procedure
**Reporting Source:**	Clinical
**Type:**	Single answer
**Value Domain:**	Code
**Response Options:**	1= Accurate Neo<br>2= Accurate Neo2<br>3= Portico<br>888= Other

**Variable ID:**	TVPercutIntModel_NVT
**Variable:**	Tricuspid Valve percutaneous intervention device: New Valve Technology Model
**Definition:**	Please indicate the valve model that was used.
**Supporting Definition:**	Tricuspid Valve percutaneous intervention device: New Valve Technology Model
**Displayed Value**	Please indicate the valve model that was used.
**Inclusion Criteria:**	If answered "4= New Valve Technology" to TVPercutInt_Man
**Timing:**	at time of procedure
**Reporting Source:**	Clinical
**Type:**	Single answer
**Value Domain:**	Code
**Response Options:**	1= Allegra<br>888= Other

**Variable ID:**	TVPercutIntModel_Edwards
**Variable:**	Tricuspid Valve percutaneous intervention device: Edwards Model
**Definition:**	Please indicate the valve model that was used.
**Supporting Definition:**	Tricuspid Valve percutaneous intervention device: Edwards Model
**Displayed Value**	Please indicate the valve model that was used.
**Inclusion Criteria:**	If answered "5= Edwards" to TVPercutInt_Man
**Timing:**	at time of procedure
**Reporting Source:**	Clinical
**Type:**	Single answer
**Value Domain:**	Code
**Response Options:**	1= Sapien 3<br>2= Sapien XT<br>888= Other

**Variable ID:**	TVPercutIntSize
**Variable:**	Tricuspid Valve Percutaneous Intervention Valve Size
**Definition:**	Please indicate the tricuspid valve replacement/repair label size.
**Supporting Definition:**	None
**Displayed Value**	Please indicate the tricuspid valve replacement/repair label size.
**Inclusion Criteria:**	If answerd "2= yes, percutanous treatment" to treatment AND "3= Tricuspid valve" to ValveTreatment.
**Timing:**	at time of procedure
**Reporting Source:**	Clinical
**Type:**	Numerical value
**Value Domain:**	quantity
**Response Options:**	Size in mm

**Variable ID:**	TCIdeviceOtherTV
**Variable:**	Name and manufacturer device tricuspid valve
**Definition:**	Please indicate the name/manufacturer of the transcatheter TV device if they are not in the list provided
**Supporting Definition:**	None
**Displayed Value**	Please indicate the name/manufacturer of the transcatheter TV device if they are not in the list provided
**Inclusion Criteria:**	if answered "888= Other" to TCIdeviceTV
**Timing:**	at time of procedure
**Reporting Source:**	Clinical
**Type:**	Free text
**Value Domain:**	String
**Response Options:**	None

Vital Status

Variable ID: vitalstatus
Variable: vital status
Definition: indicate if the person has deceased regardless of cause
Supporting Definition: None
Displayed Value none
Inclusion Criteria: All patients
Timing: Annually
Reporting Source: Clinical
Type: Single answer
Value Domain: Code
Response Options: 0= No
1= Yes
999= Unknown

Variable ID: deceaseddate
Variable: date of death
Definition: the date of death of the person
Supporting Definition: None
Displayed Value none
Inclusion Criteria: all patients if answered 1 = yes to vitalstatus
Timing: Annually
Reporting Source: Clinical
Type: date by ddmmyyyy
Value Domain: date
Response Options: none

Variable ID: EarlyMort
Variable: Early Mortality
Definition: Mortality within 30 days after a surgical/percutaneous intervention.
Supporting Definition: None
Displayed Value Indicate if the person died within 30 days after a surgical/percutaneous intervention.
Inclusion Criteria: If answered "1=Yes" to AllCauseMort, AND "Yes, percutaneous treatment" OR "Yes, surgical treatment" to Treatment
Timing: Annually
Reporting Source: Clinical
Type: Single answer
Value Domain: Code
Response Options: 0= No
1= Yes
999= Unknown

Variable ID: LateMort
Variable: Late Mortality
Definition: Mortality after 30 days after a surgical/percutaneous intervention.
Supporting Definition: None
Displayed Value Indicate if the person died after 30 days after a surgical/percutaneous intervention.
Inclusion Criteria: If answered "1=Yes" to AllCauseMort, AND "Yes, percutaneous treatment" OR "Yes, surgical treatment" to Treatment
Timing: Annually
Reporting Source: Clinical
Type: Single answer
Value Domain: Code
Response Options: 0= No
1= Yes
999= Unknown

Variable ID: ValveMort
Variable: Valve Related Mortality
Definition: Valve-related mortality is any death caused by structural valve deterioration, nonstructural dysfunction, valve thrombosis, embolism, valve-related bleeding, or prosthetic valve endocarditis; death related to reintervention on the operated valve.
Supporting Definition: None
Displayed Value Indicate whether the death was caused by structural valve deterioration, nonstructural dysfunction, valve thrombosis, embolism, valve-related bleeding, or prosthetic valve endocarditis, sudden unexplained death, or by death related to reintervention on th

**Inclusion Criteria:**	If answered "1= Yes" to AllCauseMort
**Timing:**	Annually
**Reporting Source:**	Clinical
**Type:**	Single answer
**Value Domain:**	Code
**Response Options:**	0= No<br>1= Yes<br>999 = Unknown

Quality of life & mental health

**Variable ID:**	EQ-5D-5L_date
**Variable:**	Date of impact daily life prom
**Definition:**	Please indicate the date the EQ-5D-5L s filled in
**Supporting Definition:**	None
**Displayed Value**	None
**Inclusion Criteria:**	All patient
**Timing:**	Baseline; annually; at procedure; 3 months after procedure
**Reporting Source:**	Patient-reported
**Type:**	date by dd/mm/YYYY
**Value Domain:**	Date
**Response Options:**	DD-MM-YYYY

**Variable ID:**	EQ-5D-5L_1
**Variable:**	EQ-5D-5L Question 1
**Definition:**	Please indicate which statement applies to you in terms of mobility today.
**Supporting Definition:**	Descriptive system for health-related quality of life states in adults.
**Displayed Value**	Please indicate which statement applies to you in terms of mobility today.
**Inclusion Criteria:**	All patients
**Timing:**	Baseline; annually; at procedure; 3 months after procedure
**Reporting Source:**	Patient-reported
**Type:**	Single answer
**Value Domain:**	Code
**Response Options:**	1= I have no problems in walking about<br>2= I have slight problems in walking about<br>3= I have moderate problems in walking about<br>4= I have severe problems in walking about<br>5= I am unable to walk about

**Variable ID:**	EQ-5D-5L_2
**Variable:**	EQ-5D-5L Question 2
**Definition:**	Please indicate which statement applies to you in terms of self-care today.
**Supporting Definition:**	Descriptive system for health-related quality of life states in adults.
**Displayed Value**	Please indicate which statement applies to you in terms of self-care today.
**Inclusion Criteria:**	All patients
**Timing:**	Baseline; annually; at procedure; 3 months after procedure
**Reporting Source:**	Patient-reported
**Type:**	Single answer
**Value Domain:**	Code
**Response Options:**	1= I have no problems washing or dressing myself<br>2= I have slight problems washing or dressing myself<br>3= I have moderate problems washing or dressing myself<br>4= I have severe problems washing or dressing myself<br>5= I am unable to wash or dress myself

**Variable ID:**	EQ-5D-5L_3
**Variable:**	EQ-5D-5L Question 3
**Definition:**	Please indicate which statement applies to you in terms of usual activities today.
**Supporting Definition:**	Descriptive system for health-related quality of life states in adults.
**Displayed Value**	Please indicate which statement applies to you in terms of usual activities today.
**Inclusion Criteria:**	All patients
**Timing:**	Baseline; annually; at procedure; 3 months after procedure
**Reporting Source:**	Patient-reported
**Type:**	Single answer

**Value Domain:**	Code
**Response Options:**	1= I have no problems doing my usual activities<br>2= I have slight problems doing my usual activities<br>3= I have moderate problems doing my usual activities<br>4= I have severe problems doing my usual activities<br>5= I am unable to do my usual activities

**Variable ID:**	EQ-5D-5L_4
**Variable:**	EQ-5D-5L Question 4
**Definition:**	Please indicate which statement applies to you in terms of pain/discomfort today.
**Supporting Definition:**	Descriptive system for health-related quality of life states in adults.
**Displayed Value**	Please indicate which statement applies to you in terms of pain/discomfort today.
**Inclusion Criteria:**	All patients
**Timing:**	Baseline; annually; at procedure; 3 months after procedure
**Reporting Source:**	Patient-reported
**Type:**	Single answer
**Value Domain:**	Code
**Response Options:**	1= I have no pain or discomfort<br>2= I have slight pain or discomfort<br>3= I have moderate pain or discomfort<br>4= I have severe pain or discomfort<br>5= I have extreme pain or discomfort

**Variable ID:**	EQ-5D-5L_5
**Variable:**	EQ-5D-5L Question 5
**Definition:**	Please indicate which statement applies to you in terms of anxiety/depression today.
**Supporting Definition:**	Descriptive system for health-related quality of life states in adults.
**Displayed Value**	Please indicate which statement applies to you in terms of anxiety/depression today.
**Inclusion Criteria:**	All patients
**Timing:**	Baseline; annually; at procedure; 3 months after procedure
**Reporting Source:**	Patient-reported
**Type:**	Single answer
**Value Domain:**	Code
**Response Options:**	1= I am not anxious or depressed<br>2= I am slightly anxious or depressed<br>3= I am moderately anxious or depressed<br>4= I am severely anxious or depressed<br>5= I am extremely anxious or depressed

Impact on mental health and daily activities

**Variable ID:**	Impact_HVD_date
**Variable:**	Date of impact daily life prom
**Definition:**	Please indicate the date the HVD impact daily life was filled in
**Supporting Definition:**	None
**Displayed Value**	None
**Inclusion Criteria:**	All patient
**Timing:**	Baseline; annually; at procedure; 3 months after procedure
**Reporting Source:**	Patient-reported
**Type:**	date by dd/mm/YYYY
**Value Domain:**	Date
**Response Options:**	DD-MM-YYYY

**Variable ID:**	Impact_HVD_daily_life_Q1_A
**Variable:**	Impact on Mental Health and Daily Activities Question 1
**Definition:**	To what extent does HVD impact your daily life, including anxiety due to the disease and dependence upon other people because of it. Please indicate how much the following statement applies to your situation:
**Supporting Definition:**	None
**Displayed Value**	My heart condition made me dependent on other people.
**Inclusion Criteria:**	All patients
**Timing:**	Baseline; annually; at procedure; 3 months after procedure
**Reporting Source:**	Patient-reported
**Type:**	Single answer

**Value Domain:**	Code
**Response Options:**	1= Strongly disagree<br>2= Disagree<br>3= Neither agree nor disagree<br>4= Agree<br>5= Strongly agree
**Variable ID:**	Impact_HVD_daily_life_Q2_A
**Variable:**	Impact on Mental Health and Daily Activities Question 2
**Definition:**	To what extent does HVD impact your daily life, including anxiety due to the disease and dependence upon other people because of it. Please indicate how much the following statement applies to you:
**Supporting Definition:**	None
**Displayed Value**	My heart condition damaged my ability to work as before.
**Inclusion Criteria:**	All patients
**Timing:**	Baseline; annually; at procedure; 3 months after procedure
**Reporting Source:**	Patient-reported
**Type:**	Single answer
**Value Domain:**	Code
**Response Options:**	1= Strongly disagree<br>2= Disagree<br>3= Neither agree nor disagree<br>4= Agree<br>5= Strongly agree
**Variable ID:**	Impact_HVD_daily_life_Q3_A
**Variable:**	Impact on Mental Health and Daily Activities Question 3
**Definition:**	To what extent does HVD impact your daily life, including anxiety due to the disease and dependence upon other people because of it. Please indicate how much the following statement applies to you:
**Supporting Definition:**	None
**Displayed Value**	Today, I become more irritated and anxious because of my heart condition.
**Inclusion Criteria:**	All patients
**Timing:**	Baseline; annually; at procedure; 3 months after procedure
**Reporting Source:**	Patient-reported
**Type:**	Single answer
**Value Domain:**	Code
**Response Options:**	1= Strongly disagree<br>2= Disagree<br>3= Neither agree nor disagree<br>4= Agree<br>5= Strongly agree
**Variable ID:**	Impact_HVD_daily_life_Q4_A
**Variable:**	Impact on Mental Health and Daily Activities Question 4
**Definition:**	To what extent does HVD impact your daily life, including anxiety due to the disease and dependence upon other people because of it. Please indicate how much the following statement applies to you:
**Supporting Definition:**	None
**Displayed Value**	Since I developed my heart condition, I feel very upset.
**Inclusion Criteria:**	All patients
**Timing:**	Baseline; annually; at procedure; 3 months after procedure
**Reporting Source:**	Patient-reported
**Type:**	Single answer
**Value Domain:**	Code
**Response Options:**	1= Strongly disagree<br>2= Disagree<br>3= Neither agree nor disagree<br>4= Agree<br>5= Strongly agree
**Variable ID:**	Impact_HVD_daily_life_Q5_A
**Variable:**	Impact on Mental Health and Daily Activities Question 5

**Definition:**	To what extent does HVD impact your daily life, including anxiety due to the disease and dependence upon other people because of it. Please indicate how much the following statement applies to you:
**Supporting Definition:**	None
**Displayed Value**	After I developed my heart condition, I started to get scared that something would happen to me.
**Inclusion Criteria:**	All patients
**Timing:**	Baseline; annually; at procedure; 3 months after procedure
**Reporting Source:**	Patient-reported
**Type:**	Single answer
**Value Domain:**	Code
**Response Options:**	1= Strongly disagree<br>2= Disagree<br>3= Neither agree nor disagree<br>4= Agree<br>5= Strongly agree
**Variable ID:**	Impact_HVD_daily_life_Q6_A
**Variable:**	Impact on Mental Health and Daily Activities Question 6
**Definition:**	To what extent does HVD impact your daily life, including anxiety due to the disease and dependence upon other people because of it. Please indicate how much the following statement applies to you:
**Supporting Definition:**	None
**Displayed Value**	Having a heart condition worries me.
**Inclusion Criteria:**	All patients
**Timing:**	Baseline; annually; at procedure; 3 months after procedure
**Reporting Source:**	Patient-reported
**Type:**	Single answer
**Value Domain:**	Code
**Response Options:**	1= Strongly disagree<br>2= Disagree<br>3= Neither agree nor disagree<br>4= Agree<br>5= Strongly agree
**Variable ID:**	Impact_HVD_daily_life_Q7_A
**Variable:**	Impact on Mental Health and Daily Activities Question 7
**Definition:**	To what extent does HVD impact your daily life, including anxiety due to the disease and dependence upon other people because of it. Please indicate how much the following statement applies to you:
**Supporting Definition:**	None
**Displayed Value**	Because of my heart condition, I have difficulty sleeping.
**Inclusion Criteria:**	All patients
**Timing:**	Baseline; annually; at procedure; 3 months after procedure
**Reporting Source:**	Patient-reported
**Type:**	Single answer
**Value Domain:**	Code
**Response Options:**	1= Strongly disagree<br>2= Disagree<br>3= Neither agree nor disagree<br>4= Agree<br>5= Strongly agree
**Variable ID:**	Impact_HVD_daily_life_Q8_A
**Variable:**	Impact on Mental Health and Daily Activities Question 8
**Definition:**	To what extent does HVD impact your daily life, including anxiety due to the disease and dependence upon other people because of it. Please indicate how much the following statement applies to you:
**Supporting Definition:**	None
**Displayed Value**	Because of my heart condition, I have a lot of difficulty performing activities of daily living.
**Inclusion Criteria:**	All patients
**Timing:**	Baseline; annually; at procedure; 3 months after procedure
**Reporting Source:**	Patient-reported
**Type:**	Single answer

**Value Domain:**	Code
**Response Options:**	1= Strongly disagree<br>2= Disagree<br>3= Neither agree nor disagree<br>4= Agree<br>5= Strongly agree
**Variable ID:**	Impact_HVD_daily_life_Q9_A
**Variable:**	Impact on Mental Health and Daily Activities Question 9
**Definition:**	To what extent does HVD impact your daily life, including anxiety due to the disease and dependence upon other people because of it. Please indicate how much the following statement applies to you:
**Supporting Definition:**	None
**Displayed Value**	Because of my heart condition, I have a lot of shortness of breath.
**Inclusion Criteria:**	All patients
**Timing:**	Baseline; annually; at procedure; 3 months after procedure
**Reporting Source:**	Patient-reported
**Type:**	Single answer
**Value Domain:**	Code
**Response Options:**	1= Strongly disagree<br>2= Disagree<br>3= Neither agree nor disagree<br>4= Agree<br>5= Strongly agree
**Variable ID:**	Impact_HVD_daily_life_Q10_A
**Variable:**	Impact on Mental Health and Daily Activities Question 10
**Definition:**	To what extent does HVD impact your daily life, including anxiety due to the disease and dependence upon other people because of it. Please indicate how much the following statement applies to you:
**Supporting Definition:**	None
**Displayed Value**	Because of my heart condition, I feel very tired.
**Inclusion Criteria:**	All patients
**Timing:**	Baseline; annually; at procedure; 3 months after procedure
**Reporting Source:**	Patient-reported
**Type:**	Single answer
**Value Domain:**	Code
**Response Options:**	1= Strongly disagree<br>2= Disagree<br>3= Neither agree nor disagree<br>4= Agree<br>5= Strongly agree
**Variable ID:**	Impact_HVD_daily_life_Q11_A
**Variable:**	Impact on Mental Health and Daily Activities Question 11
**Definition:**	To what extent does HVD impact your daily life, including anxiety due to the disease and dependence upon other people because of it. Please indicate how much the following statement applies to you:
**Supporting Definition:**	None
**Displayed Value**	Because of my heart condition, I have periods of dizziness.
**Inclusion Criteria:**	All patients
**Timing:**	Baseline; annually; at procedure; 3 months after procedure
**Reporting Source:**	Patient-reported
**Type:**	Single answer
**Value Domain:**	Code
**Response Options:**	1= Strongly disagree<br>2= Disagree<br>3= Neither agree nor disagree<br>4= Agree<br>5= Strongly agree
**Variable ID:**	Impact_HVD_daily_life_Q12_A
**Variable:**	Impact on Mental Health and Daily Activities Question 12

**Definition:**	To what extent does HVD impact your daily life, including anxiety due to the disease and dependence upon other people because of it. Please indicate how much the following statement applies to you:
**Supporting Definition:**	None
**Displayed Value**	After I developed my heart condition, I started to pay more attention to my health.
**Inclusion Criteria:**	All patients
**Timing:**	Baseline; annually; at procedure; 3 months after procedure
**Reporting Source:**	Patient-reported
**Type:**	Single answer
**Value Domain:**	Code
**Response Options:**	1= Strongly disagree<br>2= Disagree<br>3= Neither agree nor disagree<br>4= Agree<br>5= Strongly agree
**Variable ID:**	Impact_HVD_daily_life_Q13_A
**Variable:**	Impact on Mental Health and Daily Activities Question 13
**Definition:**	To what extent does HVD impact your daily life, including anxiety due to the disease and dependence upon other people because of it. Please indicate how much the following statement applies to you:
**Supporting Definition:**	None
**Displayed Value**	I feel okay about my heart condition.
**Inclusion Criteria:**	All patients
**Timing:**	Baseline; annually; at procedure; 3 months after procedure
**Reporting Source:**	Patient-reported
**Type:**	Single answer
**Value Domain:**	Code
**Response Options:**	1= Strongly disagree<br>2= Disagree<br>3= Neither agree nor disagree<br>4= Agree<br>5= Strongly agree
**Variable ID:**	Impact_HVD_daily_life_Q14_A
**Variable:**	Impact on Mental Health and Daily Activities Question 14
**Definition:**	To what extent does HVD impact your daily life, including anxiety due to the disease and dependence upon other people because of it. Please indicate how much the following statement applies to you:
**Supporting Definition:**	None
**Displayed Value**	My sex life stayed the same as before my heart problem.
**Inclusion Criteria:**	All patients
**Timing:**	Baseline; annually; at procedure; 3 months after procedure
**Reporting Source:**	Patient-reported
**Type:**	Single answer
**Value Domain:**	Code
**Response Options:**	1= Strongly disagree<br>2= Disagree<br>3= Neither agree nor disagree<br>4= Agree<br>5= Strongly agree
**Variable ID:**	Impact_HVD_daily_life_Q1_B
**Variable:**	Impact on Mental Health and Daily Activities Question 1
**Definition:**	To what extent does HVD impact your daily life, including anxiety due to the disease and dependence upon other people because of it. Please indicate how you preceive the consequence of the following statement:
**Supporting Definition:**	None
**Displayed Value**	My heart condition made me dependent on other people.
**Inclusion Criteria:**	All patients
**Timing:**	Baseline; annually; at procedure; 3 months after procedure
**Reporting Source:**	Patient-reported
**Type:**	Single answer

**Value Domain:**	Code
**Response Options:**	1= Very bad<br>2= Bad<br>3= Neither bad nor good<br>4= Good<br>5= Very good
**Variable ID:**	Impact_HVD_daily_life_Q2_B
**Variable:**	Impact on Mental Health and Daily Activities Question 2
**Definition:**	To what extent does HVD impact your daily life, including anxiety due to the disease and dependence upon other people because of it. Please indicate how you preceive the consequence of the following statement:
**Supporting Definition:**	None
**Displayed Value**	My heart condition damaged my ability to work as before.
**Inclusion Criteria:**	All patients
**Timing:**	Baseline; annually; at procedure; 3 months after procedure
**Reporting Source:**	Patient-reported
**Type:**	Single answer
**Value Domain:**	Code
**Response Options:**	1= Very bad<br>2= Bad<br>3= Neither bad nor good<br>4= Good<br>5= Very good
**Variable ID:**	Impact_HVD_daily_life_Q3_B
**Variable:**	Impact on Mental Health and Daily Activities Question 3
**Definition:**	To what extent does HVD impact your daily life, including anxiety due to the disease and dependence upon other people because of it. Please indicate how you preceive the consequence of the following statement:
**Supporting Definition:**	None
**Displayed Value**	Today, I become more irritated and anxious because of my heart condition.
**Inclusion Criteria:**	All patients
**Timing:**	Baseline; annually; at procedure; 3 months after procedure
**Reporting Source:**	Patient-reported
**Type:**	Single answer
**Value Domain:**	Code
**Response Options:**	1= Very bad<br>2= Bad<br>3= Neither bad nor good<br>4= Good<br>5= Very good
**Variable ID:**	Impact_HVD_daily_life_Q4_B
**Variable:**	Impact on Mental Health and Daily Activities Question 4
**Definition:**	To what extent does HVD impact your daily life, including anxiety due to the disease and dependence upon other people because of it. Please indicate how you preceive the consequence of the following statement:
**Supporting Definition:**	None
**Displayed Value**	Since I developed my heart condition, I feel very upset.
**Inclusion Criteria:**	All patients
**Timing:**	Baseline; annually; at procedure; 3 months after procedure
**Reporting Source:**	Patient-reported
**Type:**	Single answer
**Value Domain:**	Code
**Response Options:**	1= Very bad<br>2= Bad<br>3= Neither bad nor good<br>4= Good<br>5= Very good
**Variable ID:**	Impact_HVD_daily_life_Q5_B
**Variable:**	Impact on Mental Health and Daily Activities Question 5

**Definition:**	To what extent does HVD impact your daily life, including anxiety due to the disease and dependence upon other people because of it. Please indicate how you preceive the consequence of the following statement:
**Supporting Definition:**	None
**Displayed Value**	After I developed my heart condition, I started to get scared that something would happen to me.
**Inclusion Criteria:**	All patients
**Timing:**	Baseline; annually; at procedure; 3 months after procedure
**Reporting Source:**	Patient-reported
**Type:**	Single answer
**Value Domain:**	Code
**Response Options:**	1= Very bad<br>2= Bad<br>3= Neither bad nor good<br>4= Good<br>5= Very good
**Variable ID:**	Impact_HVD_daily_life_Q6_B
**Variable:**	Impact on Mental Health and Daily Activities Question 6
**Definition:**	To what extent does HVD impact your daily life, including anxiety due to the disease and dependence upon other people because of it. Please indicate how you preceive the consequence of the following statement:
**Supporting Definition:**	None
**Displayed Value**	Having a heart condition worries me.
**Inclusion Criteria:**	All patients
**Timing:**	Baseline; annually; at procedure; 3 months after procedure
**Reporting Source:**	Patient-reported
**Type:**	Single answer
**Value Domain:**	Code
**Response Options:**	1= Very bad<br>2= Bad<br>3= Neither bad nor good<br>4= Good<br>5= Very good
**Variable ID:**	Impact_HVD_daily_life_Q7_B
**Variable:**	Impact on Mental Health and Daily Activities Question 7
**Definition:**	To what extent does HVD impact your daily life, including anxiety due to the disease and dependence upon other people because of it. Please indicate how you preceive the consequence of the following statement:
**Supporting Definition:**	None
**Displayed Value**	Because of my heart condition, I have difficulty sleeping.
**Inclusion Criteria:**	All patients
**Timing:**	Baseline; annually; at procedure; 3 months after procedure
**Reporting Source:**	Patient-reported
**Type:**	Single answer
**Value Domain:**	Code
**Response Options:**	1= Very bad<br>2= Bad<br>3= Neither bad nor good<br>4= Good<br>5= Very good
**Variable ID:**	Impact_HVD_daily_life_Q8_B
**Variable:**	Impact on Mental Health and Daily Activities Question 8
**Definition:**	To what extent does HVD impact your daily life, including anxiety due to the disease and dependence upon other people because of it. Please indicate how you preceive the consequence of the following statement:
**Supporting Definition:**	None
**Displayed Value**	Because of my heart condition, I have a lot of difficulty performing activities of daily living.
**Inclusion Criteria:**	All patients
**Timing:**	Baseline; annually; at procedure; 3 months after procedure
**Reporting Source:**	Patient-reported
**Type:**	Single answer

**Value Domain:**	Code
**Response Options:**	1= Very bad<br>2= Bad<br>3= Neither bad nor good<br>4= Good<br>5= Very good
**Variable ID:**	Impact_HVD_daily_life_Q9_B
**Variable:**	Impact on Mental Health and Daily Activities Question 9
**Definition:**	To what extent does HVD impact your daily life, including anxiety due to the disease and dependence upon other people because of it. Please indicate how you preceive the consequence of the following statement:
**Supporting Definition:**	None
**Displayed Value**	Because of my heart condition, I have a lot of shortness of breath.
**Inclusion Criteria:**	All patients
**Timing:**	Baseline; annually; at procedure; 3 months after procedure
**Reporting Source:**	Patient-reported
**Type:**	Single answer
**Value Domain:**	Code
**Response Options:**	1= Very bad<br>2= Bad<br>3= Neither bad nor good<br>4= Good<br>5= Very good
**Variable ID:**	Impact_HVD_daily_life_Q10_B
**Variable:**	Impact on Mental Health and Daily Activities Question 10
**Definition:**	To what extent does HVD impact your daily life, including anxiety due to the disease and dependence upon other people because of it. Please indicate how you preceive the consequence of the following statement:
**Supporting Definition:**	None
**Displayed Value**	Because of my heart condition, I feel very tired.
**Inclusion Criteria:**	All patients
**Timing:**	Baseline; annually; at procedure; 3 months after procedure
**Reporting Source:**	Patient-reported
**Type:**	Single answer
**Value Domain:**	Code
**Response Options:**	1= Very bad<br>2= Bad<br>3= Neither bad nor good<br>4= Good<br>5= Very good
**Variable ID:**	Impact_HVD_daily_life_Q11_B
**Variable:**	Impact on Mental Health and Daily Activities Question 11
**Definition:**	To what extent does HVD impact your daily life, including anxiety due to the disease and dependence upon other people because of it. Please indicate how you preceive the consequence of the following statement:
**Supporting Definition:**	None
**Displayed Value**	Because of my heart condition, I have periods of dizziness.
**Inclusion Criteria:**	All patients
**Timing:**	Baseline; annually; at procedure; 3 months after procedure
**Reporting Source:**	Patient-reported
**Type:**	Single answer
**Value Domain:**	Code
**Response Options:**	1= Very bad<br>2= Bad<br>3= Neither bad nor good<br>4= Good<br>5= Very good
**Variable ID:**	Impact_HVD_daily_life_Q12_B
**Variable:**	Impact on Mental Health and Daily Activities Question 12

**Definition:**	To what extent does HVD impact your daily life, including anxiety due to the disease and dependence upon other people because of it. Please indicate how you preceive the consequence of the following statement:
**Supporting Definition:**	None
**Displayed Value**	After I developed my heart condition, I started to pay more attention to my health.
**Inclusion Criteria:**	All patients
**Timing:**	Baseline; annually; at procedure; 3 months after procedure
**Reporting Source:**	Patient-reported
**Type:**	Single answer
**Value Domain:**	Code
**Response Options:**	1= Very bad<br>2= Bad<br>3= Neither bad nor good<br>4= Good<br>5= Very good

**Variable ID:**	Impact_HVD_daily_life_Q13_B
**Variable:**	Impact on Mental Health and Daily Activities Question 13
**Definition:**	To what extent does HVD impact your daily life, including anxiety due to the disease and dependence upon other people because of it. Please indicate how you preceive the consequence of the following statement:
**Supporting Definition:**	None
**Displayed Value**	I feel okay about my heart condition.
**Inclusion Criteria:**	All patients
**Timing:**	Baseline; annually; at procedure; 3 months after procedure
**Reporting Source:**	Patient-reported
**Type:**	Single answer
**Value Domain:**	Code
**Response Options:**	1= Very bad<br>2= Bad<br>3= Neither bad nor good<br>4= Good<br>5= Very good

**Variable ID:**	Impact_HVD_daily_life_Q14_B
**Variable:**	Impact on Mental Health and Daily Activities Question 14
**Definition:**	To what extent does HVD impact your daily life, including anxiety due to the disease and dependence upon other people because of it. Please indicate how much the following statement applies to you:
**Supporting Definition:**	None
**Displayed Value**	My sex life stayed the same as before my heart problem.
**Inclusion Criteria:**	All patients
**Timing:**	Baseline; annually; at procedure; 3 months after procedure
**Reporting Source:**	Patient-reported
**Type:**	Single answer
**Value Domain:**	Code
**Response Options:**	1= Very bad<br>2= Bad<br>3= Neither bad nor good<br>4= Good<br>5= Very good

Hospitalization for heart failure

**Variable ID:**	HxHeartFailure
**Variable:**	Hospitalisation for heart failure
**Definition:**	Unplanned hospital admission for clinical treatment for heart failure
**Supporting Definition:**	None
**Displayed Value**	Please indicate if there was an unplanned hospital admission for clinical treatment for heart failure.
**Inclusion Criteria:**	All patients
**Timing:**	Annually

**Reporting Source:**	Clinical
**Type:**	Single answer
**Value Domain:**	Code
**Response Options:**	0= No<br>1= Yes<br>999= Unknown

**Variable ID:**	HxHeartFailure1
**Variable:**	Hospitalisation for heart failure: follow-up 1
**Definition:**	Please indicate the date of the unplanned hospital admission.
**Supporting Definition:**	None
**Displayed Value**	Please indicate the date of the unplanned hospital admission.
**Inclusion Criteria:**	If answered "1= Yes" in HxHeartFailure.
**Timing:**	Annually
**Reporting Source:**	Clinical
**Type:**	Date by DD/MM/YYYY
**Value Domain:**	date
**Response Options:**	DD-MM-YYYY

Valve Dysfunction

**Variable ID:**	DateValvemeasurments
**Variable:**	Valve measurement date
**Definition:**	Please indicate the date of the valve measurements for the aortic valve, mitral valve, and tricuspid valve.
**Supporting Definition:**	None
**Displayed Value**	Please indicate the date of the valve measurements for the aortic valve, mitral valve, and tricuspid valve.
**Inclusion Criteria:**	All patients
**Timing:**	At discharge, 3 months, and 6 months after intervention; then annually
**Reporting Source:**	Clinical
**Type:**	Date by DD/MM/YYYY
**Value Domain:**	date
**Response Options:**	DD-MM-YYYY

**Variable ID:**	Avregurg_fup
**Variable:**	Aortic Valve Regurgitation
**Definition:**	Please indicate the grade of aortic valve regurgitation.
**Supporting Definition:**	Grade 0 to 4 according to Table in supplement
**Displayed Value**	Please indicate the grade of aortic valve regurgitation.
**Inclusion Criteria:**	All patients
**Timing:**	At discharge, 3 months, and 6 months after intervention; then annually
**Reporting Source:**	Clinical
**Type:**	Single answer
**Value Domain:**	Code
**Response Options:**	0 = None<br>1= Grade 1<br>2= Grade 2<br>3= Grade 3<br>4= Grade 4

**Variable ID:**	Mvregurg_fup
**Variable:**	Mitral Valve Regurgitation
**Definition:**	Please indicate the grade of mitral valve regurgitation.
**Supporting Definition:**	Grade 0 to 4 according to Table in supplement
**Displayed Value**	Please indicate the grade of mitral valve regurgitation.
**Inclusion Criteria:**	All patients
**Timing:**	At discharge, 3 months, and 6 months after intervention; then annually
**Reporting Source:**	Clinical
**Type:**	Single answer
**Value Domain:**	Code
**Response Options:**	0 = None<br>1= Grade 1

	2= Grade 2<br>3= Grade 3<br>4= Grade 4
**Variable ID:**	Tvregurg_fup
**Variable:**	Tricuspid Valve Regurgitation
**Definition:**	Please indicate the grade of tricuspid valve regurgitation.
**Supporting Definition:**	Grade 0 to 3 according to Table in supplement
**Displayed Value**	Please indicate the grade of tricuspid valve regurgitation.
**Inclusion Criteria:**	All patients
**Timing:**	At discharge, 3 months, and 6 months after intervention; then annually
**Reporting Source:**	Clinical
**Type:**	Single answer
**Value Domain:**	Code
**Response Options:**	0 = None<br>1= Grade 1<br>2= Grade 2<br>3= Grade 3
**Variable ID:**	AoValveStenosisAVA_fup
**Variable:**	Aortic Valve stenosis: Valve area in (cm2)
**Definition:**	Please indicate the valve area (in cm2) of the aortic valve.
**Supporting Definition:**	None
**Displayed Value**	Please indicate the valve area (in cm2) of the aortic valve.
**Inclusion Criteria:**	All patients
**Timing:**	At discharge, 3 months, and 6 months after intervention; then annually
**Reporting Source:**	Clinical
**Type:**	Numerical value
**Value Domain:**	quantity
**Response Options:**	Area in cm2
**Variable ID:**	AoValveStenosisGradient_fup
**Variable:**	Aortic Valve stenosis: Maximum transvalvular gradient (mm/Hg)
**Definition:**	Please indicate the mean transvalvular gradient (mm/Hg) of the aortic valve.
**Supporting Definition:**	None
**Displayed Value**	Please indicate the mean transvalvular gradient (mm/Hg) of the aortic valve.
**Inclusion Criteria:**	All patients
**Timing:**	At discharge, 3 months, and 6 months after intervention; then annually
**Reporting Source:**	Clinical
**Type:**	Numerical value
**Value Domain:**	quantity
**Response Options:**	Gradient (mm/Hg)
**Variable ID:**	AoValveStenosisVelocity_fup
**Variable:**	Aortic Valve stenosis: valve velocity (cm/s)
**Definition:**	Please indicate the maximum valve velocity (cm/s) of the aortic valve.
**Supporting Definition:**	None
**Displayed Value**	Please indicate the maximum valve velocity (cm/s) of the aortic valve.
**Inclusion Criteria:**	All patients
**Timing:**	At discharge, 3 months, and 6 months after intervention; then annually
**Reporting Source:**	Clinical
**Type:**	Numerical value
**Value Domain:**	quantity
**Response Options:**	Velocity (cm/s)
**Variable ID:**	MVStenosisarea_fup
**Variable:**	Mitral Valve stenosis: Valve area in (cm2)
**Definition:**	Please indicate the valve area (in cm2) of the mitral valve.
**Supporting Definition:**	None
**Displayed Value**	Please indicate the valve area (in cm2) of the mitral valve.
**Inclusion Criteria:**	All patients
**Timing:**	At discharge, 3 months, and 6 months after intervention; then annually
**Reporting Source:**	Clinical
**Type:**	Numerical value
**Value Domain:**	quantity
**Response Options:**	Area in cm2

**Variable ID:**	MVStenosisgradient_fup
**Variable:**	Mitral Valve stenosis: Mean transvalvular gradient (mm/Hg)
**Definition:**	Please indicate the mean transvalvular gradient (mm/Hg) of the mitral valve.
**Supporting Definition:**	None
**Displayed Value**	Please indicate the mean transvalvular gradient (mm/Hg) of the mitral valve.
**Inclusion Criteria:**	All patients
**Timing:**	At discharge, 3 months, and 6 months after intervention; then annually
**Reporting Source:**	Clinical
**Type:**	Numerical value
**Value Domain:**	quantity
**Response Options:**	Gradient (mm/Hg)

**Variable ID:**	MVStenosisVelocity_fup
**Variable:**	Mitral Valve stenosis: valve velocity (cm/s)
**Definition:**	Please indicate the valve velocity (cm/s) of the mitral valve.
**Supporting Definition:**	None
**Displayed Value**	Please indicate the valve velocity (cm/s) of the mitral valve.
**Inclusion Criteria:**	All patients
**Timing:**	At discharge, 3 months, and 6 months after intervention; then annually
**Reporting Source:**	Clinical
**Type:**	Numerical value
**Value Domain:**	quantity
**Response Options:**	Velocity (cm/s)

**Variable ID:**	TVStenosis_fup
**Variable:**	Tricuspid Valve Stenosis: mean transvalvular gradient (mm/Hg)
**Definition:**	Please indicate the mean transvalvular gradient (mm/Hg) of the tricuspid valve.
**Supporting Definition:**	None
**Displayed Value**	Please indicate the mean transvalvular gradient (mm/Hg) of the tricuspid valve.
**Inclusion Criteria:**	All patients
**Timing:**	At discharge, 3 months, and 6 months after intervention; then annually
**Reporting Source:**	Clinical
**Type:**	Numerical value
**Value Domain:**	quantity
**Response Options:**	Gradient (mm/Hg)

Cardiac Fitness

**Variable ID:**	AnginaPectoris
**Variable:**	Angina Pectoris: follow-up 1
**Definition:**	Functional classification according to the Canadian Cardiovascular Society.
**Supporting Definition:**	0 Asymptomatic Angina Mild myocardial ischemia with no symptoms.<br>I Angina only with strenuous exertion Presence of angina during strenuous, rapid, or prolonged ordinary activity (walking or climbing the stairs).<br>II: Angina with moderate exertion Slight limitation of ordinary activities when they are performed rapidly, after meals, in cold, in wind, under emotional stress, during the first few hours after waking up, but also walking uphill, climbing more than one flight of ordinary stairs at a normal pace and in normal conditions.<br>III Angina with mild exertion Having difficulties walking one or two blocks or climbing one flight of stairs at normal pace and conditions.<br>IV Angina at rest No exertion needed to trigger angina.
**Displayed Value**	Please indicate which classification the patient falls under regarding Angina Pectoris.
**Inclusion Criteria:**	All patients
**Timing:**	Annually (if 0)<br>6 months (if >0)
**Reporting Source:**	Clinical
**Type:**	Single answer
**Value Domain:**	Code
**Response Options:**	0 = "No Angina"<br>1 = "Class I"<br>2 = "Class II"<br>3 = "Class III"<br>4 = "Class IV"

**Variable ID:**	AnginaPectorisDate
**Variable:**	Angina Pectoris: follow-up 2
**Definition:**	Please indicate the date of this evaluation.
**Supporting Definition:**	None
**Displayed Value**	Please indicate the date of this evaluation.
**Inclusion Criteria:**	All patients
**Timing:**	At discharge, 3 months, and 6 months after intervention; then annually
**Reporting Source:**	Clinical
**Type:**	Date by DD/MM/YYYY
**Value Domain:**	date
**Response Options:**	DD-MM-YYYY

**Variable ID:**	nyha_fup
**Variable:**	nyha follow up
**Definition:**	patient symptoms
**Supporting Definition:**	nyha - new york heart association classification of heart failure functional capacity
**Displayed Value**	None
**Inclusion Criteria:**	All patients
**Timing:**	At discharge, 3 months, and 6 months after intervention; then annually
**Reporting Source:**	Clinical
**Type:**	Single answer
**Value Domain:**	Code
**Response Options:**	0 = Class I: No limitation of physical activity. Ordinary physical activity does not cause undue fatigue, palpitation, dyspnea (shortness of breath).<br>1 = Class II: Slight limitation of physical activity. Comfortable at rest. Ordinary physical activity res

**Variable ID:**	nyhaDate
**Variable:**	nyha data
**Definition:**	Please indicate the date of this evaluation.
**Supporting Definition:**	None
**Displayed Value**	Please indicate the date of this evaluation.
**Inclusion Criteria:**	All patients
**Timing:**	At discharge, 3 months, and 6 months after intervention; then annually
**Reporting Source:**	Clinical
**Type:**	Date by DD/MM/YYYY
**Value Domain:**	date
**Response Options:**	DD-MM-YYYY

LVF

**Variable ID:**	LVFdate
**Variable:**	Date Left ventricular measurement date
**Definition:**	Please indicate the date of LVF measurement
**Supporting Definition:**	None
**Displayed Value**	Please indicate the date of LVF measurement
**Inclusion Criteria:**	All patients
**Timing:**	At discharge, 3 months, and 6 months after intervention; then annually
**Reporting Source:**	Clinical
**Type:**	Date by DD/MM/YYYY
**Value Domain:**	date
**Response Options:**	DD-MM-YYYY

**Variable ID:**	LVF
**Variable:**	LVF: Left Ventricular Ejection Fraction
**Definition:**	Left ventricular ejection fraction (Please state range of patient’s ejection fraction)
**Supporting Definition:**	None
**Displayed Value**	Please indicate the range of the patient's left ventricular ejection fraction.
**Inclusion Criteria:**	All patients
**Timing:**	At discharge, 3 months, and 6 months after intervention; then annually
**Reporting Source:**	Clinical
**Type:**	Numerical value
**Value Domain:**	quantity
**Response Options:**	1 - 100

Rhytm

**Variable ID:**	BaselineRhythmDate
**Variable:**	Baseline rhythm date
**Definition:**	Please indicate the date the baseline rhythm has been measured.
**Supporting Definition:**	None
**Displayed Value**	Please indicate whether baseline rhythm has been measured.
**Inclusion Criteria:**	All patients
**Timing:**	Prior to and post procedure, 3 and 6 months after discharge; then annually
**Reporting Source:**	Clinical
**Type:**	Date by DD/MM/YYYY
**Value Domain:**	date
**Response Options:**	DD-MM-YYYY
**Variable ID:**	BaselineRhythm1
**Variable:**	Baseline Rhythm: follow-up question 1
**Definition:**	Baseline rhythm of the patient at time of measurement. If a patient has a permanent/temporary pacemaker, but has AF/sinus on electrocardiogram, AF/sinus should be coded.
**Supporting Definition:**	None
**Displayed Value**	Please indicate the baseline rhythm of the patients at time of measurement. If a patient has a permanent/temporary pacemaker, but has AF/sinus on electrocardiogram, AF/sinus should be coded.
**Inclusion Criteria:**	All patients
**Timing:**	Prior to and post procedure, 3 and 6 months after discharge; then annually
**Reporting Source:**	Clinical
**Type:**	Single answer
**Value Domain:**	Code
**Response Options:**	1 = Sinus<br>2 = Atrial fibrillation/flutter<br>3 = Paced<br>888 = Other

Bleeding

**Variable ID:**	BleedingEvent
**Variable:**	Bleeding event
**Definition:**	Type 1<br>Overt bleeding that does require medical intervention by a healthcare professional, leading to hospitalization, an increased level of care, or medical evaluation<br>Overt bleeding that requires a transfusion of 1 unit of whole blood/red blood cells
**Supporting Definition:**	Adjusted definition from VARC 3. Ref: Généreux P, Piazza N, et al. Valve Academic Research Consortium 3: Updated Endpoint Definitions for Aortic Valve Clinical Research. J Am Coll Cardiol. 2021 Jun, 77 (21) 2717–2746. https://doi.org/10.1016/j.jacc.2021.02.038
**Displayed Value**	Please indicate if the patient had a bleeding event according to the definition.
**Inclusion Criteria:**	All patients
**Timing:**	Index event
**Reporting Source:**	Clinical
**Type:**	Single answer
**Value Domain:**	Code
**Response Options:**	0 = No<br>1 = Yes, type 1<br>2 = Yes, type 2<br>3 = Yes, type 3<br>4 = Yes, type 4 definite<br>5 = Yes, type 4, probably
**Variable ID:**	BleedingEventDate
**Variable:**	Bleeding event date
**Definition:**	Please indicate the date of this event.

Supporting Definition: None
Displayed Value Please indicate the date of this event.
Inclusion Criteria: If answered to BleedingEvent.
Timing: Annually
Reporting Source: Clinical
Type: Date by DD/MM/YYYY
Value Domain: date
Response Options: DD-MM-YYYY

Endocarditis

Variable ID: Endocarditis
Variable: Endocarditis
Definition: Please indicate whether the patient has been diagnosed with endocarditis.
Supporting Definition: Diagnosis modified Duke criteria and 2015 modified diagnostic criteria.
Displayed Value Has the patient been diagnosed with endocarditis?
Inclusion Criteria: All patients
Timing: Annually
Reporting Source: Clinical
Type: Single answer
Value Domain: Code
Response Options: 0= No
1= Yes
999= Unknown

Variable ID: EndocarditisDate
Variable: Endocarditis: follow up question 1
Definition: Please indicate the date of this diagnosis
Supporting Definition: None
Displayed Value Please indicate the date of this diagnosis
Inclusion Criteria: If answered to "1=Yes" to Endocarditis.
Timing: Annually
Reporting Source: Clinical
Type: Date by DD/MM/YYYY
Value Domain: date
Response Options: DD-MM-YYYY

Valve Thrombosis

Variable ID: ValveThromb
Variable: Valve Thrombosis
Definition: Valve thrombosis is any thrombus not caused by infection attached to or near an operated valve that occludes part of the blood flow path, interferes with valve function, or is sufficiently large to warrant treatment.
Supporting Definition: None
Displayed Value Has the patient been diagnosed with valve thrombosis?
Inclusion Criteria: All patients
Timing: Annually
Reporting Source: Clinical
Type: Single answer
Value Domain: Code
Response Options: 0 = No
1 = Yes
999= Unknown

Variable ID: ValveThrombDate
Variable: Valve Thrombosis: follow up question 1
Definition: Please indicate the date of this event
Supporting Definition: None
Displayed Value Please indicate the date of this event.
Inclusion Criteria: If answered to "1=Yes" to ValveThromb.
Timing: Annually
Reporting Source: Clinical
Type: Date by DD/MM/YYYY

**Value Domain:**	date
**Response Options:**	DD-MM-YYYY

Structural and Non- Structural Valve Dysfunction

**Variable ID:**	SVD
**Variable:**	Structural Valve Deterioration
**Definition:**	Is the stenosis or regurgitation due to structural deterioration?
**Supporting Definition:**	Only after intervention: The term structural valve deterioration refers to changes intrinsic to the valve, such as wear, fracture, poppet escape, calcification, leaflet tear, stent creep, and suture line disruption of components of a prosthetic valve; it also refers to new chordal rupture, leaflet disruption, or leaflet retraction of a repaired valve."
**Displayed Value**	Is the stenosis or regurgitation due to structural deterioration?
**Inclusion Criteria:**	Patients that underwent valve replacement
**Timing:**	At discharge, 3 months, and 6 months after intervention; then annually
**Reporting Source:**	Clinical
**Type:**	Single answer
**Value Domain:**	Code
**Response Options:**	0 = Not applicable, no stenosis/regurgitation<br>1 = No<br>2 = Yes<br>999 = Unknown

**Variable ID:**	NSVD
**Variable:**	Non-structural valve deterioration
**Definition:**	Is the stenosis or regurgitation due to non-structural deterioration?
**Supporting Definition:**	The term nonstructural dysfunction refers to problems (exclusive of thrombosis and infection) that do not directly involve valve components yet result in dysfunction of an operated valve, as diagnosed by reoperation, autopsy, or clinical investigation. Examples of nonstructural dysfunction include the following: entrapment by pannus, tissue, or suture paravalvular leak valvular leak; inappropriate sizing or positioning; residual leak or obstruction after valve implantation or repair; and clinically important intravascular hemolytic anemia. In addition, nonstructural dysfunction includes development of aortic or pulmonic regurgitation as a result of technical errors, dilatation of the sinotubular junction, or dilatation of the valve annulus after either valve replacement with stentless prostheses (eg, pulmonary autograft, aortic allograft, and xenograft valves) or aortic valve sparing operations if the cusps are seen to be normal at reoperation, autopsy, or clinical investigation. For percutaneous and transapical approaches to aortic valve replacement or conventional open aortic valve replacement, new onset of coronary ischemia from coronary ostial obstruction or paravalvular aortic regurgitation is considered non-structural dysfunction"
**Displayed Value**	Is the stenosis or regurgitation due to non-structural deterioration?
**Inclusion Criteria:**	Patients that underwent valve replacement
**Timing:**	At discharge, 3 months, and 6 months after intervention; then annually
**Reporting Source:**	Clinical
**Type:**	Single answer
**Value Domain:**	Code
**Response Options:**	0 = Not applicable, no stenosis/regurgitation<br>1 = No<br>2 = Yes, paravalvular<br>3 = Yes, but not paravalvular<br>999 = Unknown

**Variable ID:**	DeviceMigration
**Variable:**	Device migration
**Definition:**	Has a device migration been identified after percutaneous intervention?
**Supporting Definition:**	After initial correct positioning, the valve prosthesis moves upwards or downwards, within the aortic annulus from its initial position, with or without consequences (only applicable to percutaneous devices)
**Displayed Value**	Has a device migration been identified after percutaneous intervention?
**Inclusion Criteria:**	All patients
**Timing:**	At discharge, 3 months, and 6 months after intervention; then annually
**Reporting Source:**	Clinical
**Type:**	Single answer

Value Domain: Code
Response Options: 0= No
1= Yes
999= Unknown

Stroke and thromboembolic event

Variable ID: stroketype
Variable: stroke type
Definition: Please indicate if the patient had a stroke and indicatie the type
Supporting Definition: Acute episode of a focal or global neurological deficit with at least one of the following: change in the level of consciousness, hemiplegia, hemiparesis, numbness, or sensory loss affecting one side of the body, dysphasia or aphasia, hemianopia, amaurosis fugax, or other neurological signs or symptoms consistent with stroke
Stroke: duration of a focal or global neurological deficit ≥24 h; OR <24 h if available neuroimaging documents a new haemorrhage or infarct; OR the neurological deficit results in death Stroke classification
Ischaemic: an acute episode of focal cerebral, spinal, or retinal dysfunction caused by infarction of the central nervous system tissue
Haemorrhagic: an acute episode of focal or global cerebral or spinal dysfunction caused by intraparenchymal, intraventricular, or subarachnoid haemorrhage
A stroke may be classified as undetermined if there is insufficient information to allow categorization as ischaemic or haemorrhagic. Ref:Généreux P, Piazza N, et al. Valve Academic Research Consortium 3: Updated Endpoint Definitions for Aortic Valve Clinical Research. J Am Coll Cardiol. 2021 Jun, 77 (21) 2717–2746. https://doi.org/10.1016/j.jacc.2021.02.038
Displayed Value Indicate stroke type
Inclusion Criteria: All patients
Timing: Annually
Reporting Source: Clinical
Type: Single answer
Value Domain: Code
Response Options: 1=Ischemic stroke (IS)
2=Intracerebral hemorrhage (ICH)
3=Transient ischemic attack (TIA)
999=Stroke of unknown type

Variable ID: strokedate
Variable: Stroke date
Definition: Indicate the date of this event
Supporting Definition: None
Displayed Value Indicate the date of this event
Inclusion Criteria: All patients
Timing: Annually
Reporting Source: Clinical
Type: Date by DD/MM/YYYY
Value Domain: date
Response Options: DD-MM-YYYY

Variable ID: ThromboEvent
Variable: Thromboembolic Event (non-cerebral)
Definition: Has the patient had a non-cerebral thromboembolic event?
Supporting Definition: A non-cerebral embolic event is an embolus documented operatively, at autopsy or clinically that produces signs or symptoms attributable to complete or partial obstruction of a non-cerebral artery.
Displayed Value Has the patient had a non-cerebral thromboembolic event?
Inclusion Criteria: All patients
Timing: Annually
Reporting Source: Clinical
Type: Single answer
Value Domain: Code
Response Options: 0= No
1= Yes

	999= Unknown

**Variable ID:**	ThromboEvent1
**Variable:**	Thromboembolic Event (non-cerebral): follow-up question 1
**Definition:**	Please indicate the date of the event.
**Supporting Definition:**	None
**Displayed Value**	Please indicate the date of the event.
**Inclusion Criteria:**	If answered "1=Yes" to ThromboEvent
**Timing:**	Annually
**Reporting Source:**	Clinical
**Type:**	Date by DD/MM/YYYY
**Value Domain:**	date
**Response Options:**	DD-MM-YYYY

Re-intervention

**Variable ID:**	Re-intervention
**Variable:**	Re-intervention
**Definition:**	Please indicate if a patient is undergoing a reintervention after a previous valve intervention.
**Supporting Definition:**	Please refer to case-mix variables for details of previous intervention.
**Displayed Value**	Please indicate if a patient is undergoing a reintervention after a previous valve intervention.
**Inclusion Criteria:**	All patients who have undergone a heart valve procedure
**Timing:**	at time of procedure
**Reporting Source:**	Clinical
**Type:**	Single answer
**Value Domain:**	Code
**Response Options:**	0 = No<br>1 = Yes, surgical<br>2= Yes, percutaneous

**Variable ID:**	Re-intervention1
**Variable:**	Re-intervention: follow-up question 1
**Definition:**	Please specify which valve was involved in the re-intervention procedure.
**Supporting Definition:**	None
**Displayed Value**	Please specify which valve was involved in the re-intervention procedure.
**Inclusion Criteria:**	If answered "1=Yes" to Re-intervention
**Timing:**	at time of procedure
**Reporting Source:**	Clinical
**Type:**	Multiple answer
**Value Domain:**	Code
**Response Options:**	1= Aortic valve<br>2 = Mitral valve<br>3 = Tricuspid valve

**Variable ID:**	Re-intervention2
**Variable:**	Re-intervention: follow-up question 2
**Definition:**	Please specify when this re-intervention took place.
**Supporting Definition:**	None
**Displayed Value**	Please specify when this re-intervention took place.
**Inclusion Criteria:**	If answered "1=Yes" to Re-intervention
**Timing:**	at time of procedure
**Reporting Source:**	Clinical
**Type:**	Date by DD/MM/YYYY
**Value Domain:**	date
**Response Options:**	DD-MM-YYYY

Operative Complications

**Variable ID:**	ConvHeartQx
**Variable:**	Conversion to open-heart surgery
**Definition:**	Please indicate if there was need for a conversion to open heart surgery during percutaneous/minimally invasive interventions.
**Supporting Definition:**	None

**Displayed Value**	Please indicate if there was need for a conversion to open heart surgery during percutaneous/minimally invasive interventions.
**Inclusion Criteria:**	All patients who are undergoing minimally invasive heart valve procedure.
**Timing:**	at time of procedure
**Reporting Source:**	Clinical
**Type:**	Single answer
**Value Domain:**	Code
**Response Options:**	0= No<br>1= Yes<br>999= Unknown

**Variable ID:**	ReopBleeding
**Variable:**	Reoperation for bleeding
**Definition:**	Please indicate if there has ever been need for reoperation due to bleeding.
**Supporting Definition:**	In case of surgical intervention:<br>Return to the operating room for re-thoracotomy/sternotomy.<br>In case of percutaneous intervention:<br>Unplanned intervention for the purpose of controlling the bleeding5.
**Displayed Value**	Please indicate if there has ever been need for reoperation due to bleeding.
**Inclusion Criteria:**	All patients who have undergone a valve intervention (surgical/percutaneous)
**Timing:**	Within index hospitalisation after intervention
**Reporting Source:**	Clinical
**Type:**	Single answer
**Value Domain:**	Code
**Response Options:**	0= No<br>1= Yes<br>999= Unknown

**Variable ID:**	PeriProceduralMI
**Variable:**	Periprocedural myocardial Infarction
**Definition:**	Please indicate if the patient had periprocedural myocardial infarction.
**Supporting Definition:**	Criteria for Cabg-Related MI ≤48 Hours After the Index Procedure (Type 5 MI)<br>CABG-related MI is arbitrarily defined as elevation of cTn values >10 times the 99th percentile URL in patients with normal baseline cTn values. In patients with elevated preprocedure cTn in whom cTn levels are stable (≤20% variation) or falling, the postprocedure cTn must rise by >20%. However, the absolute postprocedural value still must be >10 times the 99th percentile URL. In addition, 1 of the following elements is required:<br>Development of new pathological Q waves*;<br>Angiographic documented new graft occlusion or new native coronary artery occlusion;<br>Imaging evidence of new loss of viable myocardium or new regional wall motion abnormality in a pattern consistent with an ischemic etiology.<br>*Isolated development of new pathological Q waves meets the type 5 MI criteria if cTn values are elevated and rising but <10 times the 99th percentile URL.
**Displayed Value**	Please indicate if the patient had a perioperative myocardial infarction.
**Inclusion Criteria:**	All patients who have undergone a valve intervention (surgical/percutaneous)
**Timing:**	Within index hospitalisation after intervention
**Reporting Source:**	Clinical
**Type:**	Single answer
**Value Domain:**	Code
**Response Options:**	0= No<br>1= Yes<br>999= Unknown

**Variable ID:**	NewPermPacemaker
**Variable:**	New permanent pacemaker
**Definition:**	Please indicate whether the patient has undergone a pacemaker implantation (repeat implant is not necessary to capture).
**Supporting Definition:**	None
**Displayed Value**	Please indicate whether the patient has undergone a pacemaker implantation (repeat implant is not necessary to capture).
**Inclusion Criteria:**	All patients
**Timing:**	Annually

**Reporting Source:**	Clinical
**Type:**	Single answer
**Value Domain:**	Code
**Response Options:**	0= No<br>1= Yes<br>999= Unknown

**Variable ID:**	NewPermPacemaker1
**Variable:**	New permanent pacemaker: follow-up question 1
**Definition:**	Please indicate the date of this event
**Supporting Definition:**	None
**Displayed Value**	Please indicate the date of this event
**Inclusion Criteria:**	If answered "1=Yes" to NewPermPacemaker
**Timing:**	At event occurence (annual check)
**Reporting Source:**	Clinical
**Type:**	Date by DD/MM/YYYY
**Value Domain:**	date
**Response Options:**	DD-MM-YYYY

**Variable ID:**	Vascular complication
**Variable:**	Major/minor vascular complications
**Definition:**	Please indicate if the patient had major or minor vascular complications after a percutanous intervention.
**Supporting Definition:**	Any complication related to the device insertion, delivery, and complete removal of all its components (delivery catheter, sheath, guide wire), excluding the actual implantation in the heart. Categrorized in<br>Major<br>One of the following:<br>1. Aortic dissection or aortic rupture<br>Vascular (arterial or venous) injury (perforation, rupture, dissection, stenosis, ischaemia, arterial or venous thrombosis including pulmonary embolism, arteriovenous fistula, pseudoaneurysm, haematoma, retroperitoneal haematoma, infection) or compartment syndrome resulting in death, type 2 bleeding, limb or visceral ischaemia, or irreversible neurologic impairment<br>2. Distal embolization (non-cerebral) from a vascular source resulting in death, amputation, limb or visceral ischaemia, or irreversible end-organ damage.<br>Unplanned endovascular or surgical intervention resulting in death, type 2 bleeding, limb or visceral ischaemia, or irreversible neurologic impairment<br>3. Closure device failure resulting in death, type 2 bleeding, limb or visceral ischaemia, or irreversible neurologic impairment<br>Minor<br>One of the following:<br>1. Vascular (arterial or venous) injury (perforation, rupture, dissection, stenosis, ischaemia, arterial or venous thrombosis including pulmonary embolism, arteriovenous fistula, pseudoaneurysm, haematoma, retroperitoneal haematoma, infection) not resulting in death, type 2 bleeding, limb or visceral ischaemia, or irreversible neurologic impairment<br>2. Distal embolization treated with embolectomy and/or thrombectomy, not resulting in death, amputation, limb or visceral ischaemia, or irreversible endorgan damage<br>3. Any unplanned endovascular or surgical intervention, ultra-sound guided compression, or thrombin injection, not resulting in death, type 2 bleeding, limb or visceral ischaemia, or irreversible neurologic impairment<br>4. Closure device failure not resulting in death, type 2 bleeding, limb or visceral ischaemia, or irreversible neurologic impairment (ref:
**Displayed Value**	Please indicate if the patient had major or minor vascular complications after a percutanous intervention.
**Inclusion Criteria:**	All patients who have undergone a valve intervention (surgical/percutaneous)
**Timing:**	Within index hospitalisation after percuteanous intervention
**Reporting Source:**	Clinical
**Type:**	Single answer
**Value Domain:**	Code

**Response Options:**	0 = No<br>1 = Yes, major<br>2 = Yes, minor
**Variable ID:**	LCOS
**Variable:**	Low cardiac output syndrome
**Definition:**	Please indicate if the patient had low cardiac output syndrome after the intervention.
**Supporting Definition:**	1. Need for mechanical circulatory support with IABP, LVAD, or extracorporeal membrane oxygenation during surgery or within 5 postoperative days, and/or<br>2. Hemodynamic instability requiring continued pharmacologic support with ≥2 inotropic medications (epinephrine, milrinone dobutamine dopamine) on postoperative day 1<br>Reference: Duncan AE, Kartashov A, Robinson SB, Randall D, Zhang K, Luber J, et al. Risk factors, resource use, and cost of postoperative low cardiac output syndrome. The Journal of Thoracic and Cardiovascular Surgery. 2022;163(5):1890-8.e10
**Displayed Value**	Please indicate if the patient had low cardiac output syndrome after the intervention.
**Inclusion Criteria:**	After valve intervention (surgical/percutaneous)
**Timing:**	Within 5 days post intervention
**Reporting Source:**	Clinical
**Type:**	Single answer
**Value Domain:**	Code
**Response Options:**	0= No<br>1= Yes<br>999= Unknown
**Variable ID:**	HospStay
**Variable:**	Hospital Stay
**Definition:**	Please indicate the number of days spent in the index hospital after valve intervention.
**Supporting Definition:**	None
**Displayed Value**	Please indicate the number of days spent in the index hospital after valve intervention.
**Inclusion Criteria:**	All patients who have undergone a valve intervention (surgical/percutaneous).
**Timing:**	Within index hospitalisation after percutaneous intervention
**Reporting Source:**	Clinical
**Type:**	Numerical value
**Value Domain:**	quantity
**Response Options:**	Number of days
**Variable ID:**	ICU_Stay
**Variable:**	ICU Stay
**Definition:**	Please indicate the number of days spent in the ICU after valve intervention.
**Supporting Definition:**	None
**Displayed Value**	Please indicate the number of days spent in the ICU after valve intervention.
**Inclusion Criteria:**	All patients who have undergone a valve intervention (surgical/percutaneous)
**Timing:**	Within index hospitalisation after percutaneous intervention
**Reporting Source:**	Clinical
**Type:**	Numerical value
**Value Domain:**	quantity
**Response Options:**	Number of days
**Variable ID:**	DichargedTo
**Variable:**	Discharged to
**Definition:**	Please indicate where the patient is discharged to.
**Supporting Definition:**	None
**Displayed Value**	Please indicate where the patient is discharged to.
**Inclusion Criteria:**	All patients who have undergone a valve intervention (surgical/percutaneous)
**Timing:**	Within index hospitalisation after percutaneous intervention
**Reporting Source:**	Clinical
**Type:**	Single answer
**Value Domain:**	Code
**Response Options:**	1 = Home or community dwelling (not home hospice) 2 = Residential facility 3 = Dedicated inpatient rehabilitation facility 4 = Another acute care hospital 5 = Patient died in hospital 888 = Other 999 = Unknown

Note: This version has been corrected to include the response options for the EQ-5D-5L questionnaire (variables EQ-5D-5L_1, EQ-5D-5L_2, EQ-5D-5L_3, EQ-5D-5L_4 and EQ-5D-5L_5).

ICHOM Contact Information

**Website**	http://www.ichom.org
**Business Address**	**United States Office**<br>399 Boylston Street 6th Floor<br>Boston, MA 02116, USA
**Email**	info@ICHOM.org

Reference Guide Revisions

Reference Guide Version	Location within Reference Guide	Content Change
5.0.1	Whole Document	Updates were made to harmonize the Heart Valve Disease Set with other ICHOM Cardiometabolic Sets. The following changes were made:<br><br>Gender_CVD variable was added.

www.ichom.org
