# Supplementary material for: The First International Consortium for Health Outcomes Measurement (ICHOM) Standard Dataset for Reporting Outcomes in Heart Valve Disease: Moving From Device- to Patient-Centered Outcomes
Source: Innovations (Phila). 2025 Feb 19;20(2):133–47. doi: 10.1177/15569845241269309 (PMC12089684; doi:10.1177/15569845241269309)
Supplement: sj-pdf-3-inv-10.1177_15569845241269309 – Supplemental material for The First International Consortium for Health Outcomes Measurement (ICHOM) Standard Dataset for Reporting Outcomes in Heart Valve Disease: Moving From Device- to Patient-Centered Outcomes [file sj-pdf-3-inv-10.1177_15569845241269309.pdf]

Supplementary Text 1

Search terms for the clinical outcome measures and case-mix in Embase.

('heart valve surgery'/mj/de OR 'heart valve prosthesis'/mj/de OR 'aortic valve prosthesis'/mj/exp OR 'heart valve bioprosthesis'/mj/exp OR 'mechanical heart valve'/mj/exp OR 'mitral valve prosthesis'/mj/exp OR 'percutaneous heart valve'/mj/exp OR 'tricuspid valve prosthesis'/mj/exp OR 'mitral valve surgery'/mj/de OR valvuloplasty/mj/de OR 'annuloplasty'/mj/exp OR 'aortic valve repair'/mj/exp OR 'heart valve commissurotomy'/mj/exp OR 'mitral valve repair'/mj/exp OR 'transluminal valvuloplasty'/mj/exp OR 'tricuspid valve repair'/mj/exp OR 'heart valve replacement'/mj/de OR 'aortic valve replacement'/mj/exp OR 'mitral valve replacement'/mj/exp OR 'Ross procedure'/mj/exp OR 'tricuspid valve replacement'/mj/exp OR 'valvular heart disease'/mj/de OR 'aortic valve disease'/mj/exp OR 'blood regurgitation'/mj/exp OR 'chorda tendinea rupture'/mj/exp OR 'heart murmur'/mj/exp OR 'heart valve prolapse'/mj/exp OR 'heart valve regurgitation'/mj/exp OR 'heart valve stenosis'/mj/exp OR 'mitral valve disease'/mj/exp OR 'paravalvular leak'/mj/exp OR 'prosthetic valve dysfunction'/mj/exp OR 'prosthetic valve endocarditis'/mj/exp OR 'prosthetic valve thrombosis'/mj/exp OR 'tricuspid valve disease'/mj/exp OR (((heart* OR aort* OR mitral* OR tricuspid*) NEAR/3 (valve OR valvular*) NEAR/6 (replacement* OR prosthe* OR surger* OR disease* OR endocarditi* OR thrombo* OR rupture* OR regurgitate* OR stenosis OR implant* OR repair* OR dysfunction*)) OR ((aort* OR mitral* OR tricuspid*) NEAR/3 valvuloplast*)):ti) NOT [conference abstract]/lim AND [english]/lim NOT ('case report'/de OR case-report*:ti) AND ('treatment outcome'/exp OR (outcome*):ab,ti)

© 2024 The Authors. This is an Open Access article under the terms of the Creative Commons Attribution Non-Commercial-NoDerivs License, which permits use, distribution, and reproduction in any medium, provided that the original work is properly cited, the use is noncommercial, and no modifications or adaptations are made.

Supplementary Text 2

Search terms for the patient-reported outcome measures in Embase, MEDLINE, Web of Science, and Cochrane.

Embase.com 781

('heart valve surgery'/de OR 'heart valve prosthesis'/de OR 'aortic valve prosthesis'/exp OR 'heart valve bioprosthesis'/exp OR 'mechanical heart valve'/exp OR 'mitral valve prosthesis'/exp OR 'percutaneous heart valve'/exp OR 'tricuspid valve prosthesis'/exp OR 'mitral valve surgery'/de OR valvuloplasty/de OR 'annuloplasty'/exp OR 'aortic valve repair'/exp OR 'heart valve commissurotomy'/exp OR 'mitral valve repair'/exp OR 'transluminal valvuloplasty'/exp OR 'tricuspid valve repair'/exp OR 'heart valve replacement'/de OR 'aortic valve replacement'/exp OR 'mitral valve replacement'/exp OR 'Ross procedure'/exp OR 'tricuspid valve replacement'/exp OR 'valvular heart disease'/de OR 'aortic valve disease'/exp OR 'blood regurgitation'/exp OR 'chorda tendinea rupture'/exp OR 'heart murmur'/exp OR 'heart valve prolapse'/exp OR 'heart valve regurgitation'/exp OR 'heart valve stenosis'/exp OR 'mitral valve disease'/exp OR 'paravalvular leak'/exp OR 'prosthetic valve dysfunction'/exp OR 'prosthetic valve endocarditis'/exp OR 'prosthetic valve thrombosis'/exp OR 'tricuspid valve disease'/exp OR (((heart* OR aort* OR mitral* OR tricuspid*) NEAR/3 (valve OR valvular*) NEAR/6 (replacement* OR prosthe* OR surger* OR disease* OR endocarditi* OR thrombo* OR rupture* OR regurgitate* OR stenosis OR implant* OR repair* OR dysfunction*)) OR ((aort* OR mitral* OR tricuspid*) NEAR/3 valvuloplast*)):ab,ti) NOT [conference abstract]/lim AND [english]/lim NOT ('case report'/de OR case-report*:ti) AND ('patient-reported outcome'/de OR ('quality of life'/exp AND (questionnaire/de OR 'self report'/de)) OR 'quality of life assessment'/exp OR (((patient*-report* OR patient*-perceiv* OR patient*-percept* OR self*-report* OR selfreport*) NEAR/10 (outcome* OR function* OR symptom* OR quality-of-life OR qol OR hrqol OR health-status OR improvement* OR disabilit* OR problem* OR result* OR impact*)) OR ((quality-of-life OR qol OR hrqol) NEAR/3 (tool* OR measur* OR questionnaire*))):ab,ti)

Medline Ovid 313

(Heart Valve Prosthesis Implantation/ OR Heart Valve Prosthesis/ OR Mitral Valve Annuloplasty/ OR Transcatheter Aortic Valve Replacement/ OR Heart Valve Diseases/ OR Aortic Valve Disease/ OR Heart Murmurs/ OR exp Heart Valve Prolapse/ OR (((heart* OR aort* OR mitral* OR tricuspid*) ADJ3 (valve OR valvular*) ADJ6 (replacement* OR prosthe* OR surger* OR disease* OR endocarditi* OR thrombo* OR rupture* OR regurgitate* OR stenosis OR implant* OR repair* OR dysfunction*)) OR ((aort* OR mitral* OR tricuspid*) ADJ3 valvuloplast*)).ab,ti.) AND english.la. NOT (case reports/ OR case-report*.ti.) AND (Patient Reported Outcome Measures/ OR (Quality of Life/ AND ("Surveys and Questionnaires"/ OR Self Report/)) OR quality of life assessment/ OR (((patient*-report* OR patient*-perceiv* OR patient*-percept* OR self*-report* OR selfreport*) ADJ10 (outcome* OR function* OR symptom* OR quality-of-life OR qol OR hrqol OR health-status OR improvement* OR disabilit* OR problem* OR result* OR impact*)) OR ((quality-of-life OR qol OR hrqol) ADJ3 (tool* OR measur* OR questionnaire*))).ab,ti.)

Web of Science (SCI-EXPANDED & SSCI) 153

TS=(((((heart* OR aort* OR mitral* OR tricuspid*) NEAR/2 (valve OR valvular*) NEAR/5 (replacement* OR prosthe* OR surger* OR disease* OR endocarditi* OR thrombo* OR rupture* OR

© 2024 The Authors. This is an Open Access article under the terms of the Creative Commons Attribution Non-Commercial-NoDerivs License, which permits use, distribution, and reproduction in any medium, provided that the original work is properly cited, the use is noncommercial, and no modifications or adaptations are made.

regurgitate* OR stenosis OR implant* OR repair* OR dysfunction*)) OR ((aort* OR mitral* OR tricuspid*) NEAR/2 valvuloplast*))) AND ((((patient*-report* OR patient*-perceiv* OR patient*-percept* OR self*-report* OR selfreport*) NEAR/10 (outcome* OR function* OR symptom* OR quality-of-life OR qol OR hrqol OR health-status OR improvement* OR disabilit* OR problem* OR result* OR impact*)) OR ((quality-of-life OR qol OR hrqol) NEAR/2 (tool* OR measur* OR questionnaire*))))) AND DT=(article) AND LA=(english) NOT TI=("case report*")

Cochrane CENTRAL register of Trials 65

((((heart* OR aort* OR mitral* OR tricuspid*) NEAR/3 (valve OR valvular*) NEAR/6 (replacement* OR prosthe* OR surger* OR disease* OR endocarditi* OR thrombo* OR rupture* OR regurgitate* OR stenosis OR implant* OR repair* OR dysfunction*)) OR ((aort* OR mitral* OR tricuspid*) NEAR/3 valvuloplast*)):ab,ti) AND ((((patient* NEXT report* OR patient* NEXT perceiv* OR patient* NEXT percept* OR self* NEXT report* OR selfreport*) NEAR/10 (outcome* OR function* OR symptom* OR quality NEXT of NEXT life OR qol OR hrqol OR health NEXT status OR improvement* OR disabilit* OR problem* OR result* OR impact*)) OR ((quality NEXT of NEXT life OR qol OR hrqol) NEAR/3 (tool* OR measur* OR questionnaire*))):ab,ti)NOT [conference abstract]/lim AND [english]/lim NOT ('case report'/de OR case-report*:ti)

© 2024 The Authors. This is an Open Access article under the terms of the Creative Commons Attribution Non-Commercial-NoDerivs License, which permits use, distribution, and reproduction in any medium, provided that the original work is properly cited, the use is noncommercial, and no modifications or adaptations are made.

Supplementary Table 1

Registry	Region	Valve
AVIATOR[1]	Global	Aortic
STS/ACC national database adult cardiac surgery[2]	USA	Aortic, mitral, tricuspid
STS/ACC TVT Registry[3]	USA	Aortic, mitral
TCVT Registry[4]	Europe	Aortic, mitral
GARY[5]	Germany	Aortic
Dutch national cardiothoracic database[6]	The Netherlands	Aortic, mitral, tricuspid

ACC indicates American College of Cardiology; AVIATOR, Aortic Valve Insufficiency and ascending aorta Aneurysm InternATiOnal Registry; GARY, German Aortic Valve Registry; STS, Society of Thoracic Surgeons; TCVT, TransCatheter Valve Treatment Registry; and TVT, transcatheter valve therapies.

References

1. de Heer F, Kluin J, Elkhoury G, Jondeau G, Enriquez-Sarano M, Schäfers HJ, Takkenberg JJM, Lansac E; Aortic Valve Repair Research Network Investigators. AVIATOR: an open international registry to evaluate medical and surgical outcomes of aortic valve insufficiency and ascending aorta aneurysm. *J Thorac Cardiovasc Surg*. 2019;157:2202–2211.e7. doi: 10.1016/j.jtcvs.2018.10.076
2. Bowdish ME, D'Agostino RS, Thourani VH, Schwann TA, Krohn C, Desai N, Shahian DM, Fernandez FG, Badhwar V. STS Adult Cardiac Surgery Database: 2021 update on outcomes, quality, and research. *Ann Thorac Surg*. 2021;111:1770–1780. doi: 10.1016/j.athoracsur.2021.03.043
3. Carroll JD, Mack MJ, Vemulapalli S, Herrmann HC, Gleason TG, Hanzel G, Deeb GM, Thourani VH, Cohen DJ, Desai N, et al. STS-ACC TVT registry of transcatheter aortic valve replacement. *J Am Coll Cardiol*. 2020;76:2492–2516. doi: 10.1016/j.jacc.2020.09.595
4. Di Mario C, Eltchaninoff H, Moat N, Goicolea J, Ussia GP, Kala P, Wenaweser P, Zembala M, Nickenig G, Alegria Barrero E, et al; Transcatheter Valve Treatment Sentinel Registry (TCVT) Investigators of the EURObservational Research Programme (EORP) of the European Society of Cardiology. The 2011-12 pilot European sentinel registry of transcatheter aortic valve implantation: in-hospital results in 4,571 patients. *EuroIntervention*. 2013;8:1362–1371. doi: 10.4244/EIJV8I12A209
5. Beckmann A, Hamm C, Figulla HR, Cremer J, Kuck KH, Lange R, Zahn R, Sack S, Schuler GC, Walther T, et al; GARY Executive Board. The German Aortic Valve Registry (GARY): a nationwide registry for patients undergoing invasive therapy for severe aortic valve stenosis. *Thorac Cardiovasc Surg*. 2012;60:319–325. doi: 10.1055/s-0032-1323155
6. Siregar S, Groenwold RH, Versteegh MI, Takkenberg JJ, Bots ML, van der Graaf Y, van Herwerden LA. Data resource profile: adult cardiac surgery database of the Netherlands Association for Cardio-Thoracic Surgery. *Int J Epidemiol*. 2013;42:142–149. doi: 10.1093/ije/dys241

© 2024 The Authors. This is an Open Access article under the terms of the Creative Commons Attribution Non-Commercial-NoDerivs License, which permits use, distribution, and reproduction in any medium, provided that the original work is properly cited, the use is noncommercial, and no modifications or adaptations are made.

Supplementary Figure 1

Structure of calls and focus group sessions.

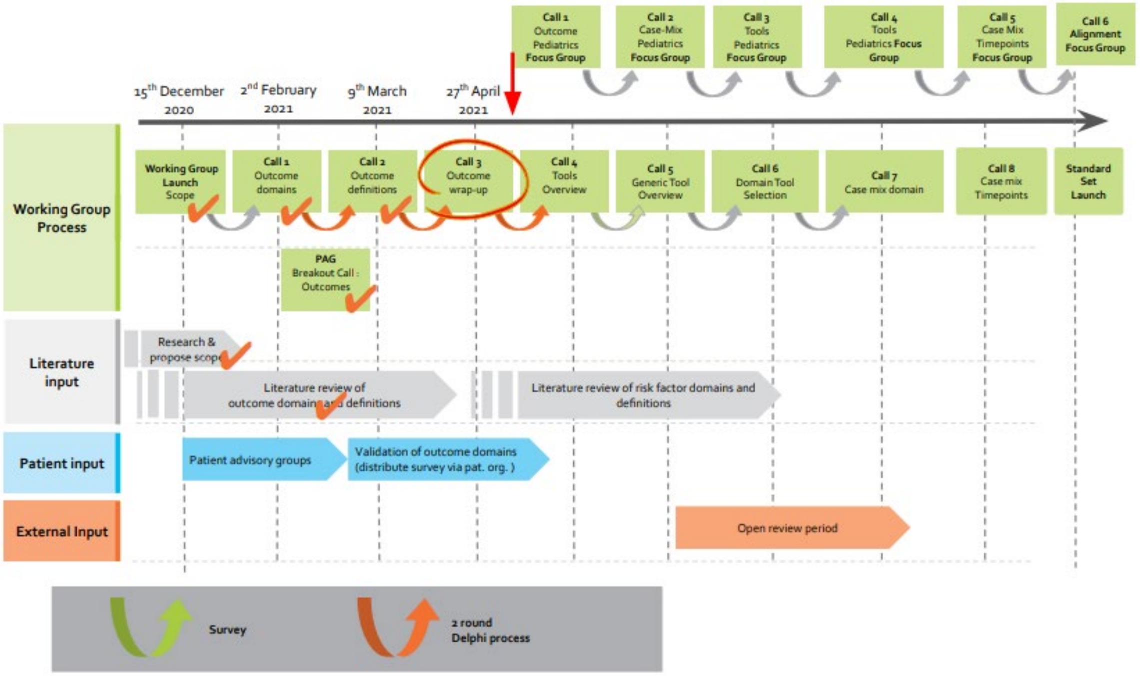

© 2024 The Authors. This is an Open Access article under the terms of the Creative Commons Attribution Non-Commercial-NoDerivs License, which permits use, distribution, and reproduction in any medium, provided that the original work is properly cited, the use is noncommercial, and no modifications or adaptations are made.

Supplementary Figure 2

Word cloud of all available abstracts.

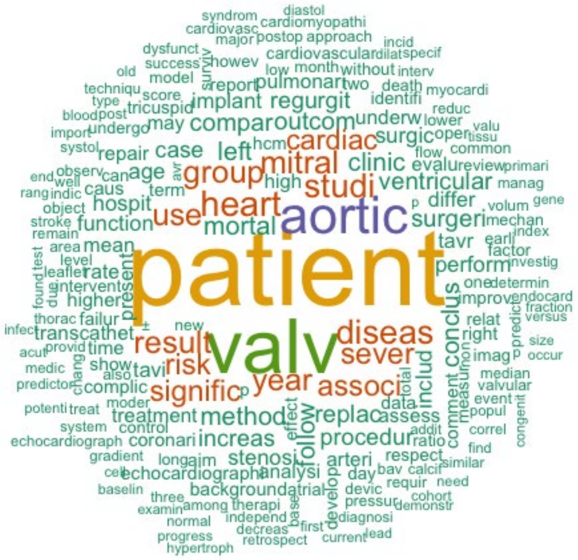

© 2024 The Authors. This is an Open Access article under the terms of the Creative Commons Attribution Non-Commercial-NoDerivs License, which permits use, distribution, and reproduction in any medium, provided that the original work is properly cited, the use is noncommercial, and no modifications or adaptations are made.
