# Supplementary material for: The First International Consortium for Health Outcomes Measurement (ICHOM) Standard Dataset for Reporting Outcomes in Heart Valve Disease: Moving From Device- to Patient-Centered Outcomes
Source: Innovations (Phila). 2025 Feb 19;20(2):133–47. doi: 10.1177/15569845241269309 (PMC12089684; doi:10.1177/15569845241269309)
Supplement: sj-pdf-2-inv-10.1177_15569845241269309 – Supplemental material for The First International Consortium for Health Outcomes Measurement (ICHOM) Standard Dataset for Reporting Outcomes in Heart Valve Disease: Moving From Device- to Patient-Centered Outcomes [file sj-pdf-2-inv-10.1177_15569845241269309.pdf]

Supplementary Figure 3

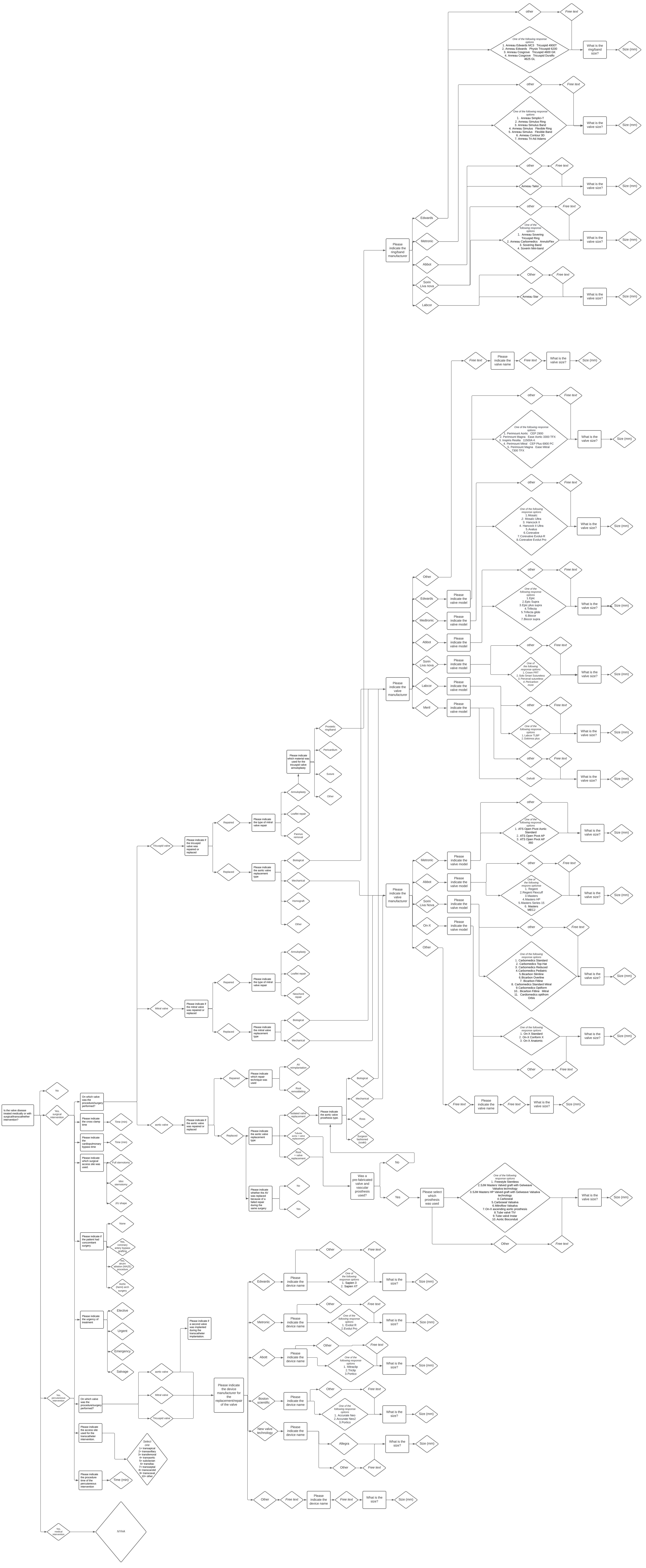

© 2024 The Authors. This is an Open Access article under the terms of the Creative Commons Attribution Non-Commercial-NoDerivs License, which permits use, distribution, and reproduction in any medium, provided that the original work is properly cited, the use is noncommercial, and no modifications or adaptations are made.
